# Association of polymorphisms in the erythropoietin gene with diabetic retinopathy: a case–control study and systematic review with meta-analysis

**DOI:** 10.1186/s12886-022-02467-y

**Published:** 2022-06-04

**Authors:** Luís Fernando Castagnino Sesti, Renan Cesar Sbruzzi, Evelise Regina Polina, Douglas dos Santos Soares, Daisy Crispim, Luís Henrique Canani, Kátia Gonçalves dos Santos

**Affiliations:** 1 Lutheran University Center of Palmas, Universidade Luterana do Brasil (ULBRA), Palmas, TO Brazil; 2 Laboratory of Human Molecular Genetics, Programa de Pós-Graduação em Biologia Celular e Molecular Aplicada à Saúde (PPGBioSaúde), Universidade Luterana do Brasil (ULBRA), Av. Farroupilha, 8001, Prédio 22, 5° andar, Canoas, RS 92425-900 Brazil; 3 Cardiovascular Research Laboratory, Hospital de Clínicas de Porto Alegre (HCPA), Porto Alegre, RS Brazil; 4 Endocrine Division, Hospital de Clínicas de Porto Alegre (HCPA), Porto Alegre, RS Brazil; 5 Department of Internal Medicine, Universidade Federal do Rio Grande do Sul (UFRGS), Porto Alegre, RS Brazil

**Keywords:** Type 2 diabetes, Diabetic retinopathy, Erythropoietin, Polymorphism, rs1617640, rs507392, rs551238

## Abstract

**Background:**

Diabetic retinopathy (DR) is characterized by ischemia, hypoxia, and angiogenesis. Erythropoietin (EPO), an angiogenic hormone, is upregulated in DR, and the association of *EPO* genetic variants with DR is still uncertain, as conflicting results have been reported. Therefore, we performed a case–control study followed by a meta-analysis to investigate whether the rs1617640, rs507392, and rs551238 polymorphisms in *EPO* gene are associated with DR.

**Methods:**

The case–control study included 1042 Southern Brazilians with type 2 diabetes (488 without DR and 554 with DR). Eligible studies for the meta-analysis were searched from electronic databases up to June 1, 2021. Pooled odds ratios (ORs) and 95% confidence intervals (CIs) were estimated for five genetic inheritance models.

**Results:**

The minor alleles of the *EPO* polymorphisms had nearly the same frequency in all groups of patients (35%), and no association was detected with DR in the case–control study. The meta-analysis included 14 independent sets of cases and controls with 9117 subjects for the rs1617640 polymorphism and nine independent sets with more than 5000 subjects for the rs507392 and rs551238 polymorphisms. The G allele of the rs1617640 polymorphism was suggestively associated with DR under the dominant (OR = 0.82, 95% CI: 0.68–0.98), heterozygous additive (OR = 0.82, 95% CI: 0.69–0.97), and overdominant (OR = 0.88, 95% CI: 0.79–0.97) models. In the subgroup analyses, the G allele was also suggestively associated with proliferative DR (PDR), non-proliferative DR (NPDR), and DR (PDR + NPDR) among patients with type 1 diabetes (T1DM) or non-Asian ancestry. After considering the Bonferroni correction for multiple comparisons, the G allele remained associated with NPDR and DR in T1DM. Regarding the rs507392 and rs551238 polymorphisms, no association was found between these variants and DR.

**Conclusion:**

Our findings provide additional support to *EPO* as a susceptibility gene for DR, with the rs1617640 polymorphism deserving further investigation.

**Supplementary Information:**

The online version contains supplementary material available at 10.1186/s12886-022-02467-y.

## Background

Diabetic retinopathy (DR) is a chronic complication of diabetes mellitus and the leading cause of blindness in working-age adults. Clinically, it is classified as non-proliferative (NPDR) and proliferative (PDR). Non-proliferative DR is characterized by microaneurysms, exudates, venous beading, and intraretinal microvascular abnormalities, whereas PDR is characterized by neovascularization, which can result in intraocular bleeding, vision loss, and retinal detachment [1]. Chronic hyperglycemia augments the activation of biochemical pathways that promote the production of inflammatory cytokines, reactive oxygen species, and vasoactive substances. Collectively, these changes disrupt the neurovascular structures and alter normal retinal function by leading to the blood–retinal barrier breakdown, pericyte loss, neuronal death, and angiogenesis [2, 3].

Erythropoietin (EPO) is a pleiotropic hormone produced mainly by the adult kidney in response to hypoxia or anemia to increase the production of red blood cells [4, 5]. Erythropoietin and its receptors are also expressed in several other tissues in response to tissue injury [5, 6], including the retinal pigment epithelium, outer and inner nuclear layers, and ganglion cell layer of the retina [4], where they exert cytoprotective effects. Experimental studies have shown that EPO has antiapoptotic, anti-inflammatory, antioxidant, and angiogenic properties [4–6], thereby protecting against retinal damage by reducing the pericyte loss, formation of acellular capillaries, and degeneration of neuroretinal layers, amongst several other features of early DR. Despite the beneficial effects of EPO administration reported in small human clinical trials and several experimental models of ocular diseases [4, 5], patients with PDR have high levels of EPO in the vitreous fluid, aqueous humor [5], postmortem retinal tissue [7], plasma [7, 8], and serum [9].

The human *EPO* gene is located on chromosome 7q22.1, contains five exons and encodes a precursor protein of 193 amino acids (https://www.ncbi.nlm.nih.gov/gene/2056). The rs1617640 (G > T), rs507392 (C > T), and rs551238 (C > A) polymorphisms in the *EPO* gene were first investigated regarding their potential association with PDR and end-stage renal disease (ESRD) in European-Americans. In that study, the T allele of the rs1617640 polymorphism was associated with PDR and ESRD in three different cohorts and had a functional role in *EPO* expression [10]. Since then, the relationship between these genetic variants and DR has been evaluated in other populations [11–20], with half of the studies reporting positive associations with either allele [11, 16, 17, 19, 20] and the other half reporting no association [12–15, 18]. Specifically, the minor G allele of the rs1617640 polymorphism was associated with risk of DR in Australian [11], Chinese [17], and Slovenian [20] patients with type 2 diabetes (T2DM). In contrast, the T allele was associated with the risk of DR in European-Americans [10] and North Indians [19] with T2DM. In relation to the rs507392 and rs551238 polymorphisms, the minor C allele was also associated with risk of DR in Australian [11] and Chinese [17] T2DM patients, whereas it was associated with protection against DR in another Chinese population of T2DM patients [16]. In addition, the C allele of the rs507392 polymorphism and the C allele of the rs551238 polymorphism were also associated with protection against DR in North Indians [19] and European-Americans [10] with T2DM, respectively.

Here, we aimed to investigate whether the rs1617640, rs507392, and rs551238 polymorphisms in the *EPO* gene are associated with DR. To address this question, we performed a case–control study in Southern Brazilians with T2DM and conducted a systematic review followed by a meta-analysis of previous studies and ours. In addition, we performed an exploratory analysis to evaluate the association between *EPO* polymorphisms and diabetic macular edema (DME).

## Methods

This study was reported in accordance with the STrengthening the REporting of Genetic Association Studies (STREGA) [21] and Preferred Reporting Items for Systematic Reviews and Meta-Analyses (PRISMA) [22] statements.

### Case–control study

#### Study population and clinical data collection

Our case–control study was carried out on a total of 1042 adult T2DM patients from Rio Grande do Sul, the southernmost Brazilian state. Most patients (*n* = 740) were enrolled between 1999 and 2010 as part of a multicenter study that aimed to investigate risk factors for chronic complications of diabetes. It mainly included the endocrinology outpatient clinics and the dialysis centers of four public tertiary care hospitals in the cities of Porto Alegre (Hospital de Clínicas de Porto Alegre—HCPA and Hospital Nossa Senhora da Conceição), Passo Fundo (Hospital São Vicente de Paulo), and Rio Grande (Hospital Universitário de Rio Grande). The remaining patients (*n* = 302) were enrolled between 2015 and 2017 in the endocrinology and the ophthalmology outpatient clinics of HCPA. This study adhered to the Declaration of Helsinki and was approved by the Human Research Ethics Committee of Universidade Luterana do Brasil—ULBRA (CAAE number: 55236216.2.0000.5349; consolidated review number: 1.553.469). All patients gave their written informed consent prior to the data and blood collection.

Type 2 diabetes was diagnosed according to the criteria of the American Diabetes Association [23], and the inclusion criteria of this study were as follows: age at the diagnosis of diabetes ≥ 30 years, no need for daily insulin treatment within the first year of diagnosis, and no previous episodes of ketoacidosis. Patients underwent a complete clinical evaluation consisting of physical examination and routine biochemical exams, including the measurement of glycated hemoglobin (HbA1c), creatinine and lipid levels, which were determined according to standard methods as described elsewhere [24]. Glomerular filtration rate (eGFR) was estimated using the CKD-EPI equation [25] and diabetic kidney disease was defined according to the KDIGO 2012 classification, as previously described [26]. A structured questionnaire was used to collect demographic data and information regarding the clinical history, such as the age at the diagnosis of diabetes, history of cigarette smoking, and presence of comorbidities, which were obtained directly by interview with the patient or from medical records. Skin color/ethnicity was self-reported and dichotomized as white or non-white (pardo or black).

Diagnosis of DR was based on either ophthalmoscopy (for patients included in the study until 2010) or retinography (for patients included between 2015 and 2017) after mydriasis by staff retinal ophthalmologists in each institution. All eye examinations were performed before DNA isolation and genotyping procedures, and patients who had any eye condition that impaired the funduscopic examination, such as severe cataract, were excluded from the study. Retinopathy was defined according to the worst affected eye and was classified as absent (no fundus abnormalities), NPDR (microaneurysms, intraretinal hemorrhages, and/or venous beading and intraretinal microvascular abnormalities), or PDR (neovascularization and/or vitreous/preretinal hemorrhage) [27]. Patients with a prior history of panretinal photocoagulation were also included in the PDR group. Overall, of the 1042 T2DM patients included in this case–control study, 488 patients did not have DR, 317 had NPDR, and 237 had PDR.

#### DNA isolation and genotyping

Genomic DNA was isolated from peripheral white blood cells using a standard salting out procedure [28]. Genotyping of *EPO* polymorphisms was performed by real-time polymerase chain reaction (PCR) using specific primers and hydrolysis probes contained in validated commercial assays for allelic discrimination (TaqMan® Genotyping Assay, ID numbers C__8786860_10, C__27168915_10, and C__2868037_10 for rs1617640, rs507392, and rs551238 polymorphisms, respectively; Thermo Fisher Scientific, Waltham, USA). Amplification reactions were carried out in a total volume of 8 μL containing 2 μL genomic DNA (10 ng/μL), 4 μL TaqMan Genotyping Master Mix (2 X) (Thermo Fisher Scientific), and 0.4 μL genotyping assay (20 X). Plates were loaded into a real-time PCR thermal cycler (StepOnePlus Real-Time PCR System; Thermo Fisher Scientific) and heated under the standard conditions recommended by the manufacturer. The genotyping was done in the Laboratory of Human Molecular Genetics at ULBRA.

A sample of each genotype was used in all PCR runs as a positive control; the investigators who performed the genotyping were blinded to the patients’ DR status (L.F.C.S. and R.C.S.) and the genotypes were read independently by two investigators (L.F.C.S. and E.R.P.). Genotyping success rate ranged from 97.8% (rs507392) to 99.1% (rs1617640), and 15% of the samples that were successfully genotyped for all three polymorphisms (152 out of 1010) were randomly selected to be re-genotyped to assess accuracy. One sample was discordant for the three polymorphisms, while another sample was discordant for only the rs551238 polymorphism. The discordant results were confirmed in a further PCR. The genotyping data generated in this study are available in a public repository (https://doi.org/10.6084/m9.figshare.16417161).

#### Statistical analysis

Continuous data are shown as the mean ± standard deviation or median (25th–75th percentiles), while categorical data are shown as absolute frequency (percentage), percentage, or relative frequency. After checking for the normal distribution using the Shapiro–Wilk test, continuous data were compared between groups by the Kruskal–Wallis test followed by the Dunn post hoc test for multiple comparisons where appropriate. Categorical data, including the genotype and allele frequencies, were compared between groups using the chi-square test followed by Bonferroni correction for multiple pairwise comparisons, if appropriate. Allele frequencies were determined by gene counting, and deviations from Hardy–Weinberg equilibrium (HWE) were also verified by the chi-square test. Statistical analyses were performed using SPSS version 18 (SPSS Inc., Chicago, USA) and WinPEPI version 11.50 [29] statistical software. Haplotype frequencies were estimated by a Bayesian method and compared between groups by a random permutation test (1000 replicates) implemented in the PHASE software version 2.1 [30, 31]. Linkage disequilibrium (LD) between the *EPO* polymorphisms was estimated and expressed in terms of *D*’ and *r*^2^ [32]. *P* values < 0.05 were considered statistically significant.

Sample size estimates were performed using the WinPEPI program. Calculations were based on the association effect sizes previously reported for *EPO* polymorphisms and DR [10, 11, 16, 17], considering a significance level of 5% and global frequencies of 0.33 (rs1617640 and rs507392) and 0.34 (rs551238) for the minor alleles, as described in the 1000 Genomes Project (https://www.ncbi.nlm.nih.gov/snp/). These estimates indicated that 1118 patients with T2DM (559 cases and 559 controls) would be needed to detect an odds ratio (OR) of as low as 1.30 (95% confidence interval [CI]): 1.02–1.65) for the association between the G allele of the rs1617640 polymorphism and DR, under the dominant model, as found in Chinese T2DM patients [17].

### Systematic review and meta-analysis

#### Search strategy and eligibility criteria

PubMed and Virtual Health Library (Biblioteca Virtual en Salud—BVS) databases were last searched on June 1, 2021, to retrieve all studies that investigated the association of at least one *EPO* gene polymorphism with DR (PDR, NPDR, or both combined), with no restriction on language. The following search terms were used: diabetes AND retinopathy AND (erythropoietin OR EPO) AND (polymorphism OR polymorphisms OR SNP OR SNPs OR variant OR variants OR mutation OR mutations OR rs1617640 OR rs507392 OR rs551238). In addition, we searched abstracts presented from 2018 to 2020 at scientific meetings of the following societies of diabetes, endocrinology, and ophthalmology: American Academy of Ophthalmology (AAO), American Diabetes Association, Endocrine Society (ENDO), European Association for the Study of Diabetes (EASD), International Society for Eye Research (ISER), and the Association for Research in Vision and Ophthalmology (ARVO).

Reference lists of the retrieved papers were also searched to identify additional studies. Studies presented in the form of a thesis or published in predatory journals were not included in our meta-analysis. Titles and abstracts of the retrieved articles were screened for eligibility, and then original studies on human subjects were reviewed in full. Authors of the papers that did not report the genotype and/or allele frequencies were contacted by e-mail. In case of no reply, the study was not included in the meta-analysis.

#### Data extraction and methodological quality assessment

Data from eligible studies were extracted independently by two of the authors (D.S.S. and K.G.S.) and disagreements were resolved by discussion and consensus. The following data were extracted and entered in an electronic spreadsheet: (i) characteristics of the study setting (name of the first author, year of publication, design type, period of inclusion, total sample size, number of cases and controls, degrees of DR, and diagnosis method of DR); (ii) characteristics of the study population (country and region, ethnicity, age, gender, type of diabetes, duration of diabetes, and presence of other diabetic complications); (iii) information regarding the polymorphisms (genotyping method, HWE in the control group, minor allele, and allele and genotype frequencies in cases and controls). Where available, haplotype frequencies and genotype/allele frequencies reported separately by the degree of DR were also registered for further analysis.

Genotype and allele data were extracted and carefully checked for accuracy. In the cases that genotype frequencies could not be clearly deduced from the paper or seemed to be incomplete, incorrect, or unclear, at least one of the authors was contacted regarding the data. Incomplete, incorrect, or unclear information regarding the DR definition was also checked with the authors of the eligible studies by e-mail. The methodological quality of each study included in our meta-analysis was assessed independently by two of the authors (D.S.S. and K.G.S.) using the Newcastle–Ottawa scoring Scale (NOS) for case–control studies [33].

#### Quantitative synthesis

The association between *EPO* polymorphisms and DR was evaluated by estimating pooled ORs and corresponding 95% CIs, using the minor allele for the rs1617640 (G), rs507392 (C), and rs551238 (C) polymorphisms as the exposure factor in the following five genetic inheritance models: (i) dominant (GG + TG vs. TT, CC + TC vs. TT, and CC + AC vs. AA); (ii) recessive (GG vs. TG + TT, CC vs. TC + TT, and CC vs. AC + AA); (iii) homozygous (GG vs. TT, CC vs. TT, and CC vs. AA) and heterozygous additive (TG vs. TT, TC vs. TT, and AC vs. AA); (iv) overdominant (TG vs. GG + TT, TC vs. CC + TT, and AC vs. CC + AA); and, (v) allele contrast (G vs. T, C vs. T, and C vs. A). Meta-analysis of haplotypes was also performed by comparing the GCC haplotype against the TTA haplotype.

Genotype frequencies were tested for HWE using the goodness-of-fit chi-square test and the overall analyses were repeated by excluding the studies in which the genotype frequencies deviated from HWE in the control group, as recommended elsewhere [34–36]. In addition, subgroup analyses were performed stratifying for the degree of DR (NPDR or PDR vs. no DR), type of diabetes (type 1 diabetes mellitus [T1DM] or T2DM), and ethnicity (Asian or non-Asian), including only the studies that met HWE. Some of the studies included in the meta-analysis enrolled two or more independent sets of cases and controls ([10, 11], present study); therefore, these groups were analyzed as separate populations.

Heterogeneity among studies was evaluated using the I^2^, τ^2^, and Q metrics, and all the individual and pooled ORs were estimated using both fixed- and random-effects models. In the presence of moderate/high heterogeneity, as defined by I^2^ ≥ 50% and *P* < 0.10 in the Q-test, the random-effects model was considered more suitable than the fixed-effects model for interpretating our meta-analysis. Otherwise, the fixed-effects model was considered as the appropriate model [34, 35]. Estimates with *P* < 0.05 were considered as evidence of suggestive association. As we performed 42 comparisons for rs1617640, 18 comparisons for rs507392, and 24 comparisons for rs551238, only the estimates with *P*-values less than 0.0012 (0.05/42), 0.0028 (0.05/18), and 0.0021 (0.05/24) for these three polymorphisms, respectively, were considered as statistically significant.

Following the recommendations of Sterne et al. [37], small-study effects were examined by visual inspection of funnel plots and formal statistical testing for the rs1617640 polymorphism, as this was the only polymorphism for which at least 10 studies were included in the meta-analysis. Rücker’s test, based on arcsine transformation of the effect measure, was used to test for funnel plot asymmetry because it is indicated for meta-analyses with binary outcomes and performs reasonably well in the presence of substantial between-study heterogeneity, as defined by τ^2^ > 0.1 [37, 38]. Statistical analyses were performed using the ‘meta’ package version 4.14–0 [39] in R version 4.0.2 [40].

## Results

### Case–control study

#### Characteristics of study population

Subjects with T2DM included in our case–control study were predominantly elderly (60.3 ± 9.5 years), female (53.3%), and white (89.0%). Subjects with DR were more often male and daily insulin users, had a longer duration of diabetes, and lower body mass index than those without this complication. In addition, patients with PDR were older and had worse renal function than those without DR (Supplementary Table 1).

#### Relationship between EPO polymorphisms and DR

Genotype frequencies were in agreement with those predicted by the Hardy–Weinberg equation for the three *EPO* polymorphisms in all T2DM groups. As the genotype and allele frequencies did not differ according to the period of inclusion in the study (Supplementary Table 2) and were quite similar in white and non-white subjects (Supplementary Table 3), all T2DM patients were analyzed together in relation to DR. As shown in Table 1, the genotype frequencies were similar between subjects with PDR, NPDR, and without DR, and the minor alleles had a frequency of approximately 0.35 in these three groups.

**Table 1:** Genotype and allele frequencies of *EPO* polymorphisms in Brazilians with type 2 diabetes

SNPs	Genotypes and alleles	All subjects	Without DR	NPDR	PDR	*P*
rs1617640﻿	Genotype	*n* = 1033	*n* = 483	*n* = 316	*n* = 234	
	TT	438 (42.4)	208 (43.1)	138 (43.7)	92 (39.3)	0.519
	TG	480 (46.5)	221 (45.7)	149 (47.1)	110 (47.0)	
	GG	115 (11.1)	54 (11.2)	29 (9.2)	32 (13.7)	
	Allele					
	T	0.66	0.66	0.67	0.63	0.299
	G	0.34	0.34	0.33	0.37	
﻿rs507392	Genotype	*n* = 1019	*n* = 473	*n* = 314	*n* = 232	
	TT	426 (41.8)	198 (41.9)	137 (43.6)	91 (39.2)	0.538
	TC	477 (46.8)	220 (46.5)	148 (47.2)	109 (47.0)	
	CC	116 (11.4)	55 (11.6)	29 (9.2)	32 (13.8)	
	Allele					
	T	0.65	0.65	0.67	0.63	0.306
	C	0.35	0.35	0.33	0.37	
﻿rs551238	Genotype	*n* = 1028	*n* = 481	*n* = 315	*n* = 232	
	AA	427 (41.5)	200 (41.6)	138 (43.8)	89 (38.4)	0.628
	AC	477 (46.4)	223 (46.3)	145 (46.0)	110 (47.4)	
	CC	124 (12.1)	58 (12.1)	32 (10.2)	33 (14.2)	
	Allele					
	A	0.65	0.65	0.67	0.62	0.266
	C	0.35	0.35	0.33	0.38	

Data are shown as absolute frequency (and percentage) or relative frequency. *SNPs* single nucleotide polymorphisms, *DR* diabetic retinopathy, *NPDR* non-proliferative DR, *PDR* proliferative DR

Among the 1010 T2DM patients successfully genotyped for the three *EPO* polymorphisms, 413 (41%) were homozygous for the major alleles, 454 (45%) were triple heterozygotes, and 110 (11%) were homozygous for the minor alleles, while the remaining 33 patients (3%) had other genotype combinations. In fact, the three polymorphisms were in strong LD (*D*’ = 0.96 and *r*^2^ = 0.90, for rs1617640 vs. rs507392; *D*’ = 0.95 and *r*^2^ = 0.88, for rs507392 vs. rs551238; and *D*’ = 0.98 and *r*^2^ = 0.93, for rs1617640 vs. rs551238). Two haplotypes accounted for > 97% of the chromosomes in our population, whereas the other six possible haplotypes had estimated individual frequencies varying from 0.01% to 1.1%. Haplotype frequencies were not significantly different between patients with PDR, NPDR, and without DR (Supplementary Table 4).

#### Relationship between EPO polymorphisms and DME

Considering that we have information on the presence or absence of DME for 139 out of the 302 T2DM patients enrolled between 2015 and 2017, an exploratory analysis was performed to examine the potential association of the three *EPO* polymorphisms with this retinal complication. Ten of the 139 patients (7.2%) had DME, with 8 patients having NPDR and 2 patients having PDR. As shown in Supplementary Table 5, the genotype, allele, and haplotype frequencies were not statistically different between those with and without DME.

### Meta-analysis

#### Study characteristics

**Fig. 1:**
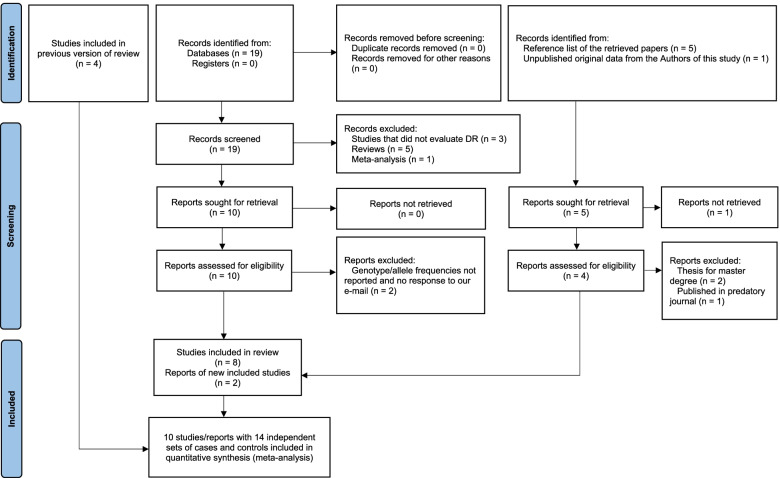
PRISMA flow diagram depicting the results of the search and selection of studies included in the meta-analysis

Nineteen non-duplicate articles were initially retrieved from PubMed and Virtual Health Library (BVS) databases, and another five studies were identified by checking the reference lists of the retrieved articles (Fig. 1). No studies were identified from the abstracts of the scientific meetings. After reviewing the titles and abstracts, 13 studies were excluded because they did not evaluate the association of *EPO* polymorphisms with DR, they did not report original data (reviews and meta-analysis), the full-text was not available, or they had not been published in peer-reviewed journals. Among the 11 full-texts reviewed, two of them were excluded because they did not report the genotype and/or the allele frequencies and the contacted author did not reply to our e-mail asking for these data [13, 15]. In addition to our case–control study, nine articles fulfilled the eligibility criteria and were included in the meta-analysis, giving 14 independent sets in total (Fig. 1) with 9117 subjects analyzed for the rs1617640 polymorphism [10–12, 14, 16–20]. Nine independent sets from seven studies, with more than 5000 subjects, were analyzed for the rs507392 and rs551238 polymorphisms ([11, 14, 16–19], present study) (Table 2).

**Table 2:** Characteristics of the studies included in the meta-analysis

First author and reference	Year	Country	Type of diabetes	*n* (cases/controls)	Cases		Controls		HWE
					**Genotypes**	**Alleles**	**Genotypes**	**Alleles**	
rs1617640				9117 (4462/4655)	TT/TG/GG	T/G	TT/TG/GG	T/G	
Tong (1) [10]	2008	USA	T2DM	613 (374/239)	150/172/52	472/276	66/127/46	259/219	Yes
Tong (2) [10]	2008	USA	T1DM	1439 (865/574)	335/419/111	1089/641	148/307/119	603/545	Yes
Tong (3) [10]	2008	USA	T1DM	520 (379/141)	139/180/60	458/300	35/78/28	148/134	Yes
Abhary (1) [11]	2010	Australia	T2DM	333 (170/163)	65/78/27	208/132	64/88/11	216/110	No
Abhary (2) [11]	2010	Australia	T1DM	167 (102/65)	40/44/18	124/80	24/30/11	78/52	Yes
Balasubbu [12]	2010	India	T2DM	702 (344/358)	31/163/150	225/463	30/171/157	231/485	Yes
Yang [14]	2014	China	T2DM	491 (211/280)	146/55/10	347/75	182/82/16	446/114	Yes
Song [16]	2015	China	T2DM	782 (444/338)	293/138/13	724/164	225/98/15	548/128	Yes
Fan [17]	2016	China	T2DM	1193 (397/796)	208/161/28	577/217	468/302/26	1238/354	No
Montesanto [18]	2018	Italy	T2DM	433 (107/326)	51/42/14	144/70	140/145/41	425/227	Yes
Kaur [19]	2021	India	T2DM	614 (302/312)	125/129/48	379/225	122/116/74	360/264	No
Mankoc Ramus [20]	2021	Slovenia	T2DM	797 (217/580)	70/96/51	236/198	180/305/95	665/495	Yes
Sesti (1)	Present study	Brazil	T2DM	731 (422/309)	183/194/45	560/284	132/147/30	411/207	Yes
Sesti (2)	Present study	Brazil	T2DM	302 (128/174)	47/65/16	159/97	76/74/24	226/122	Yes
rs507392				5023 (2281/2742)	TT/TC/CC	T/C	TT/TC/CC	T/C	
Abhary (1) [11]	2010	Australia	T2DM	332 (170/162)	65/78/27	208/132	63/88/11	214/110	No
Abhary (2) [11]	2010	Australia	T1DM	167 (102/65)	40/44/18	124/80	24/30/11	78/52	Yes
Yang [14]	2014	China	T2DM	496 (216/280)	141/65/10	347/85	181/81/18	443/117	No
Song [16]	2015	China	T2DM	782 (444/338)	281/149/14	711/177	217/97/24	531/145	No
Fan [17]	2016	China	T2DM	1193 (397/796)	202/161/34	565/229	463/305/28	1231/361	No
Montesanto [18]	2018	Italy	T2DM	420 (104/316)	48/43/13	139/69	130/146/40	406/226	Yes
Kaur [19]	2021	India	T2DM	614 (302/312)	138/124/40	400/204	132/106/74	370/254	No
Sesti (1)	Present study	Brazil	T2DM	722 (418/304)	181/192/45	554/282	126/148/30	400/208	Yes
Sesti (2)	Present study	Brazil	T2DM	297 (128/169)	47/65/16	159/97	72/72/25	216/122	Yes
rs551238				5031 (2279/2752)	AA/AC/CC	A/C	AA/AC/CC	A/C	
Abhary (1) [11]	2010	Australia	T2DM	333 (170/163)	65/78/27	208/132	64/88/11	216/110	No
Abhary (2) [11]	2010	Australia	T1DM	167 (102/65)	40/44/18	124/80	24/30/11	78/52	Yes
Yang [14]	2014	China	T2DM	494 (216/278)	141/65/10	347/85	182/79/17	443/113	No
Song [16]	2015	China	T2DM	774 (439/335)	286/140/13	712/166	219/92/24	530/140	No
Fan [17]	2016	China	T2DM	1193 (397/796)	203/156/38	562/232	452/299/45	1203/389	Yes
Montesanto [18]	2018	Italy	T2DM	428 (106/322)	51/42/13	144/68	138/143/41	419/225	Yes
Kaur [19]	2021	India	T2DM	614 (302/312)	130/125/47	385/219	123/117/72	363/261	No
Sesti (1)	Present study	Brazil	T2DM	731 (419/312)	181/188/50	550/288	129/150/33	408/216	Yes
Sesti (2)	Present study	Brazil	T2DM	297 (128/169)	46/66/16	158/98	71/73/25	215/123	Yes

*SNP* single nucleotide polymorphism, *T2DM* type 2 diabetes mellitus, *T1DM* type 1 diabetes mellitus, *HWE* Hardy–Weinberg equilibrium

Studies included in our meta-analysis were case–control or cross-sectional studies, and most of them enrolled T2DM patients. Most independent sets of cases and controls were composed predominantly of white subjects of European ancestry ([10, 11, 18, 20], present study), while five studies were carried out on Indian [12, 19] and Chinese [14, 16, 17] populations (Table 2). According to NOS, all the previous studies included in the meta-analysis were of good quality, with the total scores ranging from 7 to 9 (Supplementary Table 6).

#### Quantitative pooled analyses of rs1617640 polymorphism

Pooled estimates for the overall association between the rs1617640 polymorphism and DR revealed a moderate to high between-study heterogeneity (I^2^ = 57–76%) in almost all genetic models including all the 14 independent subject sets (Supplementary Table 7). We then sought to identify its source by excluding set #2 of Tong et al. [10], which had the highest weight in the analyses (18–27%), and the three sets with controls deviating from HWE [set #1 of 11, 17, 19]. Exclusion of these studies from the meta-analysis markedly reduced the heterogeneity (I^2^ = 12–39%), while the pooled estimates became suggestively significant with the removal of the studies that did not meet the HWE in half of the genetic models. Although the funnel plots were asymmetric with set #2 by Tong et al. [10] and some others lying outside the boundary line, this asymmetry was not confirmed by statistical analysis (Supplementary Table 7). The forest and funnel plots are provided in Supplementary Figs. 1–48.

Table 3 shows the overall and subgroup analyses after removing the three subject sets in which the genotype frequencies were not in agreement with HWE. The G allele was suggestively associated with a reduced risk of DR under the dominant, heterozygous additive, and overdominant genetic models. Following the standard recommendations for meta-analysis of genetic association studies, we also stratified the analyses by the degree of DR, type of diabetes, and ethnicity including only the studies whose genotype frequencies met the HWE in the control group. Again, the G allele was suggestively associated with both PDR and NPDR, under the overdominant and recessive models, respectively, and with DR among patients with T1DM under all the genetic models. Regarding the subjects of non-Asian ancestry, the G allele was suggestively associated with the reduced risk of DR under the dominant, heterozygous additive, and overdominant models. However, only the association of the GG genotype with NPDR and the association of the G allele with DR in T1DM remained statistically significant after taking into account multiple testing (*P* < 0.0012). Moreover, no association between the rs1617640 polymorphism and DR was detected in patients with T2DM or Asian ancestry (Table 3). The forest plots are shown in Supplementary Figs. 49–84.

**Table 3:** Pooled estimates for the association between the *EPO* rs1617640 polymorphism and DR

Subgroup and genetic model	*n* (cases/controls)	Heterogeneity		Effect model	Pooled OR (95% CI)	P**
		**I**^**2**^** (%)**	P*			
Overall (*n* = 11)						
Dominant (GG + TG vs. TT)	6977 (3593/3384)	62	0.003	Random	**0.82 (0.68–0.98)**	0.029
Recessive (GG vs. TG + TT)	6977 (3593/3384)	55	0.013	Random	0.88 (0.71–1.10)	0.273
Homozygous additive (GG vs. TT)	3845 (2025/1820)	65	0.002	Random	0.78 (0.58–1.04)	0.088
Heterozygous additive (TG vs. TT)	5855 (3053/2802)	50	0.030	Random	**0.82 (0.69–0.97)**	0.018
Overdominant (TG vs. GG + TT)	6977 (3593/3384)	7	0.375	Fixed	**0.88 (0.79–0.97)**	0.009
Allele contrast (G vs. T)	13,954 (7186/6768)	67	< 0.001	Random	0.88 (0.77–1.01)	0.075
PDR (*n* = 8)						
Dominant (GG + TG vs. TT)	4843 (2130/2713)	74	< 0.001	Random	0.79 (0.60–1.04)	0.089
Recessive (GG vs. TG + TT)	4843 (2130/2713)	65	0.006	Random	0.90 (0.68–1.21)	0.490
Homozygous additive (GG vs. TT)	2592 (1186/1406)	75	< 0.001	Random	0.76 (0.50–1.15)	0.188
Heterozygous additive (TG vs. TT)	3929 (1730/2199)	66	0.004	Random	0.78 (0.61–1.01)	0.058
Overdominant (TG vs. GG + TT)	4843 (2130/2713)	38	0.127	Fixed	**0.82 (0.73–0.93)**	0.002
Allele contrast (G vs. T)	9686 (4260/5426)	77	< 0.001	Random	0.88 (0.73–1.06)	0.181
NPDR (*n* = 4)						
Dominant (GG + TG vs. TT)	2438 (1043/1395)	72	0.014	Random	0.90 (0.65–1.26)	0.552
Recessive (GG vs. TG + TT)	2438 (1043/1395)	25	0.260	Fixed	**0.63 (0.49–0.82)**	**0.0006**
Homozygous additive (GG vs. TT)	1329 (560/769)	60	0.055	Random	0.69 (0.41–1.15)	0.152
Heterozygous additive (TG vs. TT)	2152 (945/1207)	56	0.076	Random	0.94 (0.71–1.25)	0.689
Overdominant (TG vs. GG + TT)	2438 (1043/1395)	0	0.633	Fixed	1.02 (0.87–1.20)	0.804
Allele contrast (G vs. T)	4876 (2086/2790)	73	0.010	Random	0.90 (0.69–1.16)	0.404
T2DM (*n* = 8)						
Dominant (GG + TG vs. TT)	4851 (2247/2604)	35	0.152	Fixed	0.90 (0.79–1.02)	0.100
Recessive (GG vs. TG + TT)	4851 (2247/2604)	31	0.180	Fixed	1.00 (0.85–1.18)	0.993
Homozygous additive (GG vs. TT)	2777 (1322/1455)	35	0.151	Fixed	0.91 (0.74–1.12)	0.367
Heterozygous additive (TG vs. TT)	4076 (1896/2180)	32	0.174	Fixed	0.89 (0.78–1.02)	0.100
Overdominant (TG vs. GG + TT)	4851 (2247/2604)	23	0.243	Fixed	0.91 (0.81–1.03)	0.124
Allele contrast (G vs. T)	9702 (4494/5208)	38	0.124	Fixed	0.95 (0.87–1.04)	0.251
T1DM (*n* = 3)						
Dominant (GG + TG vs. TT)	2126 (1346/780)	4	0.353	Fixed	**0.58 (0.48–0.70)**	** < 0.0001**
Recessive (GG vs. TG + TT)	2126 (1346/780)	24	0.269	Fixed	**0.63 (0.50–0.80)**	**0.0001**
Homozygous additive (GG vs. TT)	1068 (703/365)	41	0.183	Fixed	**0.47 (0.36–0.62)**	** < 0.0001**
Heterozygous additive (TG vs. TT)	1779 (1157/622)	0	0.569	Fixed	**0.62 (0.50–0.76)**	** < 0.0001**
Overdominant (TG vs. GG + TT)	2126 (1346/780)	0	0.841	Fixed	**0.80 (0.67–0.96)**	0.016
Allele contrast (G vs. T)	4252 (2692/1560)	30	0.241	Fixed	**0.69 (0.61–0.78)**	** < 0.0001**
Non-Asian (*n* = 8)						
Dominant (GG + TG vs. TT)	5002 (2594/2408)	68	0.003	Random	**0.78 (0.62–0.99)**	0.037
Recessive (GG vs. TG + TT)	5002 (2594/2408)	67	0.004	Random	0.90 (0.67–1.21)	0.474
Homozygous additive (GG vs. TT)	2577 (1382/1195)	75	< 0.001	Random	0.77 (0.53–1.13)	0.186
Heterozygous additive (TG vs. TT)	4241 (2227/2014)	53	0.037	Random	**0.77 (0.63–0.95)**	0.013
Overdominant (TG vs. GG + TT)	5002 (2594/2408)	4	0.399	Fixed	**0.83 (0.74–0.94)**	0.002
Allele contrast (G vs. T)	10,004 (5188/4816)	74	< 0.001	Random	0.87 (0.73–1.04)	0.122
Asian (*n* = 3)						
Dominant (GG + TG vs. TT)	1975 (999/976)	0	0.681	Fixed	0.94 (0.76–1.17)	0.580
Recessive (GG vs. TG + TT)	1975 (999/976)	0	0.571	Fixed	0.92 (0.71–1.20)	0.547
Homozygous additive (GG vs. TT)	1268 (643/625)	0	0.785	Fixed	0.82 (0.55–1.21)	0.306
Heterozygous additive (TG vs. TT)	1614 (826/788)	0	0.602	Fixed	0.97 (0.78–1.22)	0.804
Overdominant (TG vs. GG + TT)	1975 (999/976)	0	0.599	Fixed	1.00 (0.82–1.20)	0.959
Allele contrast (G vs. T)	3950 (1998/1952)	0	0.743	Fixed	0.95 (0.81–1.10)	0.467

*n* = number of independent sets of cases and controls. *Computed by Q-test. Statistically suggestive association estimates are shown in bold, considering the most appropriate model for each analysis (fixed- or random-effects). **P-values that reached the threshold for statistical significance after considering the multiple comparisons (< 0.05/42 = 0.0012) are shown in bold. *OR* odds ratio, *95% CI* 95% confidence interval, *PDR* proliferative DR, *NPDR* non-proliferative DR

#### Quantitative pooled analyses of rs507392 and rs551238 polymorphisms

In relation to the rs507392 polymorphism, five of the nine subject sets initially eligible for the meta-analysis did not meet the HWE [set #1 of 11, 14, 16, 17, 19]. Removing these studies from the meta-analysis eliminated the between-study heterogeneity, while the association estimates remained statistically non-significant. With fewer data available, subgroup analyses including only the studies that met the HWE were restricted to T2DM patients (who were all of non-Asian ancestry), and no association was observed in this group (Table 4 and Supplementary Figs. 85–102).

**Table 4:** Pooled estimates for the association between the *EPO* rs507392 polymorphism and DR

Subgroup and genetic model	*n* (cases/controls)	Heterogeneity		Effect model	Pooled OR (95% CI)
		**I**^**2**^** (%)**	P*		
Overall (*n* = 9)					
Dominant (CC + TC vs. TT)	5023 (2281/2742)	2	0.414	Fixed	1.04 (0.93–1.17)
Recessive (CC vs. TC + TT)	5023 (2281/2742)	79	< 0.001	Random	0.99 (0.63–1.54)
Homozygous additive (CC vs. TT)	3029 (1360/1669)	76	< 0.001	Random	1.00 (0.64–1.55)
Heterozygous additive (TC vs. TT)	4545 (2064/2481)	0	0.622	Fixed	1.06 (0.94–1.20)
Overdominant (TC vs. CC + TT)	5023 (2281/2742)	26	0.216	Fixed	1.06 (0.94–1.19)
Allele contrast (C vs. T)	10,046 (4562/5484)	60	0.010	Random	1.00 (0.86–1.16)
Only in HWE (*n* = 4)					
Dominant (CC + TC vs. TT)	1606 (752/854)	0	0.568	Fixed	0.96 (0.78–1.18)
Recessive (CC vs. TC + TT)	1606 (752/854)	0	0.921	Fixed	1.00 (0.73–1.37)
Homozygous additive (CC vs. TT)	866 (408/458)	0	0.986	Fixed	0.98 (0.70–1.37)
Heterozygous additive (TC vs. TT)	1408 (660/748)	0	0.414	Fixed	0.95 (0.77–1.19)
Overdominant (TC vs. CC + TT)	1606 (752/854)	7	0.360	Fixed	0.96 (0.78–1.18)
Allele contrast (C vs. T)	3212 (1504/1708)	0	0.888	Fixed	0.98 (0.84–1.14)
T2DM (*n* = 3)					
Dominant (CC + TC vs. TT)	1439 (650/789)	0	0.369	Fixed	0.96 (0.77–1.20)
Recessive (CC vs. TC + TT)	1439 (650/789)	0	0.789	Fixed	0.99 (0.71–1.39)
Homozygous additive (CC vs. TT)	773 (350/423)	0	0.929	Fixed	0.98 (0.69–1.41)
Heterozygous additive (TC vs. TT)	1270 (576/694)	29	0.247	Fixed	0.96 (0.76–1.21)
Overdominant (TC vs. CC + TT)	1439 (650/789)	36	0.207	Fixed	0.97 (0.78–1.20)
Allele contrast (C vs. T)	2878 (1300/1578)	0	0.729	Fixed	0.98 (0.83–1.15)

*n* = number of independent sets of cases and controls. *Computed by Q-test. *OR* odds ratio, *95% CI* 95% confidence interval, *HWE* Hardy–Weinberg equilibrium

Regarding the rs551238 polymorphism, the genotype frequencies in controls were not in agreement with HWE in four of the nine subject sets [set #1 of 11, 14, 16, 19]. The overall pooled analyses including or excluding these studies revealed no association between the rs551238 and DR in any genetic model, even among T2DM patients or non-Asians (Table 5 and Supplementary Figs. 103–126).

**Table 5:** Pooled estimates for the association between the *EPO* rs551238 polymorphism and DR

Subgroup and genetic model	*n* (cases/controls)	Heterogeneity		Effect model	Pooled OR (95% CI)
		**I**^**2**^** (%)**	P*		
Overall (*n* = 9)					
Dominant (CC + AC vs. AA)	5031 (2279/2752)	0	0.595	Fixed	1.02 (0.91–1.15)
Recessive (CC vs. AC + AA)	5031 (2279/2752)	69	0.001	Random	0.98 (0.68–1.40)
Homozygous additive (CC vs. AA)	3056 (1375/1681)	66	0.003	Random	0.98 (0.68–1.40)
Heterozygous additive (AC vs. AA)	4520 (2047/2473)	0	0.698	Fixed	1.04 (0.92–1.18)
Overdominant (AC vs. CC + AA)	5031 (2279/2752)	9	0.362	Fixed	1.04 (0.92–1.16)
Allele contrast (C vs. A)	10,062 (4558/5504)	43	0.084	Fixed	1.01 (0.92–1.10)
Only in HWE (*n* = 5)					
Dominant (CC + AC vs. AA)	2816 (1152/1664)	20	0.288	Fixed	1.07 (0.92–1.25)
Recessive (CC vs. AC + AA)	2816 (1152/1664)	13	0.329	Fixed	1.20 (0.93–1.55)
Homozygous additive (CC vs. AA)	1625 (656/969)	22	0.277	Fixed	1.22 (0.94–1.60)
Heterozygous additive (AC vs. AA)	2526 (1017/1509)	10	0.348	Fixed	1.03 (0.88–1.22)
Overdominant (AC vs. CC + AA)	2816 (1152/1664)	0	0.405	Fixed	1.00 (0.85–1.17)
Allele contrast (C vs. A)	5632 (2304/3328)	24	0.260	Fixed	1.08 (0.96–1.22)
T2DM (*n* = 4)					
Dominant (CC + AC vs. AA)	2649 (1050/1599)	36	0.194	Fixed	1.08 (0.92–1.27)
Recessive (CC vs. AC + AA)	2649 (1050/1599)	33	0.214	Fixed	1.22 (0.94–1.59)
Homozygous additive (CC vs. AA)	1532 (598/934)	38	0.184	Fixed	1.25 (0.94–1.65)
Heterozygous additive (AC vs. AA)	2388 (933/1455)	29	0.238	Fixed	1.04 (0.88–1.24)
Overdominant (AC vs. CC + AA)	2649 (1050/1599)	22	0.278	Fixed	1.01 (0.86–1.19)
Allele contrast (C vs. A)	5298 (2100/3198)	40	0.171	Fixed	1.09 (0.97–1.24)
Non-Asian (*n* = 4)					
Dominant (CC + AC vs. AA)	1623 (755/868)	0	0.537	Fixed	0.96 (0.78–1.18)
Recessive (CC vs. AC + AA)	1623 (755/868)	0	0.881	Fixed	1.02 (0.75–1.38)
Homozygous additive (CC vs. AA)	887 (415/472)	0	0.964	Fixed	0.99 (0.72–1.38)
Heterozygous additive (AC vs. AA)	1416 (658/758)	1	0.386	Fixed	0.95 (0.76–1.18)
Overdominant (AC vs. CC + AA)	1623 (755/868)	13	0.329	Fixed	0.95 (0.78–1.17)
Allele contrast (C vs. A)	3246 (1510/1736)	0	0.857	Fixed	0.98 (0.84–1.14)

*n* = number of independent sets of cases and controls. *Computed by Q-test. *OR* odds ratio, *95% CI* 95% confidence interval, *HWE* Hardy–Weinberg equilibrium

#### Quantitative pooled analyses of EPO haplotypes

**Fig. 2:**
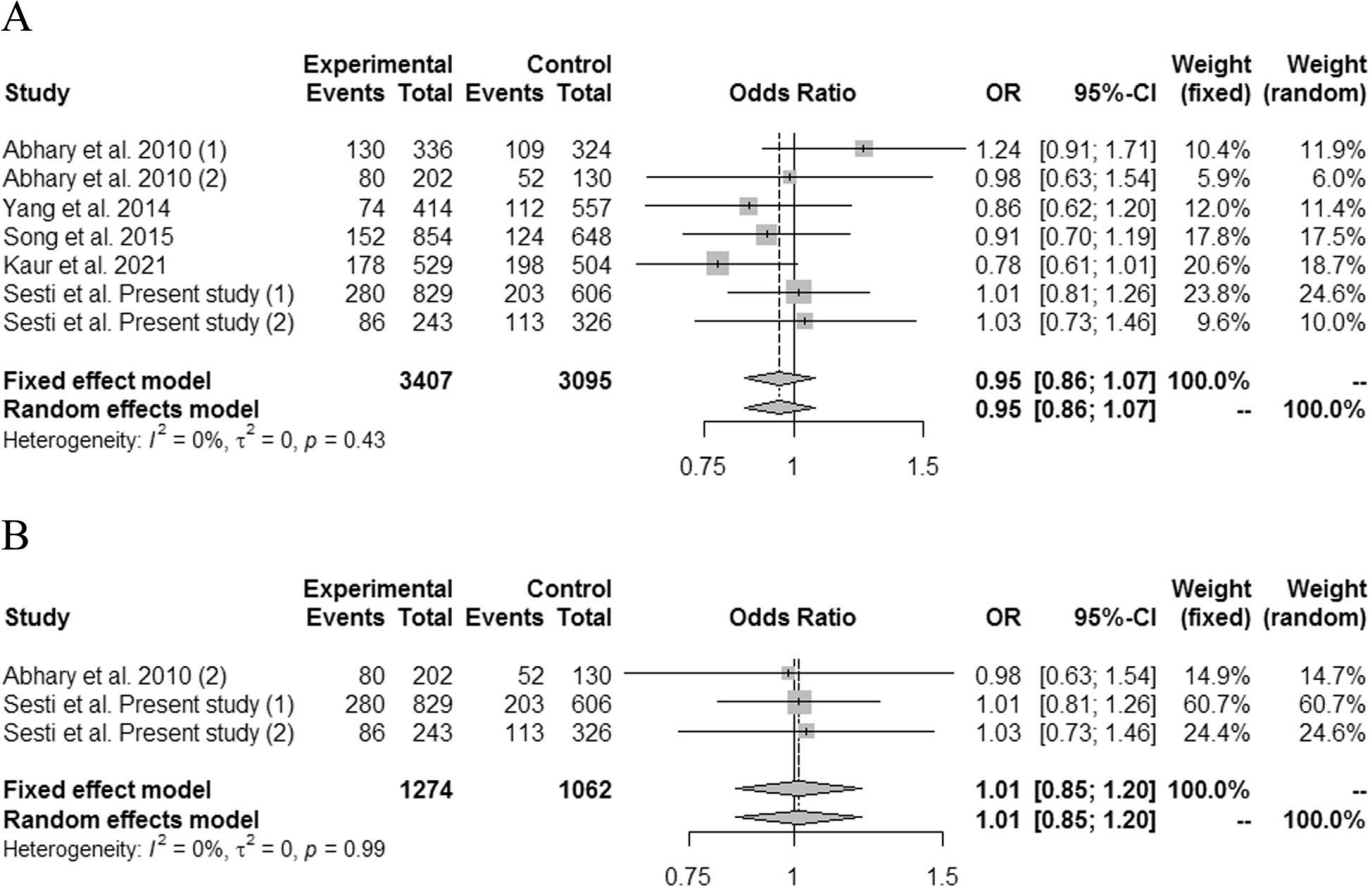
Forest plots of the association between the *EPO* polymorphisms and diabetic retinopathy (GCC haplotype vs. TTA haplotype) in the overall group analysis (**a**) and in the analysis including only the sets with controls in Hardy–Weinberg equilibrium (**b**)

The combined analysis, including seven sets of cases and controls, showed that the haplotype carrying the minor alleles (GCC) was not associated with DR in comparison to the haplotype carrying the major alleles (TTA), regardless of whether the genotype frequencies were in HWE in the control groups (Fig. 2).

## Discussion

In our case–control study, we did not find evidence of an association between *EPO* gene polymorphisms and DR in patients with T2DM from Southern Brazil. However, the meta-analysis showed that the G allele of the rs1617640 was associated with protection for NPDR under the recessive model. In the subgroup analyses by type of diabetes and ancestry, the G allele was also associated with a decreased risk of DR (PDR + NPDR) among patients with T1DM. No other statistically significant associations were detected after taking into account the multiple comparisons.

Regarding the rs1617640 polymorphism, the findings of our case–control study are in accordance with most of the previous individual studies, which reported no association between this genetic variant and DR in Indian [12], Chinese [14, 16], and Italian T2DM patients [18], as well as in five different cohorts of subjects of white European ancestry with T1DM [11, 13, 15]. The rs1617640 polymorphism was also not associated with time to development of severe DR in a large cohort of T1DM patients followed for over 15 years (from the WESDR + DCCT/EDIC studies) [15]. However, other studies have found opposing results [10, 11, 17, 19, 20]. The T allele was associated with an increased risk of PDR in three European-American cohorts of T2DM and T1DM patients from different geographic areas in the United States [10] and with DR in North Indians with T2DM [19]. On the other hand, the G allele was associated with the increased risk of DR in T2DM patients from Australia [11], China [17], and Slovenia [20]. Thus, differences in ethnicity and type of diabetes do not seem to explain the discrepancies between these studies.

When all the available genotype data were pooled in the meta-analysis, the between-heterogeneity was substantial, and the random-effects model revealed no association between the rs1617640 polymorphism and DR. The asymmetry seen in the funnel plots, and not confirmed by statistical test, can be attributed to neither publication bias nor small study effects [34–37] because most of the studies did not find an association between the rs1617640 variant and DR, and two of the subject sets that lied outside the majority of funnel plots and had shown the strongest association had sample sizes of more than 1100 individuals [set #2 of 10, 17]. Apart from this, genotype frequencies were not in HWE among controls in three studies [set #1 of 11, 17, 19]. Departures from HWE may occur due to several reasons other than genotyping errors, such as population stratification and selection bias in the enrollment of controls [34, 35]. Despite these considerations, HWE has been used as the main parameter of post-genotyping quality control in association studies.

Therefore, following the standard recommendations for meta-analyses of gene-disease associations [34–36], we removed the sets in which genotype frequencies were in Hardy–Weinberg disequilibrium to repeat the overall analysis and perform the subgroup analyses for the rs1617640 polymorphism to identify the possible causes of between-study heterogeneity. Heterogeneity was still moderate to high for most genetic models in the PDR, NPDR, and non-Asian subgroup analyses, while it was low or null among T2DM, T1DM, and Asian patients. The subject set #2 of Tong et al. [10], which contributed 20.6% of the total sample size in the overall meta-analysis, seemed to be the main factor contributing to the heterogeneity across the studies and also contributed to statistically significant and suggestive associations between the rs1617640 polymorphism and DR in PDR, NPDR, T1DM, and non-Asian subgroups.

An issue that may raise some criticism regarding the results is the fact that our case–control study did not detect an association between the rs1617640 polymorphism and DR, whereas our meta-analysis revealed an association of the G allele with a reduced risk of DR. This is not unexpected. First, the meta-analysis involves a larger number of subjects, therefore it is more powerful than a single study to detect an association of low magnitude. Second, the association with either one of the two alleles, or the lack of it, may be population-specific. Third, although the type of diabetes did not seem to explain the discrepant results across the individual studies, the meta-analysis showed that the G allele of the rs1617640 polymorphism was associated with a decreased risk of DR under almost all the genetic models in T1DM patients, while no association was observed in T2DM patients. Thus, the results of our case–control study are not necessarily in conflict with those obtained in the meta-analysis.

Moreover, the findings of our meta-analysis regarding the rs1617640 polymorphism are in line with those reported in a previous study, in which the TT genotype was associated with an increased risk of PDR + ESRD, as compared to the GG genotype, with a similar magnitude of association [41]. In their analyses, without the cohorts of Tong et al. [10], no association between the rs1617640 polymorphism and DR was observed in any genetic model, as well as in T2DM and Asian populations [41]. Supplementary Table 8 summarizes the main differences between our meta-analysis and the previous one [41].

Although both meta-analyses indicate the existence of an association between the rs1617640 polymorphism and DR, the actual biological model that describes such relationship is yet to be defined. The G allele was associated with DR under a recessive model in four studies [11, 17, 19, 20]. However, in our overall meta-analysis, the G allele was suggestively associated with a decreased risk of DR under the dominant, heterozygous additive, and overdominant genetic models. This is in line with the original report by Tong et al. [10], which suggested an additive allele–dosage effect for the rs1617640 polymorphism. To the best of our knowledge, those authors were the only ones who performed functional assays and prediction analysis to evaluate the effect of the rs1617640 variant on gene expression. The T allele markedly increased the *EPO* expression in cultured human embryonic kidney (HEK) 293 cells, and the computational analysis suggested that the T allele creates a binding site for the transcription factors EVI1/MEL1 and AP1, which likely accounted for the enhanced expression as compared with the G allele. Moreover, vitreous levels of EPO were much higher in non-diabetic subjects with the TT genotype than in those with the GG genotype [10]. Taken together, these findings suggest that high levels of EPO are associated with DR, especially PDR, and the T allele of the rs1617640 is likely a risk factor for DR as it increases the gene expression. This is consistent with experimental evidence showing that exogenous EPO protects against early DR, but it is detrimental in PDR [4–6].

In relation to the rs507392 and rs551238 polymorphisms, available data are scarcer [10, 11, 14, 16–19]. Not all the studies discussed so far have investigated the association of these two genetic variants with DR and, among those that examined such an association, not all reported the genotype data. The findings of the previous studies are varied, even in populations with the same ethnicity. Under the recessive and homozygous additive models, the C allele of both polymorphisms was strongly associated with an increased risk of DR in Australian [11] and Chinese [17] T2DM patients, whereas it was strongly associated with a decreased risk of DR in another population of Chinese T2DM patients [16]. In addition, the C allele of the rs507392 polymorphism was associated with a decreased risk of DR in North Indian T2DM patients, whereas the C allele of the rs551238 was not associated with this complication [19]. In contrast, the C allele of the rs551238 polymorphism was less frequent in patients with DR than in those without this complication, while the rs507392 polymorphism was not associated with DR in the cohort of Utahns (USA) of European ancestry with T2DM [10]. Similar to our case–control study, the rs507392 and rs551238 polymorphisms were not associated with DR in T1DM patients from Australia [11] and in T2DM patients from China [14] and Italy [18]. Our meta-analysis, including either all the studies or only the subject sets in which the control groups met the HWE, revealed no association of the rs507392 and rs551238 variants with DR.

Although the rs1617640, rs507392, and rs551238 polymorphisms were in high LD with each other in T1DM and T2DM patients ([10, 11, 14, 16, 19], present study), findings regarding an additional combined effect of the three *EPO* polymorphisms in the susceptibility of DR are also inconclusive. No evidence of an association between *EPO* haplotypes and DR was found in our case–control study as well as in two Chinese T2DM populations [14, 16]. However, the GCC haplotype was reported to be independently associated with an increased risk of DR under a recessive model in T2DM, but not in T1DM, in a white Australian population [11]. In another study of Chinese T2DM patients, the strongest relationship was observed for the carriership of at least one copy of the minor allele of each polymorphism (GCC) in comparison to the homozygosity for the three major alleles [17]. It is worth noting that risk and protective haplotypes were identified in the cohorts studied by Tong et al. [10], and the main difference between them was the rs1617640 polymorphism. Risk haplotypes carried the T allele, while the G allele was present in the protective haplotypes, irrespective of the alleles at the other two polymorphisms [10]. On the other hand, a recent study on North Indians with T2DM reported that the main source of the association between the TTA haplotype and DR was the T allele of the rs507392 polymorphism [19]. However, our meta-analysis detected no association between the GCC haplotype and DR.

In general, the studies included in our meta-analysis can be considered of good quality as suggested by the scoring scale used for this purpose (NOS). However, specific guidelines for genetic association studies have focused on the HWE test in controls as a means of assessing study quality [34–36] and on the phenotyping, blinding, validity of genotyping method, and population stratification [21, 34]. A critical aspect related to the methodological quality of the previous studies is the lack of blinding and re-genotyping as quality control procedures in half of them [16, 17, 19, 20]. In the other studies, at least one procedure to improve the genotyping accuracy was reported, such as the re-genotyping of part of the samples by sequencing [10], sequencing of some samples for each genotype at each polymorphism [11], and genotype reading by two investigators blinded to the sample phenotypes [12]. Other authors, who genotyped the samples for *EPO* polymorphisms using the Sequenom technology, described a battery of quality control tests [14, 18]. In addition, retinopathy grading was reported to have been performed without prior knowledge of genotypes in one study [10].

Population stratification is unlikely to have been a confounding factor [21, 42] in the previous studies since the authors enrolled subjects from populations with a majority ethnic group (> 90%) [10, 11, 14, 16, 17, 19, 20], used the ancestry from a given region as one of the inclusion criteria [10, 18] or matched the cases and controls by ethnicity [12]. In the study by Abhary et al. [11], there was no difference in the allele frequencies of *EPO* polymorphisms among white subjects of European ancestry and non-white subjects of Asian and Middle Eastern ancestry. In our case–control study, the genotype and allele frequencies were virtually identical in white and non-white subjects.

On the other hand, the unavailability of all genotype data that could be incorporated in the quantitative synthesis is the main limitation of our meta-analysis. Some of the previous original studies were published without reporting the genotype frequencies of the polymorphisms under investigation. In addition, data reported in some papers were unclear, inaccurate, or did not match the sample size described in the text or in the tables. Despite our attempt to obtain all the missing genotype data by e-mail, they were still missing for the rs1617640 [13, 15], rs507392, and rs551238 [10] polymorphisms. Hence, we included most, but not all the previous studies that examined the association of *EPO* polymorphisms with DR and met the screening criteria. Although we could perform subgroup analyses by the severity of DR, considering NPDR and PDR as separate outcomes, it was also limited by the lack of this information in most of the eligible studies. In future studies, authors, as well as the reviewers and editors, should be aware that the complete description of genotype frequencies and outcomes is essential to allow for comparisons across the studies and perform pooled association analyses.

Finally, DME is another retinal complication of diabetes that is also one of the leading causes of visual loss and can occur at any stage of DR [43]. Intravitreal levels of EPO have been found to be increased in patients with DME and both clinical and experimental evidence suggest that EPO could be beneficial for the treatment of DME [5]. Therefore, it would be intuitive to hypothesize that *EPO* gene polymorphisms could be involved in the susceptibility to DME. However, few studies have investigated the association of genetic variants with DME [44, 45] and Abhary et al. [11] were the only ones who reported the association of *EPO* polymorphisms with DME. In their study, the minor alleles of the rs1617640 (G), rs507392 (C), and rs551238 (C) polymorphisms were associated with the increased risk of DME in Australians with T2DM. The GCC haplotype was also associated with DME in T2DM [11]. In our case–control study, however, we did not detect such an association, which may be due to the fact that data on DME were available to only a subset of the patients. Thus, the impact of *EPO* polymorphisms on DME susceptibility is yet to be defined.

## Conclusion

Despite some limitations and the negative findings of our case–control study, suggestive evidence provided by our meta-analysis supports *EPO* as a potential gene involved in the susceptibility of DR, with the rs1617640 polymorphism deserving further investigation.

## Supplementary Information

**Additional file 1: Supplementary Table S1.** Clinical and demographic profile of Brazilian T2DM patients. **Supplementary Table S2** Genotype and allele distribution of *EPO* polymorphisms according to the period of inclusion in the study. **Supplementary Table S3** Genotype and allele distribution of *EPO* polymorphisms according to the skin color/ethnicity. **Supplementary Table S4** Haplotype frequencies of *EPO* polymorphisms according to the presence or absence of diabetic retinopathy (DR). **Supplementary Table S5** Genotype, allele, and haplotype frequencies of *EPO* polymorphisms according to the presence or absence of diabetic macular edema (DME).

**Additional file 2: Table S6.** Quality assessment of included studies in the meta-analysis according to the Newcastle–Ottawa Scale (NOS).

**Additional file 3: Table S7.** Pooled estimates for the overall association between the *EPO* rs1617640 polymorphism and DR in the sensitivity analyses.

**Additional file 4: Figure S1−S126.** Forest and funnel plots of the association between *EPO *gene polymorphisms and diabetic retinopathy.

**Additional file 5:** **Table S8.** Summary of the main differences between our meta-analysis and the previous one by Li et al. [41].

## Data Availability

The genotyping dataset generated during the case–control study are available in the Figshare repository (https://doi.org/10.6084/m9.figshare.16417161). The data used in the meta-analysis are included within the supplementary material (Additional file 4: Fig. S1-S126). Additional data are available from the corresponding author on reasonable request.

## Abbreviations

DR: Diabetic retinopathy
NPDR: Non-proliferative diabetic retinopathy
PDR: Proliferative diabetic retinopathy
EPO: Erythropoietin
ESRD: End-stage renal disease
T2DM: Type 2 diabetes mellitus
DME: Diabetic macular edema
PCR: Polymerase chain reaction
HWE: Hardy–Weinberg equilibrium
LD: Linkage disequilibrium
OR: Odds ratio
95% CI: 95% Confidence interval
NOS: Newcastle–Ottawa Scale
T1DM: Type 1 diabetes mellitus
SNP: Single nucleotide polymorphism
