# Supplementary material for: Association of polymorphisms in the erythropoietin gene with diabetic retinopathy: a case–control study and systematic review with meta-analysis
Source: BMC Ophthalmol. 2022 Jun 4;22:250. doi: 10.1186/s12886-022-02467-y (PMC9167513; doi:10.1186/s12886-022-02467-y)
Supplement: Supplementary file 1 — Additional file 1: Supplementary Table S1. Clinical and demographic profile of Brazilian T2DM patients. Supplementary Table S2 Genotype and allele distribution of EPO polymorphisms according to the period of inclusion in the study. Supplementary Table S3 Genotype and allele distribution of EPO polymorphisms according to the skin color/ethnicity. Supplementary Table S4 Haplotype frequencies of EPO polymorphisms according to the presence or absence of diabetic retinopathy (DR). Supplementary Table S5 Genotype, allele, and haplotype frequencies of EPO polymorphisms according to the presence or absence of diabetic macular edema (DME). [file 12886_2022_2467_MOESM1_ESM.docx]

**Supplementary Table S1** Clinical and demographic profile of Brazilian T2DM patients

| **Variable** | **All patients**  **(*n* = 1042)** | **Without DR**  **(*n* = 488)** | **NPDR**  **(*n* = 317)** | **PDR**  **(*n* = 237)** | ***P**** |
| --- | --- | --- | --- | --- | --- |
| Age (years) | 60.3 ± 9.5 | 59.0 ± 9.8^a^ | 61.0 ± 9.4^a,b^ | 62.1 ± 8.5^b^ | <0.001 |
| Male gender, n (%) | 487 (46.7) | 191 (39.1)^a^ | 154 (48.6)^b^ | 142 (59.9)^c^ | <0.001 |
| Non-white, n (%) | 115 (11.0) | 62 (12.7) | 30 (9.5) | 23 (9.7) | 0.271 |
| Duration of diabetes (years) | 13.5 ± 8.2 | 11.4 ± 7.3^a^ | 13.9 ± 8.2^b^ | 17.0 ± 8.8^c^ | <0.001 |
| Daily insulin use (%) | 49.0 | 36.6^a^ | 56.6^b^ | 64.3^b^ | <0.001 |
| HbA1c (%) | 7.5 ± 2.0 | 7.6 ± 2.2 | 7.6 ± 2.0 | 7.2 ± 1.7 | 0.146 |
| Body mass index (kg/m²) | 29.5 ± 5.7 | 30.5 ± 6.3^a^ | 28.9 ± 5.2^b^ | 28.2 ± 4.6^b^ | <0.001 |
| History of smoking (%) | 46.5 | 49.5 | 44.0 | 43.7 | 0.201 |
| Hypertension (%) | 76.4 | 74.3 | 78.1 | 78.7 | 0.334 |
| Systolic BP (mmHg) | 143 ± 24 | 140 ± 23^a^ | 146 ± 25^b^ | 144 ± 23^a,b^ | 0.021 |
| Diastolic BP (mmHg) | 84 ± 13 | 84 ± 14 | 85 ± 13 | 84 ± 12 | 0.218 |
| Serum creatinine (µmol/L) | 73 (55**−**102) | 66 (54**−**83)^a^ | 73 (54**−**91)^a^ | 129 (73**−**479)^b^ | <0.001 |
| eGFR (mL/min/1.73m²) | 88 (60**−**103) | 96 (76**−**105)^a^ | 89 (68**−**103)^b^ | 49 (9**−**85)^c^ | <0.001 |
| Total cholesterol (mmol/L) | 5.1 ± 1.3 | 5.0 ± 1.2 | 5.1 ± 1.5 | 5.1 ± 1.3 | 0.897 |
| HDL cholesterol (mmol/L) | 1.13 ± 0.32 | 1.14 ± 0.30 | 1.13 ± 0.33 | 1.09 ± 0.33 | 0.062 |
| LDL cholesterol (mmol/L) | 3.0 ± 1.2 | 3.0 ± 1.1 | 3.0 ± 1.1 | 3.0 ± 1.2 | 0.964 |
| Triglycerides (mmol/L) | 1.8 (1.3**−**2.7) | 1.8 (1.3**−**2.7) | 1.8 (1.2**−**2.5) | 1.8 (1.3**−**2.7) | 0.635 |
| Diabetic kidney disease (%) | 59.1 | 50.1^a^ | 57.1^a^ | 82.9^b^ | <0.001 |

Data are shown as mean ± standard deviation, median (and 25th**−**75th percentiles), absolute frequency (and percentage), or percentage. **P*-values were computed using Kruskal-Wallis for continuous variables or chi-square for categorical variables to compare the three groups of patients. The superscript letters indicate whether the means, medians, or percentages showed statistically significant differences between groups after the correction for multiple pairwise comparisons. Means, medians, or percentages indicated with the same superscript letter did not differ significantly (*P* > 0.05), while the means, medians, or percentages indicated with different superscript letters were significantly different (*P* < 0.05) following the Dunn’s test (continuous variables) or the Bonferroni correction (categorical variables). T2DM: type 2 diabetes mellitus, DR: diabetic retinopathy, NPDR: non-proliferative DR, PDR: proliferative DR, HbA1c: glycated hemoglobin; BP: blood pressure, eGFR: estimated glomerular filtration rate, HDL: high‐density lipoprotein, LDL: low‐density lipoprotein

**Supplementary Table S2** Genotype and allele distribution of *EPO* polymorphisms according to the period of inclusion in the study

| **Polymorphism** | **1999−2010** | **2015−2017** | ***P*** |
| --- | --- | --- | --- |
| rs1617640 | *n* = 731 | *n* = 302 |  |
| Genotype |  |  |  |
| TT | 316 (43.2) | 123 (40.8) | 0.361 |
| TG | 340 (46.5) | 139 (46.0) |  |
| GG | 75 (10.3) | 40 (13.2) |  |
| Allele |  |  |  |
| T | 0.66 | 0.64 | 0.253 |
| G | 0.34 | 0.36 |  |
| rs507392 | *n* = 722 | *n* = 297 |  |
| Genotype |  |  |  |
| TT | 308 (42.6) | 119 (40.1) | 0.283 |
| TC | 339 (47.0) | 137 (46.1) |  |
| CC | 75 (10.4) | 41 (13.8) |  |
| Allele |  |  |  |
| T | 0.66 | 0.63 | 0.214 |
| C | 0.34 | 0.37 |  |
| rs551238 | *n* = 731 | *n* = 297 |  |
| Genotype |  |  |  |
| AA | 311 (42.6) | 117 (39.4) | 0.428 |
| AC | 338 (46.2) | 139 (46.8) |  |
| CC | 82 (11.2) | 41 (13.8) |  |
| Allele |  |  |  |
| A | 0.66 | 0.63 | 0.236 |
| C | 0.34 | 0.37 |  |

Data are shown as absolute frequency (and percentage) or relative frequency.

**Supplementary Table S3** Genotype and allele distribution of *EPO* polymorphisms according to the skin color/ethnicity

| **Polymorphism** | **White** | **Non-white** | ***P*** |
| --- | --- | --- | --- |
| rs1617640 | *n* = 918 | *n* = 115 |  |
| Genotype |  |  |  |
| TT | 389 (42.4) | 49 (42.6) | 0.996 |
| TG | 427 (46.5) | 53 (46.1) |  |
| GG | 102 (11.1) | 13 (11.3) |  |
| Allele |  |  |  |
| T | 0.66 | 0.66 | >0.999 |
| G | 0.34 | 0.34 |  |
| rs507392 | *n* = 908 | *n* = 111 |  |
| Genotype |  |  |  |
| TT | 380 (41.9) | 46 (41.4) | 0.910 |
| TC | 426 (46.9) | 51 (46.0) |  |
| CC | 102 (11.2) | 14 (12.6) |  |
| Allele |  |  |  |
| T | 0.65 | 0.64 | 0.850 |
| C | 0.35 | 0.36 |  |
| rs551238 | *n* = 921 | *n* = 107 |  |
| Genotype |  |  |  |
| AA | 383 (41.6) | 44 (41.1) | 0.996 |
| AC | 427 (46.3) | 50 (46.8) |  |
| CC | 111 (12.1) | 13 (12.1) |  |
| Allele |  |  |  |
| A | 0.65 | 0.64 | 0.995 |
| C | 0.35 | 0.36 |  |

Data are shown as absolute frequency (and percentage) or relative frequency.

**Supplementary Table S4** Haplotype frequencies of *EPO* polymorphisms according to the presence or absence of diabetic retinopathy (DR)

| **Haplotype** | **All patients**  **(*n* = 2056)** | **Without DR**  **(*n* = 956)** | **NPDR**  **(*n* = 630)** | **PDR**  **(*n* = 470)** | ***P**** |
| --- | --- | --- | --- | --- | --- |
| TTA | 0.643 | 0.645 | 0.662 | 0.614 | 0.284 |
| GCC | 0.331 | 0.330 | 0.312 | 0.360 |  |
| Others | 0.026 | 0.025 | 0.026 | 0.026 |  |

Data are shown as relative frequency and are based on the total number of chromosomes.

**P*-value was estimated by a permutation test implemented in the PHASE software. T2DM: type 2 diabetes mellitus, NPDR: non-proliferative DR, PDR: proliferative DR

**Supplementary Table S5** Genotype, allele, and haplotype frequencies of *EPO* polymorphisms according to the presence or absence of diabetic macular edema (DME)

| **Polymorphism** |  | **Without DME**  **(*n* = 129)** | **With DME**  **(*n* = 10)** | ***P**** |
| --- | --- | --- | --- | --- |
| rs1617640 |  |  |  |  |
| Genotype | TT | 61 (47.2) | 4 (40.0) | 0.242 |
|  | TG | 54 (41.9) | 6 (60.0) |  |
|  | GG | 14 (10.9) | 0 (0.0) |  |
| Allele | T | 0.68 | 0.70 | >0.999 |
|  | G | 0.32 | 0.30 |  |
| rs507392 |  |  |  |  |
| Genotype | TT | 60 (46.5) | 4 (40.0) | 0.252 |
|  | TC | 55 (42.6) | 6 (60.0) |  |
|  | CC | 14 (10.9) | 0 (0.0) |  |
| Allele | T | 0.68 | 0.70 | >0.999 |
|  | C | 0.32 | 0.30 |  |
| rs551238 |  |  |  |  |
| Genotype | AA | 59 (45.7) | 4 (40.0) | 0.262 |
|  | AC | 56 (43.4) | 6 (60.0) |  |
|  | CC | 14 (10.9) | 0 (0.0) |  |
| Allele | A | 0.67 | 0.70 |  |
|  | C | 0.33 | 0.30 |  |
| Haplotype | TTA | 0.674 | 0.700 | 0.591 |
|  | GCC | 0.306 | 0.300 |  |
|  | Others | 0.020 | 0.000 |  |

Data are shown as absolute frequency (and percentage) or relative frequency.

**P*-values were estimated by chi-square test for the comparison of genotype and allele frequencies and by a permutation test for the comparison of haplotype frequencies.
