# Supplementary material for: Association of polymorphisms in the erythropoietin gene with diabetic retinopathy: a case–control study and systematic review with meta-analysis
Source: BMC Ophthalmol. 2022 Jun 4;22:250. doi: 10.1186/s12886-022-02467-y (PMC9167513; doi:10.1186/s12886-022-02467-y)
Supplement: Supplementary file 3 — Additional file 3: Table S7. Pooled estimates for the overall association between the EPO rs1617640 polymorphism and DR in the sensitivity analyses. [file 12886_2022_2467_MOESM3_ESM.docx]

**Supplementary Table S7** Pooled estimates for the overall association between the *EPO* rs1617640 polymorphism and DR in the sensitivity analyses

| **Genetic model and subgroup** | **Number*** | ***n* (cases/controls)** | **Heterogeneity** | | | ***P* for funnel plot asymmetry** | **Effect model** | **Pooled OR (95% CI)** |
| --- | --- | --- | --- | --- | --- | --- | --- | --- |
|  |  |  | **I² (%)** | **τ²** | ***P***** |  |  |  |
| Dominant (GG+TG vs. TT) |  |  |  |  |  |  |  |  |
| All | 14 | 9117 (4462/4655) | 68 | 0.07 | 0.001 | 0.693 | Random | 0.87 (0.74−1.03) |
| Without Tong et al. (2) | 13 | 7678 (3597/4081) | 48 | 0.03 | 0.026 | 0.577 | Fixed | 0.94 (0.85−1.04) |
| Only in HWE | 11 | 6977 (3593/3384) | 62 | 0.06 | 0.003 | 0.379 | Random | **0.82 (0.68−0.98)** |
| HWE and without Tong et al. (2) | 10 | 5538 (2728/2810) | 38 | 0.03 | 0.103 | 0.973 | Fixed | **0.87 (0.77−0.98)** |
| Recessive (GG vs. TG+TT) |  |  |  |  |  |  |  |  |
| All | 14 | 9117 (4462/4655) | 71 | 0.15 | <0.001 | 0.517 | Random | 0.97 (0.76−1.25) |
| Without Tong et al. (2) | 13 | 7678 (3597/4081) | 63 | 0.11 | 0.001 | 0.977 | Random | 1.03 (0.81−1.31) |
| Only in HWE | 11 | 6977 (3593/3384) | 55 | 0.07 | 0.013 | 0.364 | Random | 0.88 (0.71−1.10) |
| HWE and without Tong et al. (2) | 10 | 5538 (2728/2810) | 20 | 0.02 | 0.257 | 0.921 | Fixed | 0.98 (0.83−1.14) |
| Homozygous additive (GG vs. TT) |  |  |  |  |  |  |  |  |
| All | 14 | 5111 (2526/2585) | 76 | 0.25 | <0.001 | 0.563 | Random | 0.89 (0.66−1.22) |
| Without Tong et al. (2) | 13 | 4398 (2080/2318) | 65 | 0.17 | 0.001 | 0.950 | Random | 0.96 (0.73−1.28) |
| Only in HWE | 11 | 3845 (2025/1820) | 65 | 0.15 | 0.002 | 0.468 | Random | 0.78 (0.58−1.04) |
| HWE and without Tong et al. (2) | 10 | 3132 (1579/1553) | 34 | 0.05 | 0.139 | 0.873 | Fixed | 0.87 (0.72−1.05) |
| Heterozygous additive (TG vs. TT) |  |  |  |  |  |  |  |  |
| All | 14 | 7781 (3819/3962) | 57 | 0.05 | 0.005 | 0.911 | Random | 0.87 (0.75−1.02) |
| Without Tong et al. (2) | 13 | 6572 (3065/3507) | 38 | 0.02 | 0.078 | 0.387 | Fixed | 0.93 (0.84−1.04) |
| Only in HWE | 11 | 5855 (3053/2802) | 50 | 0.04 | 0.030 | 0.473 | Random | **0.82 (0.69−0.97)** |
| HWE and without Tong et al. (2) | 10 | 4646 (2299/2347) | 33 | 0.02 | 0.146 | 0.970 | Fixed | **0.86 (0.76−0.98)** |
| Overdominant (TG vs. GG+TT) |  |  |  |  |  |  |  |  |
| All | 14 | 9117 (4462/4655) | 31 | 0.01 | 0.125 | 0.742 | Fixed | 0.92 (0.84−1.00) |
| Without Tong et al. (2) | 13 | 7678 (3597/4081) | 31 | 0.01 | 0.132 | 0.425 | Fixed | 0.94 (0.86−1.04) |
| Only in HWE | 11 | 6977 (3593/3384) | 7 | <0.01 | 0.375 | 0.689 | Fixed | **0.88 (0.79−0.97)** |
| HWE and without Tong et al. (2) | 10 | 5538 (2728/2810) | 12 | <0.01 | 0.330 | 0.947 | Fixed | 0.89 (0.80−1.00) |
| Allele contrast (G vs. T) |  |  |  |  |  |  |  |  |
| All | 14 | 18234 (8924/9310) | 75 | 0.05 | <0.001 | 0.594 | Random | 0.93 (0.81−1.06) |
| Without Tong et al. (2) | 13 | 15356 (7194/8162) | 60 | 0.03 | 0.003 | 0.766 | Random | 0.96 (0.86−1.08) |
| Only in HWE | 11 | 13954 (7186/6768) | 67 | 0.03 | <0.001 | 0.380 | Random | 0.88 (0.77−1.01) |
| HWE and without Tong et al. (2) | 10 | 11076 (5456/5620) | 39 | 0.01 | 0.098 | 0.946 | Fixed | 0.93 (0.85−1.01) |

*Number of independent sets of cases and controls. **Computed by Q-test. Statistically suggestive association estimates are shown in bold, considering the most appropriate model for each analysis (fixed- or random-effects). DR: diabetic retinopathy, HWE: Hardy-Weinberg equilibrium, OR: odds ratio, 95% CI: 95% confidence interval
