# Supplementary material for: Association of polymorphisms in the erythropoietin gene with diabetic retinopathy: a case–control study and systematic review with meta-analysis
Source: BMC Ophthalmol. 2022 Jun 4;22:250. doi: 10.1186/s12886-022-02467-y (PMC9167513; doi:10.1186/s12886-022-02467-y)
Supplement: Supplementary file 4 — Additional file 4: Figure S1−S126. Forest and funnel plots of the association between EPO gene polymorphisms and diabetic retinopathy. [file 12886_2022_2467_MOESM4_ESM.docx]

**Supplementary Material**

**Supplementary Figures S1**−**S126.** Forest and funnel plots of the association between *EPO* gene polymorphisms and diabetic retinopathy.

**Supplementary Figures**

| **Figure S1** | Forest plot of the association between the *EPO* rs1617640 polymorphism and diabetic retinopathy in the overall group analysis, under the dominant genetic model for the minor allele (GG+TG vs. TT) | **20** |
| --- | --- | --- |
| **Figure S2** | Funnel plot of the association between the *EPO* rs1617640 polymorphism and diabetic retinopathy in the overall group analysis, under the dominant genetic model for the minor allele (GG+TG vs. TT) | **21** |
| **Figure S3** | Forest plot of the association between the *EPO* rs1617640 polymorphism and diabetic retinopathy in the overall group analysis without the cohort #2 by Tong et al. (2008), under the dominant genetic model for the minor allele (GG+TG vs. TT) | **22** |
| **Figure S4** | Funnel plot of the association between the *EPO* rs1617640 polymorphism and diabetic retinopathy in the overall group analysis without the cohort #2 by Tong et al. (2008), under the dominant genetic model for the minor allele (GG+TG vs. TT) | **23** |
| **Figure S5** | Forest plot of the association between the *EPO* rs1617640 polymorphism and diabetic retinopathy in the overall group analysis including only the sets with controls in Hardy-Weinberg equilibrium, under the dominant genetic model for the minor allele (GG+TG vs. TT) | **24** |
| **Figure S6** | Funnel plot of the association between the *EPO* rs1617640 polymorphism and diabetic retinopathy in the overall group analysis including only the sets with controls in Hardy-Weinberg equilibrium, under the dominant genetic model for the minor allele (GG+TG vs. TT) | **25** |
| **Figure S7** | Forest plot of the association between the *EPO* rs1617640 polymorphism and diabetic retinopathy in the overall group analysis, including only the sets with controls in Hardy-Weinberg equilibrium and excluding the cohort #2 by Tong et al. (2008), under the dominant genetic model for the minor allele (GG+TG vs. TT) | **26** |
| **Figure S8** | Funnel plot of the association between the *EPO* rs1617640 polymorphism and diabetic retinopathy in the overall group analysis, including only the sets with controls in Hardy-Weinberg equilibrium and excluding the cohort #2 by Tong et al. (2008), under the dominant genetic model for the minor allele (GG+TG vs. TT) | **27** |
| **Figure S9** | Forest plot of the association between the *EPO* rs1617640 polymorphism and diabetic retinopathy in the overall group analysis, under the recessive genetic model for the minor allele (GG vs. TG+TT) | **28** |
| **Figure S10** | Funnel plot of the association between the *EPO* rs1617640 polymorphism and diabetic retinopathy in the overall group analysis, under the recessive genetic model for the minor allele (GG vs. TG+TT) | **29** |
| **Figure S11** | Forest plot of the association between the *EPO* rs1617640 polymorphism and diabetic retinopathy in the overall group analysis without the cohort #2 by Tong et al. (2008), under the recessive genetic model for the minor allele (GG vs. TG+TT) | **30** |
| **Figure S12** | Funnel plot of the association between the *EPO* rs1617640 polymorphism and diabetic retinopathy in the overall group analysis without the cohort #2 by Tong et al. (2008), under the recessive genetic model for the minor allele (GG vs. TG+TT) | **31** |
| **Figure S13** | Forest plot of the association between the *EPO* rs1617640 polymorphism and diabetic retinopathy in the overall group analysis including only the sets with controls in Hardy-Weinberg equilibrium, under the recessive genetic model for the minor allele (GG vs. TG+TT) | **32** |
| **Figure S14** | Funnel plot of the association between the *EPO* rs1617640 polymorphism and diabetic retinopathy in the overall group analysis including only the sets with controls in Hardy-Weinberg equilibrium, under the recessive genetic model for the minor allele (GG vs. TG+TT) | **33** |
| **Figure S15** | Forest plot of the association between the *EPO* rs1617640 polymorphism and diabetic retinopathy in the overall group analysis, including only the sets with controls in Hardy-Weinberg equilibrium and excluding the cohort #2 by Tong et al. (2008), under the recessive genetic model for the minor allele (GG vs. TG+TT) | **34** |
| **Figure S16** | Funnel plot of the association between the *EPO* rs1617640 polymorphism and diabetic retinopathy in the overall group analysis, including only the sets with controls in Hardy-Weinberg equilibrium and excluding the cohort #2 by Tong et al. (2008), under the recessive genetic model for the minor allele (GG vs. TG+TT) | **35** |
| **Figure S17** | Forest plot of the association between the *EPO* rs1617640 polymorphism and diabetic retinopathy in the overall group analysis, under the homozygous additive genetic model for the minor allele (GG vs. TT) | **36** |
| **Figure S18** | Funnel plot of the association between the *EPO* rs1617640 polymorphism and diabetic retinopathy in the overall group analysis, under the homozygous additive genetic model for the minor allele (GG vs. TT) | **37** |
| **Figure S19** | Forest plot of the association between the *EPO* rs1617640 polymorphism and diabetic retinopathy in the overall group analysis without the cohort #2 by Tong et al. (2008), under the homozygous additive genetic model for the minor allele (GG vs. TT) | **38** |
| **Figure S20** | Funnel plot of the association between the *EPO* rs1617640 polymorphism and diabetic retinopathy in the overall group analysis without the cohort #2 by Tong et al. (2008), under the homozygous additive genetic model for the minor allele (GG vs. TT) | **39** |
| **Figure S21** | Forest plot of the association between the *EPO* rs1617640 polymorphism and diabetic retinopathy in the overall group analysis including only the sets with controls in Hardy-Weinberg equilibrium, under the homozygous additive genetic model for the minor allele (GG vs. TT) | **40** |
| **Figure S22** | Funnel plot of the association between the *EPO* rs1617640 polymorphism and diabetic retinopathy in the overall group analysis including only the sets with controls in Hardy-Weinberg equilibrium, under the homozygous additive genetic model for the minor allele (GG vs. TT) | **41** |
| **Figure S23** | Forest plot of the association between the *EPO* rs1617640 polymorphism and diabetic retinopathy in the overall group analysis, including only the sets with controls in Hardy-Weinberg equilibrium and excluding the cohort #2 by Tong et al. (2008), under the homozygous additive genetic model for the minor allele (GG vs. TT) | **42** |
| **Figure S24** | Funnel plot of the association between the *EPO* rs1617640 polymorphism and diabetic retinopathy in the overall group analysis, including only the sets with controls in Hardy-Weinberg equilibrium and excluding the cohort #2 by Tong et al. (2008), under the homozygous additive genetic model for the minor allele (GG vs. TT) | **43** |
| **Figure S25** | Forest plot of the association between the *EPO* rs1617640 polymorphism and diabetic retinopathy in the overall group analysis, under the heterozygous additive genetic model for the minor allele (TG vs. TT) | **44** |
| **Figure S26** | Funnel plot of the association between the *EPO* rs1617640 polymorphism and diabetic retinopathy in the overall group analysis, under the heterozygous additive genetic model for the minor allele (TG vs. TT) | **45** |
| **Figure S27** | Forest plot of the association between the *EPO* rs1617640 polymorphism and diabetic retinopathy in the overall group analysis without the cohort #2 by Tong et al. (2008), under the heterozygous additive genetic model for the minor allele (TG vs. TT) | **46** |
| **Figure S28** | Funnel plot of the association between the *EPO* rs1617640 polymorphism and diabetic retinopathy in the overall group analysis without the cohort #2 by Tong et al. (2008), under the heterozygous additive genetic model for the minor allele (TG vs. TT) | **47** |
| **Figure S29** | Forest plot of the association between the *EPO* rs1617640 polymorphism and diabetic retinopathy in the overall group analysis including only the sets with controls in Hardy-Weinberg equilibrium, under the heterozygous additive genetic model for the minor allele (TG vs. TT) | **48** |
| **Figure S30** | Funnel plot of the association between the *EPO* rs1617640 polymorphism and diabetic retinopathy in the overall group analysis including only the sets with controls in Hardy-Weinberg equilibrium, under the heterozygous additive genetic model for the minor allele (TG vs. TT) | **49** |
| **Figure S31** | Forest plot of the association between the *EPO* rs1617640 polymorphism and diabetic retinopathy in the overall group analysis, including only the sets with controls in Hardy-Weinberg equilibrium and excluding the cohort #2 by Tong et al. (2008), under the heterozygous additive genetic model for the minor allele (TG vs. TT) | **50** |
| **Figure S32** | Funnel plot of the association between the *EPO* rs1617640 polymorphism and diabetic retinopathy in the overall group analysis, including only the sets with controls in Hardy-Weinberg equilibrium and excluding the cohort #2 by Tong et al. (2008), under the heterozygous additive genetic model for the minor allele (TG vs. TT) | **51** |
| **Figure S33** | Forest plot of the association between the *EPO* rs1617640 polymorphism and diabetic retinopathy in the overall group analysis, under the overdominant genetic model (TG vs. GG+TT) | **52** |
| **Figure S34** | Funnel plot of the association between the *EPO* rs1617640 polymorphism and diabetic retinopathy in the overall group analysis, under the overdominant genetic model (TG vs. GG+TT) | **53** |
| **Figure S35** | Forest plot of the association between the *EPO* rs1617640 polymorphism and diabetic retinopathy in the overall group analysis without the cohort #2 by Tong et al. (2008), under the overdominant genetic model (TG vs. GG+TT) | **54** |
| **Figure S36** | Funnel plot of the association between the *EPO* rs1617640 polymorphism and diabetic retinopathy in the overall group analysis without the cohort #2 by Tong et al. (2008), under the overdominant genetic model (TG vs. GG+TT) | **55** |
| **Figure S37** | Forest plot of the association between the *EPO* rs1617640 polymorphism and diabetic retinopathy in the overall group analysis including only the sets with controls in Hardy-Weinberg equilibrium, under the overdominant genetic model (TG vs. GG+TT) | **56** |
| **Figure S38** | Funnel plot of the association between the *EPO* rs1617640 polymorphism and diabetic retinopathy in the overall group analysis including only the sets with controls in Hardy-Weinberg equilibrium, under the overdominant genetic model (TG vs. GG+TT) | **57** |
| **Figure S39** | Forest plot of the association between the *EPO* rs1617640 polymorphism and diabetic retinopathy in the overall group analysis, including only the sets with controls in Hardy-Weinberg equilibrium and excluding the cohort #2 by Tong et al. (2008), under the overdominant genetic model (TG vs. GG+TT) | **58** |
| **Figure S40** | Funnel plot of the association between the *EPO* rs1617640 polymorphism and diabetic retinopathy in the overall group analysis, including only the sets with controls in Hardy-Weinberg equilibrium and excluding the cohort #2 by Tong et al. (2008), under the overdominant genetic model (TG vs. GG+TT) | **59** |
| **Figure S41** | Forest plot of the association between the *EPO* rs1617640 polymorphism and diabetic retinopathy in the overall group analysis, under the allele contrast genetic model (G vs. T) | **60** |
| **Figure S42** | Funnel plot of the association between the *EPO* rs1617640 polymorphism and diabetic retinopathy in the overall group analysis, under the allele contrast genetic model (G vs. T) | **61** |
| **Figure S43** | Forest plot of the association between the *EPO* rs1617640 polymorphism and diabetic retinopathy in the overall group analysis without the cohort #2 by Tong et al. (2008), under the allele contrast genetic model (G vs. T) | **62** |
| **Figure S44** | Funnel plot of the association between the *EPO* rs1617640 polymorphism and diabetic retinopathy in the overall group analysis without the cohort #2 by Tong et al. (2008), under the allele contrast genetic model (G vs. T) | **63** |
| **Figure S45** | Forest plot of the association between the *EPO* rs1617640 polymorphism and diabetic retinopathy in the overall group analysis including only the sets with controls in Hardy-Weinberg equilibrium, under the allele contrast genetic model (G vs. T) | **64** |
| **Figure S46** | Funnel plot of the association between the *EPO* rs1617640 polymorphism and diabetic retinopathy in the overall group analysis including only the sets with controls in Hardy-Weinberg equilibrium, under the allele contrast genetic model (G vs. T) | **65** |
| **Figure S47** | Forest plot of the association between the *EPO* rs1617640 polymorphism and diabetic retinopathy in the overall group analysis, including only the sets with controls in Hardy-Weinberg equilibrium and excluding the cohort #2 by Tong et al. (2008), under the allele contrast genetic model (G vs. T) | **66** |
| **Figure S48** | Funnel plot of the association between the *EPO* rs1617640 polymorphism and diabetic retinopathy in the overall group analysis, including only the sets with controls in Hardy-Weinberg equilibrium and excluding the cohort #2 by Tong et al. (2008), under the allele contrast genetic model (G vs. T) | **67** |
| **Figure S49** | Forest plot of the association between the *EPO* rs1617640 polymorphism and proliferative diabetic retinopathy, including only the sets with controls in Hardy-Weinberg equilibrium, under the dominant genetic model for the minor allele (GG+TG vs. TT) | **68** |
| **Figure S50** | Forest plot of the association between the *EPO* rs1617640 polymorphism and proliferative diabetic retinopathy, including only the sets with controls in Hardy-Weinberg equilibrium, under the recessive genetic model for the minor allele (GG vs. TG+TT) | **69** |
| **Figure S51** | Forest plot of the association between the *EPO* rs1617640 polymorphism and proliferative diabetic retinopathy, including only the sets with controls in Hardy-Weinberg equilibrium, under the homozygous additive genetic model for the minor allele (GG vs. TT) | **70** |
| **Figure S52** | Forest plot of the association between the *EPO* rs1617640 polymorphism and proliferative diabetic retinopathy, including only the sets with controls in Hardy-Weinberg equilibrium, under the heterozygous additive genetic model for the minor allele (TG vs. TT) | **71** |
| **Figure S53** | Forest plot of the association between the *EPO* rs1617640 polymorphism and proliferative diabetic retinopathy, including only the sets with controls in Hardy-Weinberg equilibrium, under the overdominant genetic model (TG vs. GG+TT) | **72** |
| **Figure S54** | Forest plot of the association between the *EPO* rs1617640 polymorphism and proliferative diabetic retinopathy, including only the sets with controls in Hardy-Weinberg equilibrium, under the allele contrast genetic model (G vs. T) | **73** |
| **Figure S55** | Forest plot of the association between the *EPO* rs1617640 polymorphism and non-proliferative diabetic retinopathy, including only the sets with controls in Hardy-Weinberg equilibrium, under the dominant genetic model for the minor allele (GG+TG vs. TT) | **74** |
| **Figure S56** | Forest plot of the association between the *EPO* rs1617640 polymorphism and non-proliferative diabetic retinopathy, including only the sets with controls in Hardy-Weinberg equilibrium, under the recessive genetic model for the minor allele (GG vs. TG+TT) | **75** |
| **Figure S57** | Forest plot of the association between the *EPO* rs1617640 polymorphism and non-proliferative diabetic retinopathy, including only the sets with controls in Hardy-Weinberg equilibrium, under the homozygous additive genetic model for the minor allele (GG vs. TT) | **76** |
| **Figure S58** | Forest plot of the association between the *EPO* rs1617640 polymorphism and non-proliferative diabetic retinopathy, including only the sets with controls in Hardy-Weinberg equilibrium, under the heterozygous additive genetic model for the minor allele (TG vs. TT) | **77** |
| **Figure S59** | Forest plot of the association between the *EPO* rs1617640 polymorphism and non-proliferative diabetic retinopathy, including only the sets with controls in Hardy-Weinberg equilibrium, under the overdominant genetic model (TG vs. GG+TT) | **78** |
| **Figure S60** | Forest plot of the association between the *EPO* rs1617640 polymorphism and non-proliferative diabetic retinopathy, including only the sets with controls in Hardy-Weinberg equilibrium, under the allele contrast genetic model (G vs. T) | **79** |
| **Figure S61** | Forest plot of the association between the *EPO* rs1617640 polymorphism and diabetic retinopathy in subjects with type 2 diabetes, including only the sets with controls in Hardy-Weinberg equilibrium, under the dominant genetic model for the minor allele (GG+TG vs. TT) | **80** |
| **Figure S62** | Forest plot of the association between the *EPO* rs1617640 polymorphism and diabetic retinopathy in subjects with type 2 diabetes, including only the sets with controls in Hardy-Weinberg equilibrium, under the recessive genetic model for the minor allele (GG vs. TG+TT) | **81** |
| **Figure S63** | Forest plot of the association between the *EPO* rs1617640 polymorphism and diabetic retinopathy in subjects with type 2 diabetes, including only the sets with controls in Hardy-Weinberg equilibrium, under the homozygous additive genetic model for the minor allele (GG vs. TT) | **82** |
| **Figure S64** | Forest plot of the association between the *EPO* rs1617640 polymorphism and diabetic retinopathy in subjects with type 2 diabetes, including only the sets with controls in Hardy-Weinberg equilibrium, under the heterozygous additive genetic model for the minor allele (TG vs. TT) | **83** |
| **Figure S65** | Forest plot of the association between the *EPO* rs1617640 polymorphism and diabetic retinopathy in subjects with type 2 diabetes, including only the sets with controls in Hardy-Weinberg equilibrium, under the overdominant genetic model (TG vs. GG+TT) | **84** |
| **Figure S66** | Forest plot of the association between the *EPO* rs1617640 polymorphism and diabetic retinopathy in subjects with type 2 diabetes, including only the sets with controls in Hardy-Weinberg equilibrium, under the allele contrast genetic model (G vs. T) | **85** |
| **Figure S67** | Forest plot of the association between the *EPO* rs1617640 polymorphism and diabetic retinopathy in subjects with type 1 diabetes, including only the sets with controls in Hardy-Weinberg equilibrium, under the dominant genetic model for the minor allele (GG+TG vs. TT) | **86** |
| **Figure S68** | Forest plot of the association between the *EPO* rs1617640 polymorphism and diabetic retinopathy in subjects with type 1 diabetes, including only the sets with controls in Hardy-Weinberg equilibrium, under the recessive genetic model for the minor allele (GG vs. TG+TT) | **87** |
| **Figure S69** | Forest plot of the association between the *EPO* rs1617640 polymorphism and diabetic retinopathy in subjects with type 1 diabetes, including only the sets with controls in Hardy-Weinberg equilibrium, under the homozygous additive genetic model for the minor allele (GG vs. TT) | **88** |
| **Figure S70** | Forest plot of the association between the *EPO* rs1617640 polymorphism and diabetic retinopathy in subjects with type 1 diabetes, including only the sets with controls in Hardy-Weinberg equilibrium, under the heterozygous additive genetic model for the minor allele (TG vs. TT) | **89** |
| **Figure S71** | Forest plot of the association between the *EPO* rs1617640 polymorphism and diabetic retinopathy in subjects with type 1 diabetes, including only the sets with controls in Hardy-Weinberg equilibrium, under the overdominant genetic model (TG vs. GG+TT) | **90** |
| **Figure S72** | Forest plot of the association between the *EPO* rs1617640 polymorphism and diabetic retinopathy in subjects with type 1 diabetes, including only the sets with controls in Hardy-Weinberg equilibrium, under the allele contrast genetic model (G vs. T) | **91** |
| **Figure S73** | Forest plot of the association between the *EPO* rs1617640 polymorphism and diabetic retinopathy in non-Asians, including only the sets with controls in Hardy-Weinberg equilibrium, under the dominant genetic model for the minor allele (GG+TG vs. TT) | **92** |
| **Figure S74** | Forest plot of the association between the *EPO* rs1617640 polymorphism and diabetic retinopathy in non-Asians, including only the sets with controls in Hardy-Weinberg equilibrium, under the recessive genetic model for the minor allele (GG vs. TG+TT) | **93** |
| **Figure S75** | Forest plot of the association between the *EPO* rs1617640 polymorphism and diabetic retinopathy in non-Asians, including only the sets with controls in Hardy-Weinberg equilibrium, under the homozygous additive genetic model for the minor allele (GG vs. TT) | **94** |
| **Figure S76** | Forest plot of the association between the *EPO* rs1617640 polymorphism and diabetic retinopathy in non-Asians, including only the sets with controls in Hardy-Weinberg equilibrium, under the heterozygous additive genetic model for the minor allele (TG vs. TT) | **95** |
| **Figure S77** | Forest plot of the association between the *EPO* rs1617640 polymorphism and diabetic retinopathy in non-Asians, including only the sets with controls in Hardy-Weinberg equilibrium, under the overdominant genetic model (TG vs. GG+TT) | **96** |
| **Figure S78** | Forest plot of the association between the *EPO* rs1617640 polymorphism and diabetic retinopathy in non-Asians, including only the sets with controls in Hardy-Weinberg equilibrium, under the allele contrast genetic model (G vs. T) | **97** |
| **Figure S79** | Forest plot of the association between the *EPO* rs1617640 polymorphism and diabetic retinopathy in Asians, including only the sets with controls in Hardy-Weinberg equilibrium, under the dominant genetic model for the minor allele (GG+TG vs. TT) | **98** |
| **Figure S80** | Forest plot of the association between the *EPO* rs1617640 polymorphism and diabetic retinopathy in Asians, including only the sets with controls in Hardy-Weinberg equilibrium, under the recessive genetic model for the minor allele (GG vs. TG+TT) | **99** |
| **Figure S81** | Forest plot of the association between the *EPO* rs1617640 polymorphism and diabetic retinopathy in Asians, including only the sets with controls in Hardy-Weinberg equilibrium, under the homozygous additive genetic model for the minor allele (GG vs. TT) | **100** |
| **Figure S82** | Forest plot of the association between the *EPO* rs1617640 polymorphism and diabetic retinopathy in Asians, including only the sets with controls in Hardy-Weinberg equilibrium, under the heterozygous additive genetic model for the minor allele (TG vs. TT) | **101** |
| **Figure S83** | Forest plot of the association between the *EPO* rs1617640 polymorphism and diabetic retinopathy in Asians, including only the sets with controls in Hardy-Weinberg equilibrium, under the overdominant genetic model (TG vs. GG+TT) | **102** |
| **Figure S84** | Forest plot of the association between the *EPO* rs1617640 polymorphism and diabetic retinopathy in Asians, including only the sets with controls in Hardy-Weinberg equilibrium, under the allele contrast genetic model (G vs. T) | **103** |
| **Figure S85** | Forest plot of the association between the *EPO* rs507392 polymorphism and diabetic retinopathy in the overall group analysis, under the dominant genetic model for the minor allele (CC+TC vs. TT) | **104** |
| **Figure S86** | Forest plot of the association between the *EPO* rs507392 polymorphism and diabetic retinopathy in the overall group analysis, under the recessive genetic model for the minor allele (CC vs. TC+TT) | **105** |
| **Figure S87** | Forest plot of the association between the *EPO* rs507392 polymorphism and diabetic retinopathy in the overall group analysis, under the homozygous additive genetic model for the minor allele (CC vs. TT) | **106** |
| **Figure S88** | Forest plot of the association between the *EPO* rs507392 polymorphism and diabetic retinopathy in the overall group analysis, under the heterozygous additive genetic model for the minor allele (TC vs. TT) | **107** |
| **Figure S89** | Forest plot of the association between the *EPO* rs507392 polymorphism and diabetic retinopathy in the overall group analysis, under the overdominant genetic model (TC vs. CC+TT) | **108** |
| **Figure S90** | Forest plot of the association between the *EPO* rs507392 polymorphism and diabetic retinopathy in the overall group analysis, under the allele contrast genetic model (C vs. T) | **109** |
| **Figure S91** | Forest plot of the association between the *EPO* rs507392 polymorphism and diabetic retinopathy in the overall group analysis including only the sets with controls in Hardy-Weinberg equilibrium, under the dominant genetic model for the minor allele (CC+TC vs. TT) | **110** |
| **Figure S92** | Forest plot of the association between the *EPO* rs507392 polymorphism and diabetic retinopathy in the overall group analysis including only the sets with controls in Hardy-Weinberg equilibrium, under the recessive genetic model for the minor allele (CC vs. TC+TT) | **111** |
| **Figure S93** | Forest plot of the association between the *EPO* rs507392 polymorphism and diabetic retinopathy in the overall group analysis including only the sets with controls in Hardy-Weinberg equilibrium, under the homozygous additive genetic model for the minor allele (CC vs. TT) | **112** |
| **Figure S94** | Forest plot of the association between the *EPO* rs507392 polymorphism and diabetic retinopathy in the overall group analysis including only the sets with controls in Hardy-Weinberg equilibrium, under the heterozygous additive genetic model for the minor allele (TC vs. TT) | **113** |
| **Figure S95** | Forest plot of the association between the *EPO* rs507392 polymorphism and diabetic retinopathy in the overall group analysis including only the sets with controls in Hardy-Weinberg equilibrium, under the overdominant genetic model (TC vs. CC+TT) | **114** |
| **Figure S96** | Forest plot of the association between the *EPO* rs507392 polymorphism and diabetic retinopathy in the overall group analysis including only the sets with controls in Hardy-Weinberg equilibrium, under the allele contrast genetic model (C vs. T) | **115** |
| **Figure S97** | Forest plot of the association between the *EPO* rs507392 polymorphism and diabetic retinopathy in subjects with type 2 diabetes, including only the sets with controls in Hardy-Weinberg equilibrium, under the dominant genetic model for the minor allele (CC+TC vs. TT) | **116** |
| **Figure S98** | Forest plot of the association between the *EPO* rs507392 polymorphism and diabetic retinopathy in subjects with type 2 diabetes, including only the sets with controls in Hardy-Weinberg equilibrium, under the recessive genetic model for the minor allele (CC vs. TC+TT) | **117** |
| **Figure S99** | Forest plot of the association between the *EPO* rs507392 polymorphism and diabetic retinopathy in subjects with type 2 diabetes, including only the sets with controls in Hardy-Weinberg equilibrium, under the homozygous additive genetic model for the minor allele (CC vs. TT) | **118** |
| **Figure S100** | Forest plot of the association between the *EPO* rs507392 polymorphism and diabetic retinopathy in subjects with type 2 diabetes, including only the sets with controls in Hardy-Weinberg equilibrium, under the heterozygous additive genetic model for the minor allele (TC vs. TT) | **119** |
| **Figure S101** | Forest plot of the association between the *EPO* rs507392 polymorphism and diabetic retinopathy in subjects with type 2 diabetes, including only the sets with controls in Hardy-Weinberg equilibrium, under the overdominant genetic model (TC vs. CC+TT) | **120** |
| **Figure S102** | Forest plot of the association between the *EPO* rs507392 polymorphism and diabetic retinopathy in subjects with type 2 diabetes, including only the sets with controls in Hardy-Weinberg equilibrium, under the allele contrast genetic model (C vs. T) | **121** |
| **Figure S103** | Forest plot of the association between the *EPO* rs551238 polymorphism and diabetic retinopathy in the overall group analysis, under the dominant genetic model for the minor allele (CC+AC vs. AA) | **122** |
| **Figure S104** | Forest plot of the association between the *EPO* rs551238 polymorphism and diabetic retinopathy in the overall group analysis, under the recessive genetic model for the minor allele (CC vs. AC+AA) | **123** |
| **Figure S105** | Forest plot of the association between the *EPO* rs551238 polymorphism and diabetic retinopathy in the overall group analysis, under the homozygous additive genetic model for the minor allele (CC vs. AA) | **124** |
| **Figure S106** | Forest plot of the association between the *EPO* rs551238 polymorphism and diabetic retinopathy in the overall group analysis, under the heterozygous additive genetic model for the minor allele (AC vs. AA) | **125** |
| **Figure S107** | Forest plot of the association between the *EPO* rs551238 polymorphism and diabetic retinopathy in the overall group analysis, under the overdominant genetic model (AC vs. CC+AA) | **126** |
| **Figure S108** | Forest plot of the association between the *EPO* rs551238 polymorphism and diabetic retinopathy in the overall group analysis, under the allele contrast genetic model (C vs. A) | **127** |
| **Figure S109** | Forest plot of the association between the *EPO* rs551238 polymorphism and diabetic retinopathy in the overall group analysis including only the sets with controls in Hardy-Weinberg equilibrium, under the dominant genetic model for the minor allele (CC+AC vs. AA) | **128** |
| **Figure S110** | Forest plot of the association between the *EPO* rs551238 polymorphism and diabetic retinopathy in the overall group analysis including only the sets with controls in Hardy-Weinberg equilibrium, under the recessive genetic model for the minor allele (CC vs. AC+AA) | **129** |
| **Figure S111** | Forest plot of the association between the *EPO* rs551238 polymorphism and diabetic retinopathy in the overall group analysis including only the sets with controls in Hardy-Weinberg equilibrium, under the homozygous additive genetic model for the minor allele (CC vs. AA) | **130** |
| **Figure S112** | Forest plot of the association between the *EPO* rs551238 polymorphism and diabetic retinopathy in the overall group analysis including only the sets with controls in Hardy-Weinberg equilibrium, under the heterozygous additive genetic model for the minor allele (AC vs. AA) | **131** |
| **Figure S113** | Forest plot of the association between the *EPO* rs551238 polymorphism and diabetic retinopathy in the overall group analysis including only the sets with controls in Hardy-Weinberg equilibrium, under the overdominant genetic model (AC vs. CC+AA) | **132** |
| **Figure S114** | Forest plot of the association between the *EPO* rs551238 polymorphism and diabetic retinopathy in the overall group analysis including only the sets with controls in Hardy-Weinberg equilibrium, under the allele contrast genetic model (C vs. A) | **133** |
| **Figure S115** | Forest plot of the association between the *EPO* rs551238 polymorphism and diabetic retinopathy in subjects with type 2 diabetes, including only the sets with controls in Hardy-Weinberg equilibrium, under the dominant genetic model for the minor allele (CC+AC vs. AA) | **134** |
| **Figure S116** | Forest plot of the association between the *EPO* rs551238 polymorphism and diabetic retinopathy in subjects with type 2 diabetes, including only the sets with controls in Hardy-Weinberg equilibrium, under the recessive genetic model for the minor allele (CC vs. AC+AA) | **135** |
| **Figure S117** | Forest plot of the association between the *EPO* rs551238 polymorphism and diabetic retinopathy in subjects with type 2 diabetes, including only the sets with controls in Hardy-Weinberg equilibrium, under the homozygous additive genetic model for the minor allele (CC vs. AA) | **136** |
| **Figure S118** | Forest plot of the association between the *EPO* rs551238 polymorphism and diabetic retinopathy in subjects with type 2 diabetes, including only the sets with controls in Hardy-Weinberg equilibrium, under the heterozygous additive genetic model for the minor allele (AC vs. AA) | **137** |
| **Figure S119** | Forest plot of the association between the *EPO* rs551238 polymorphism and diabetic retinopathy in subjects with type 2 diabetes, including only the sets with controls in Hardy-Weinberg equilibrium, under the overdominant genetic model (AC vs. CC+AA) | **138** |
| **Figure S120** | Forest plot of the association between the *EPO* rs551238 polymorphism and diabetic retinopathy in subjects with type 2 diabetes, including only the sets with controls in Hardy-Weinberg equilibrium, under the allele contrast genetic model (C vs. A) | **139** |
| **Figure S121** | Forest plot of the association between the *EPO* rs551238 polymorphism and diabetic retinopathy in non-Asians, including only the sets with controls in Hardy-Weinberg equilibrium, under the dominant genetic model for the minor allele (CC+AC vs. AA) | **140** |
| **Figure S122** | Forest plot of the association between the *EPO* rs551238 polymorphism and diabetic retinopathy in non-Asians, including only the sets with controls in Hardy-Weinberg equilibrium, under the recessive genetic model for the minor allele (CC vs. AC+AA) | **141** |
| **Figure S123** | Forest plot of the association between the *EPO* rs551238 polymorphism and diabetic retinopathy in non-Asians, including only the sets with controls in Hardy-Weinberg equilibrium, under the homozygous additive genetic model for the minor allele (CC vs. AA) | **142** |
| **Figure S124** | Forest plot of the association between the *EPO* rs551238 polymorphism and diabetic retinopathy in non-Asians, including only the sets with controls in Hardy-Weinberg equilibrium, under the heterozygous additive genetic model for the minor allele (AC vs. AA) | **143** |
| **Figure S125** | Forest plot of the association between the *EPO* rs551238 polymorphism and diabetic retinopathy in non-Asians, including only the sets with controls in Hardy-Weinberg equilibrium, under the overdominant genetic model (AC vs. CC+AA) | **144** |
| **Figure S126** | Forest plot of the association between the *EPO* rs551238 polymorphism and diabetic retinopathy in non-Asians, including only the sets with controls in Hardy-Weinberg equilibrium, under the allele contrast genetic model (C vs. A) | **145** |

**
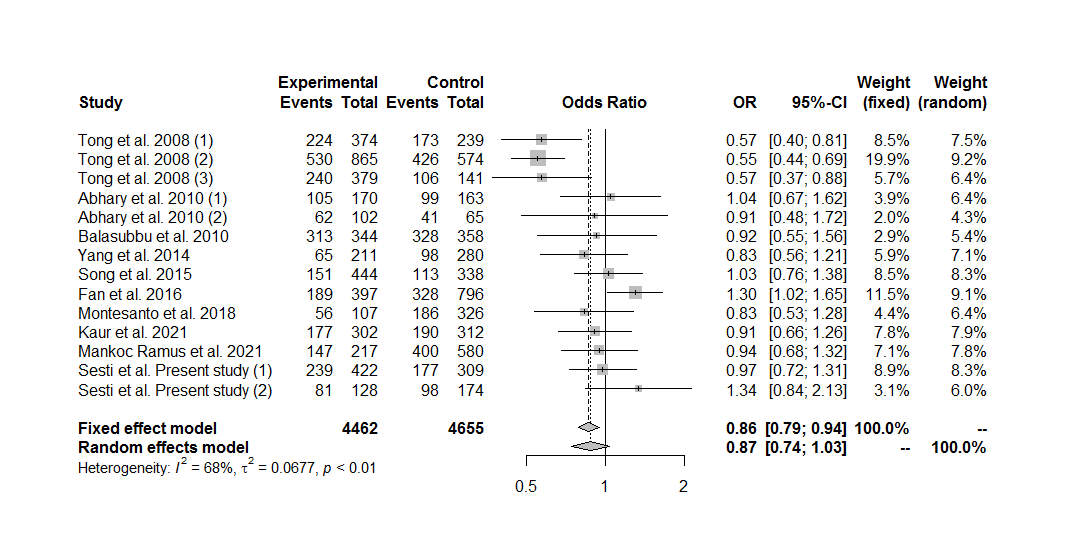
**

**Figure S1.** Forest plot of the association between the *EPO* rs1617640 polymorphism and diabetic retinopathy in the overall group analysis, under the dominant genetic model for the minor allele (GG+TG vs. TT).

**
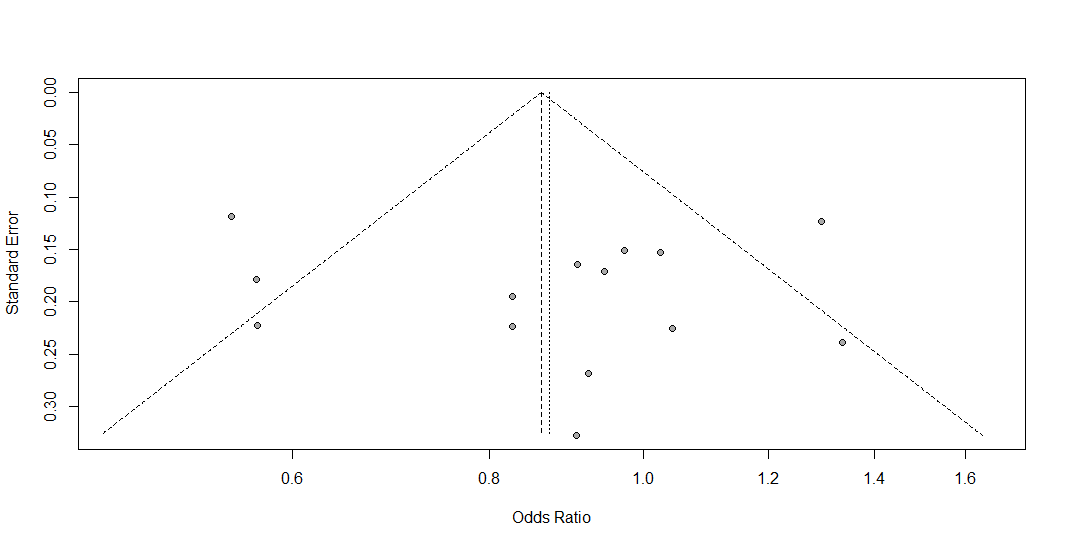
**

**Figure S2.** Funnel plot of the association between the *EPO* rs1617640 polymorphism and diabetic retinopathy in the overall group analysis, under the dominant genetic model for the minor allele (GG+TG vs. TT).

**
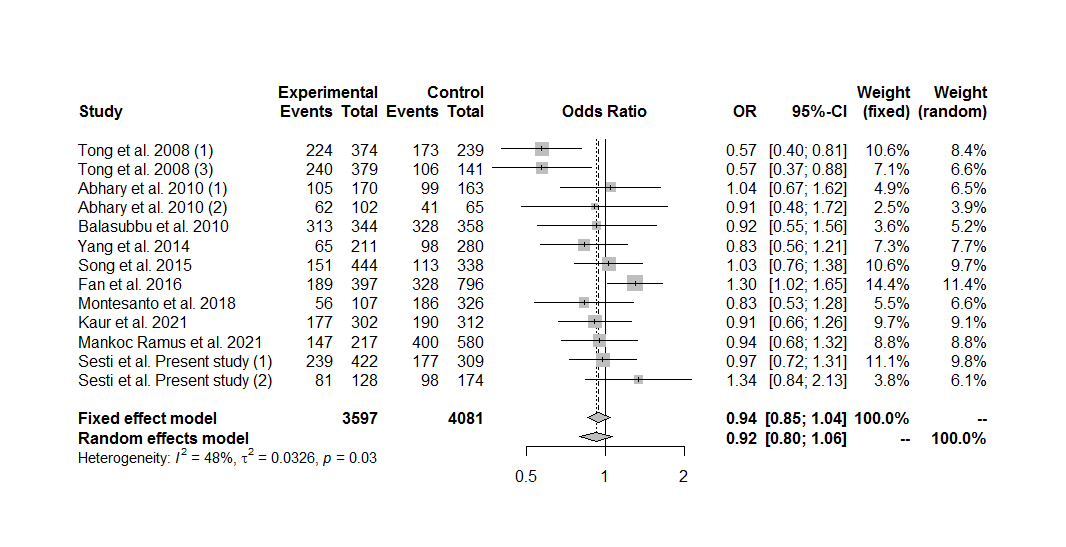
**

**Figure S3.** Forest plot of the association between the *EPO* rs1617640 polymorphism and diabetic retinopathy in the overall group analysis without the cohort #2 by Tong et al. (2008), under the dominant genetic model for the minor allele (GG+TG vs. TT).


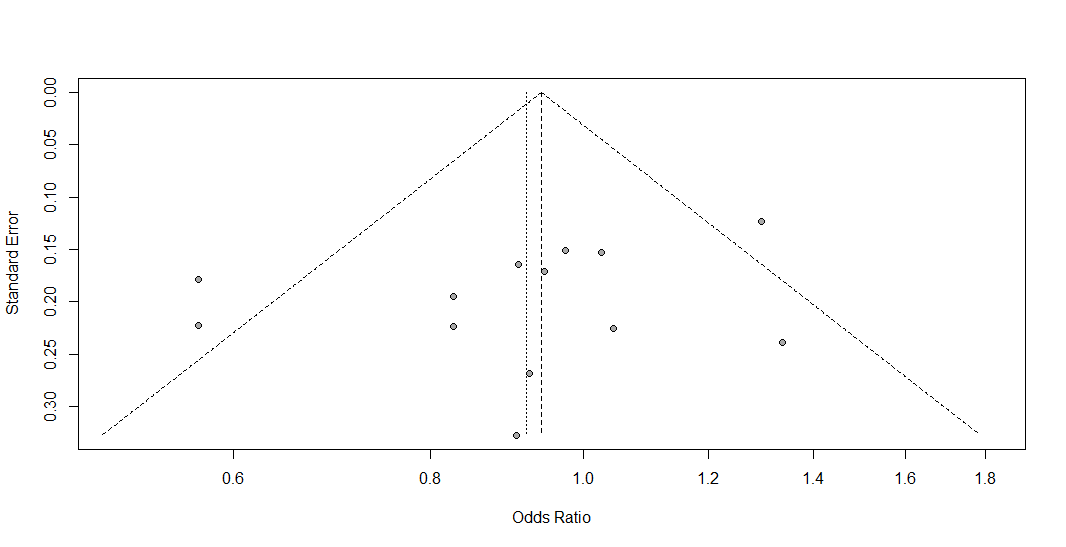


**Figure S4.** Funnel plot of the association between the *EPO* rs1617640 polymorphism and diabetic retinopathy in the overall group analysis without the cohort #2 by Tong et al. (2008), under the dominant genetic model for the minor allele (GG+TG vs. TT).


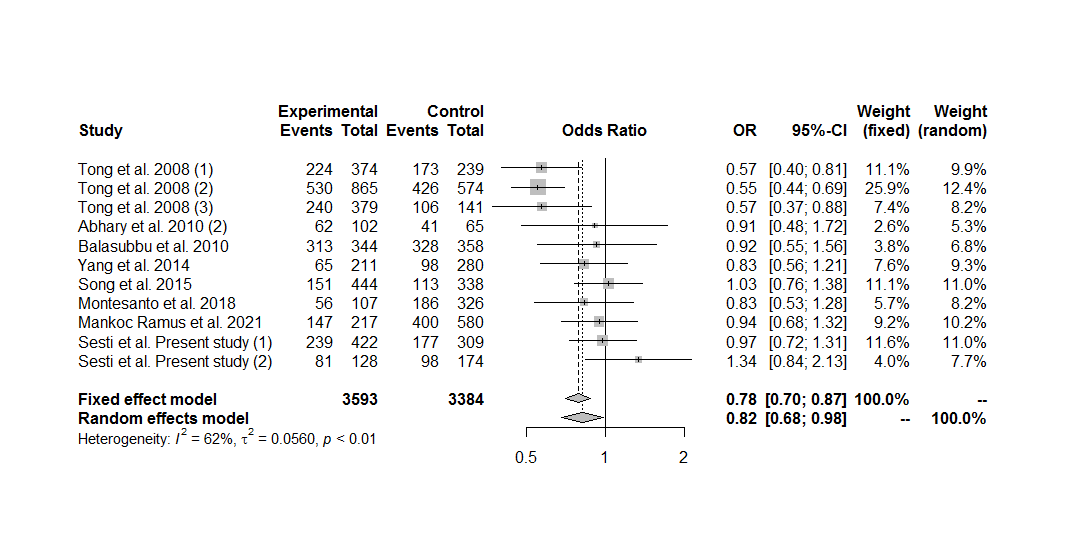


**Figure S5.** Forest plot of the association between the *EPO* rs1617640 polymorphism and diabetic retinopathy in the overall group analysis including only the sets with controls in Hardy-Weinberg equilibrium, under the dominant genetic model for the minor allele (GG+TG vs. TT).

**
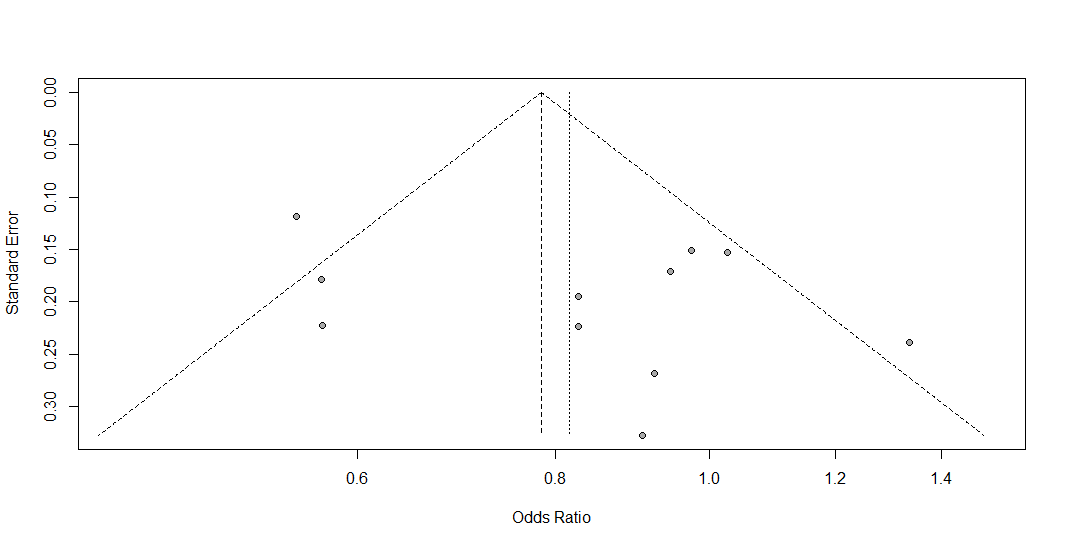
**

**Figure S6.** Funnel plot of the association between the *EPO* rs1617640 polymorphism and diabetic retinopathy in the overall group analysis including only the sets with controls in Hardy-Weinberg equilibrium, under the dominant genetic model for the minor allele (GG+TG vs. TT).


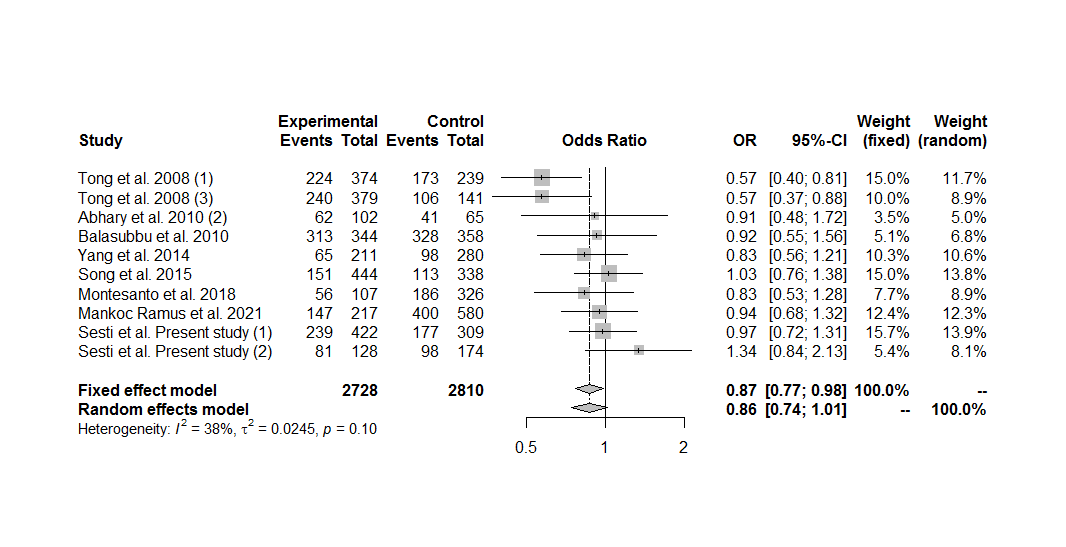


**Figure S7.** Forest plot of the association between the *EPO* rs1617640 polymorphism and diabetic retinopathy in the overall group analysis, including only the sets with controls in Hardy-Weinberg equilibrium and excluding the cohort #2 by Tong et al. (2008), under the dominant genetic model for the minor allele (GG+TG vs. TT).

**
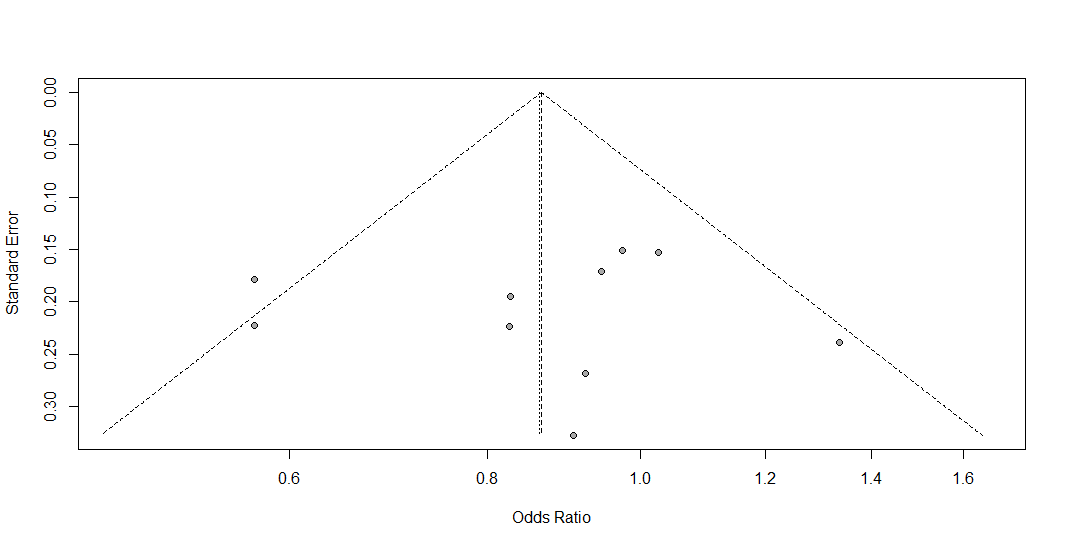
**

**Figure S8.** Funnel plot of the association between the *EPO* rs1617640 polymorphism and diabetic retinopathy in the overall group analysis, including only the sets with controls in Hardy-Weinberg equilibrium and excluding the cohort #2 by Tong et al. (2008), under the dominant genetic model for the minor allele (GG+TG vs. TT).

**
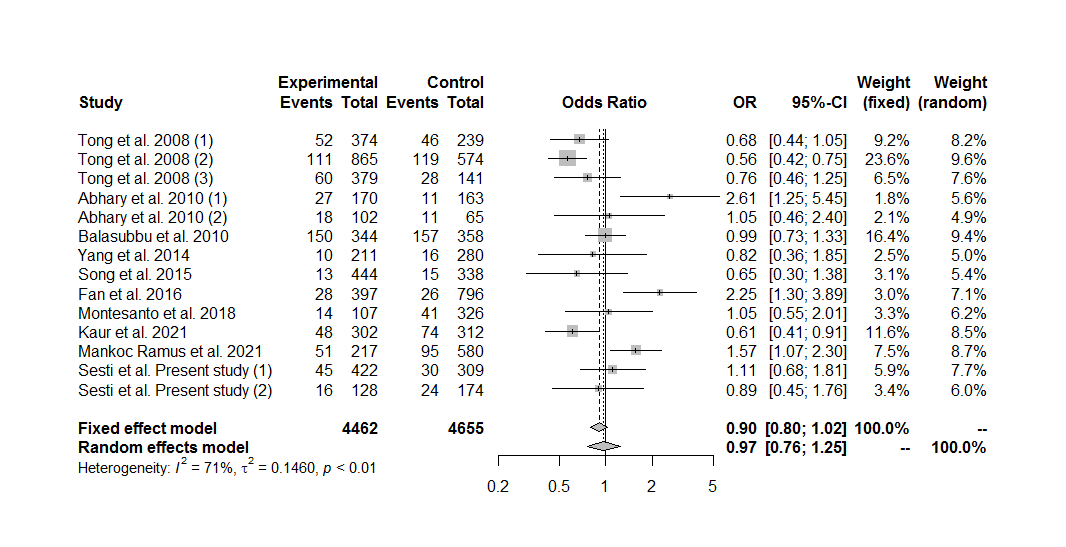
**

**Figure S9.** Forest plot of the association between the *EPO* rs1617640 polymorphism and diabetic retinopathy in the overall group analysis, under the recessive genetic model for the minor allele (GG vs. TG+TT).

**
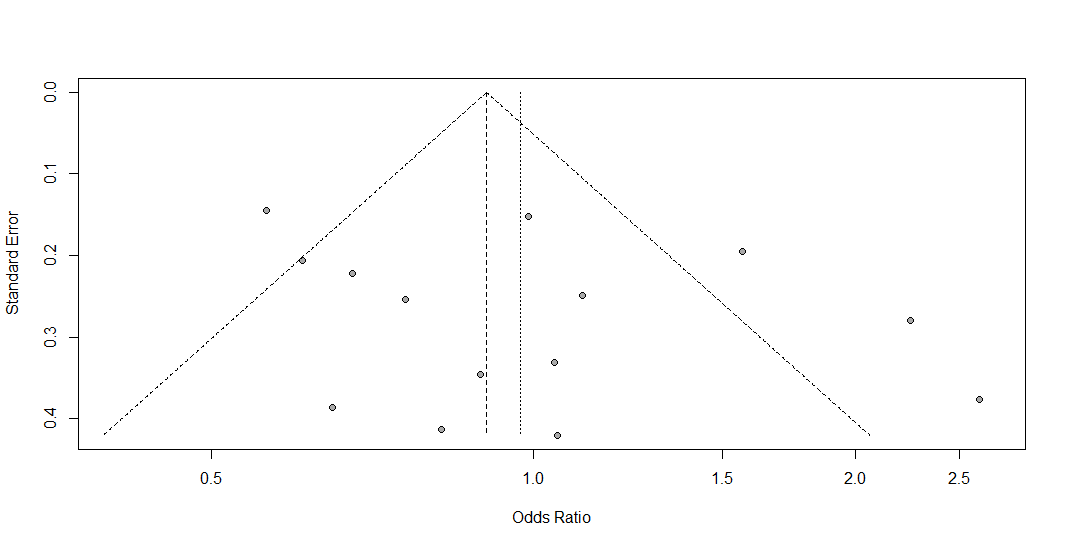
**

**Figure S10.** Funnel plot of the association between the *EPO* rs1617640 polymorphism and diabetic retinopathy in the overall group analysis, under the recessive genetic model for the minor allele (GG vs. TG+TT).

**
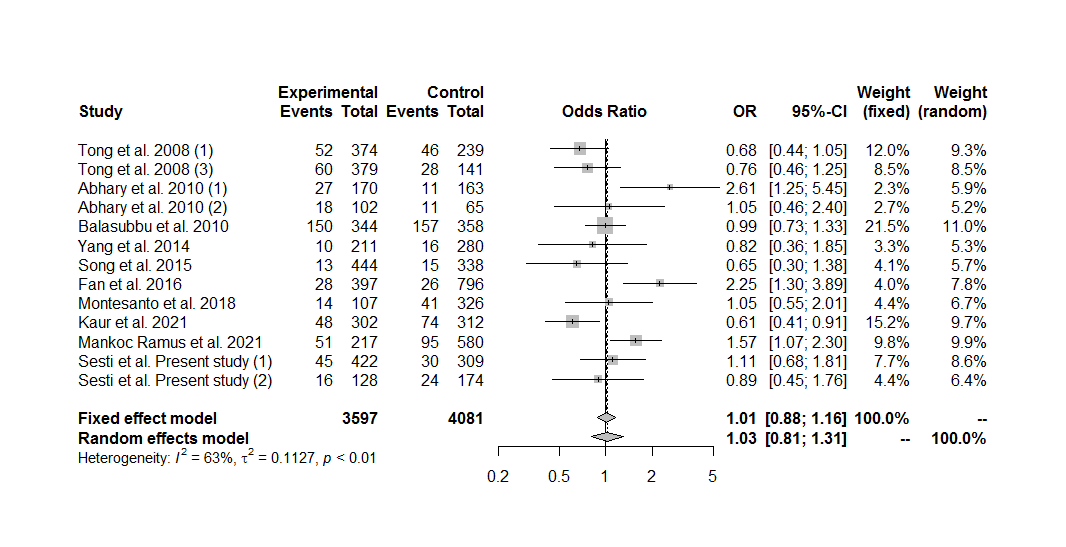
**

**Figure S11.** Forest plot of the association between the *EPO* rs1617640 polymorphism and diabetic retinopathy in the overall group analysis without the cohort #2 by Tong et al. (2008), under the recessive genetic model for the minor allele (GG vs. TG+TT).

**
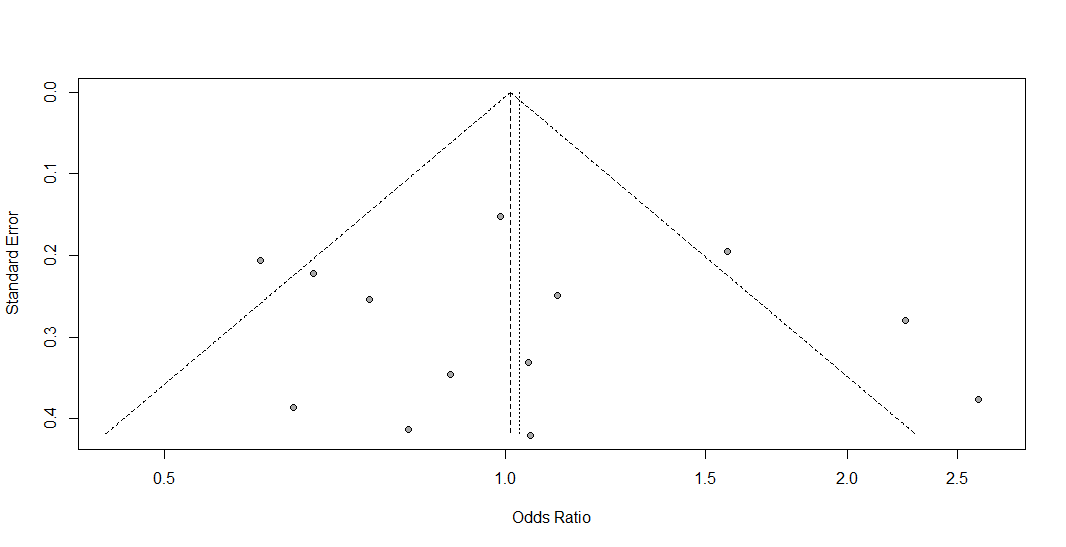
**

**Figure S12.** Funnel plot of the association between the *EPO* rs1617640 polymorphism and diabetic retinopathy in the overall group analysis without the cohort #2 by Tong et al. (2008), under the recessive genetic model for the minor allele (GG vs. TG+TT).

**
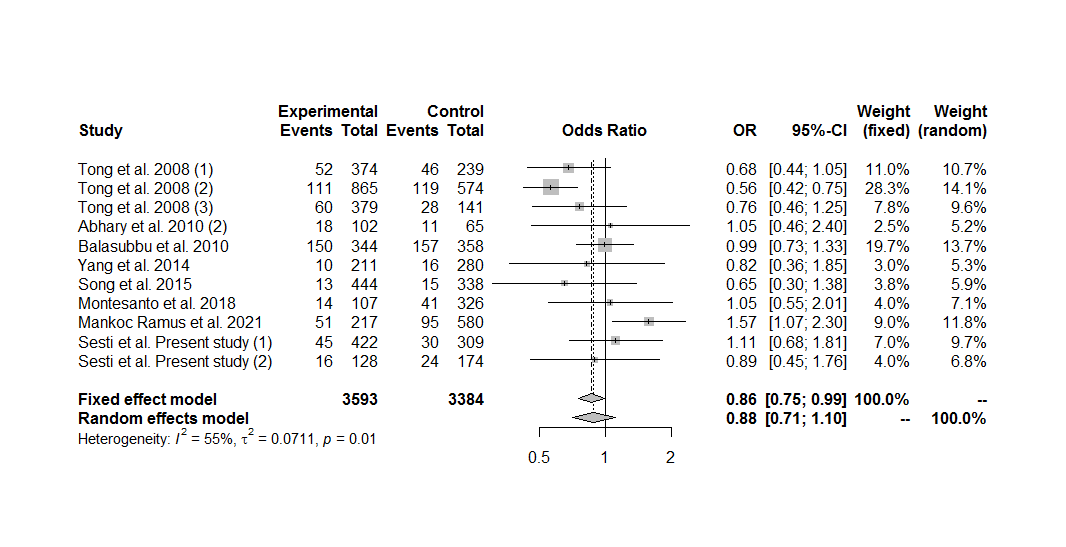
**

**Figure S13.** Forest plot of the association between the *EPO* rs1617640 polymorphism and diabetic retinopathy in the overall group analysis including only the sets with controls in Hardy-Weinberg equilibrium, under the recessive genetic model for the minor allele (GG vs. TG+TT).

**
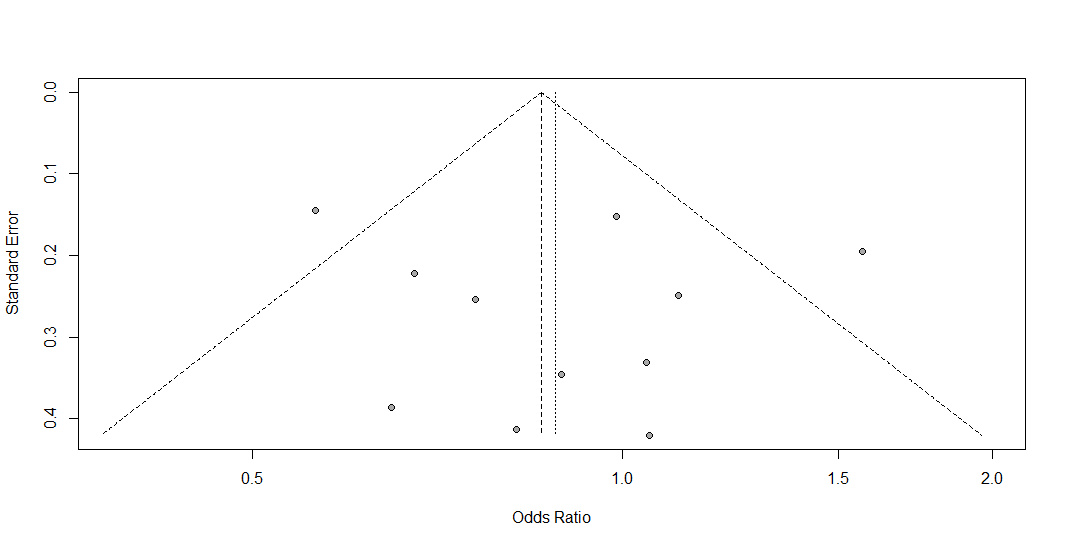
**

**Figure S14.** Funnel plot of the association between the *EPO* rs1617640 polymorphism and diabetic retinopathy in the overall group analysis including only the sets with controls in Hardy-Weinberg equilibrium, under the recessive genetic model for the minor allele (GG vs. TG+TT).

**
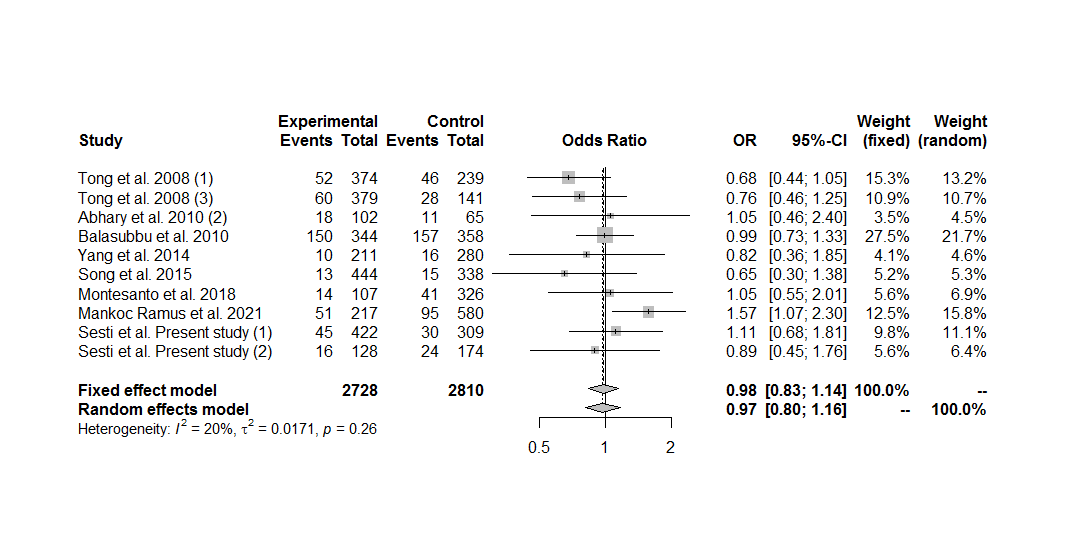
**

**Figure S15.** Forest plot of the association between the *EPO* rs1617640 polymorphism and diabetic retinopathy in the overall group analysis, including only the sets with controls in Hardy-Weinberg equilibrium and excluding the cohort #2 by Tong et al. (2008), under the recessive genetic model for the minor allele (GG vs. TG+TT).

**
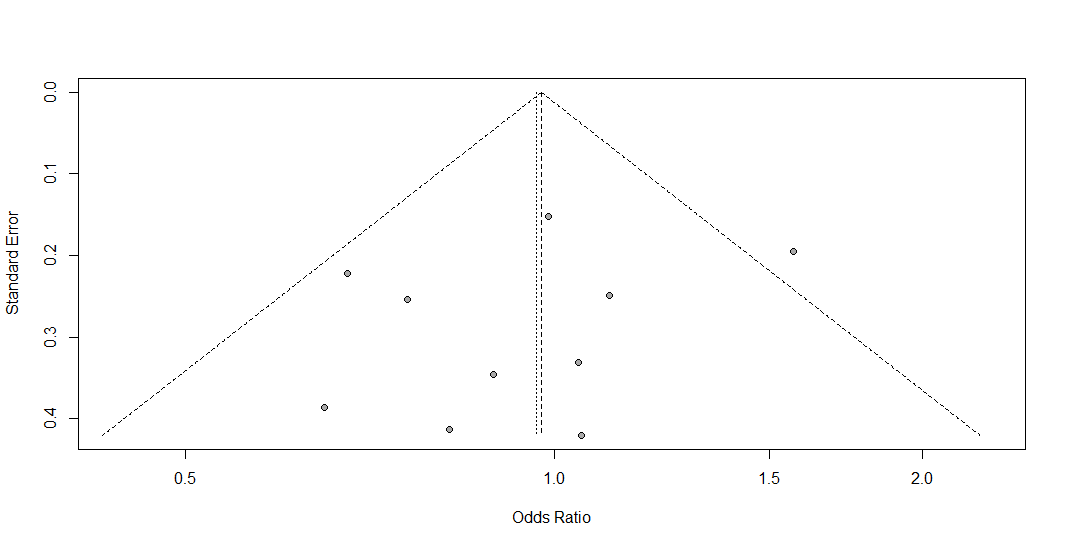
**

**Figure S16.** Funnel plot of the association between the *EPO* rs1617640 polymorphism and diabetic retinopathy in the overall group analysis, including only the sets with controls in Hardy-Weinberg equilibrium and excluding the cohort #2 by Tong et al. (2008), under the recessive genetic model for the minor allele (GG vs. TG+TT).

**
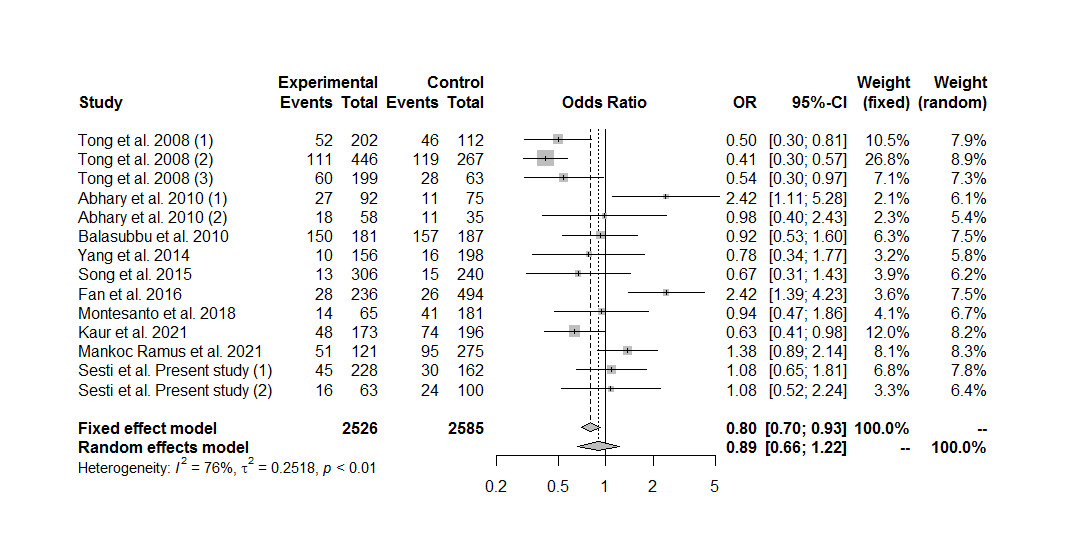
**

**Figure S17.** Forest plot of the association between the *EPO* rs1617640 polymorphism and diabetic retinopathy in the overall group analysis, under the homozygous additive genetic model for the minor allele (GG vs. TT).

**
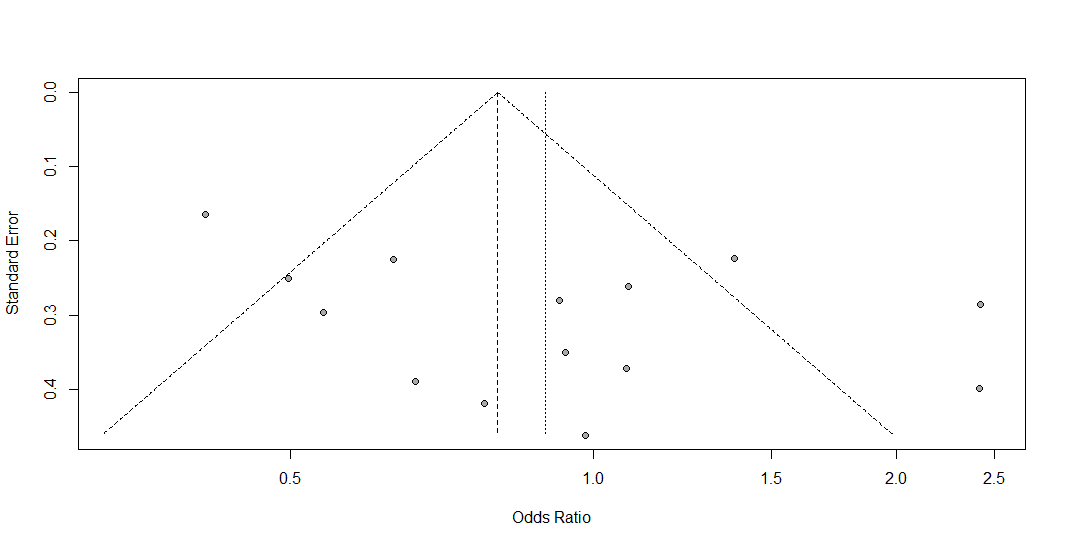
**

**Figure S18.** Funnel plot of the association between the *EPO* rs1617640 polymorphism and diabetic retinopathy in the overall group analysis, under the homozygous additive genetic model for the minor allele (GG vs. TT).

**
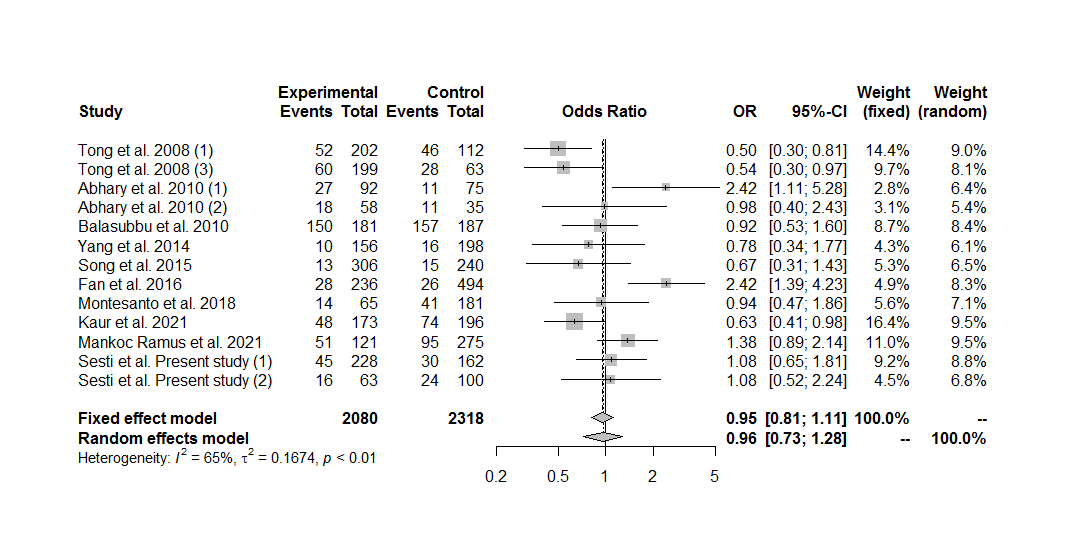
**

**Figure S19.** Forest plot of the association between the *EPO* rs1617640 polymorphism and diabetic retinopathy in the overall group analysis without the cohort #2 by Tong et al. (2008), under the homozygous additive genetic model for the minor allele (GG vs. TT).

**
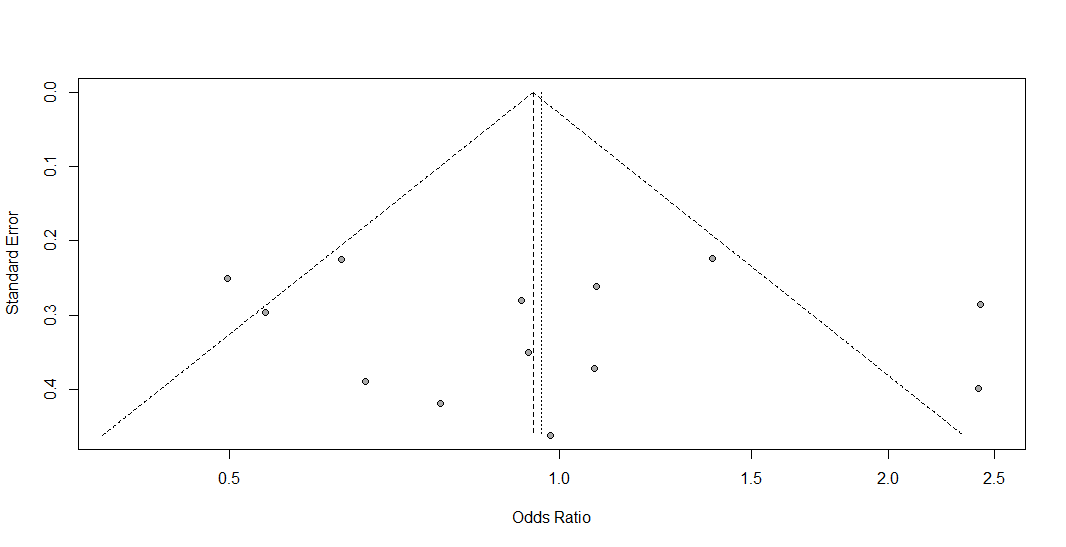
**

**Figure S20.** Funnel plot of the association between the *EPO* rs1617640 polymorphism and diabetic retinopathy in the overall group analysis without the cohort #2 by Tong et al. (2008), under the homozygous additive genetic model for the minor allele (GG vs. TT).

**
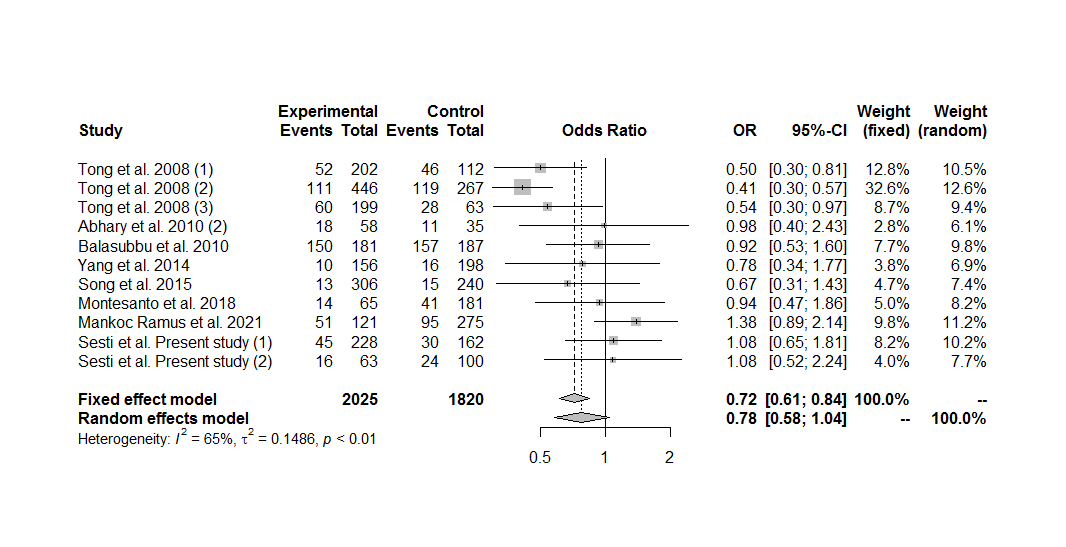
**

**Figure S21.** Forest plot of the association between the *EPO* rs1617640 polymorphism and diabetic retinopathy in the overall group analysis including only the sets with controls in Hardy-Weinberg equilibrium, under the homozygous additive genetic model for the minor allele (GG vs. TT).

**
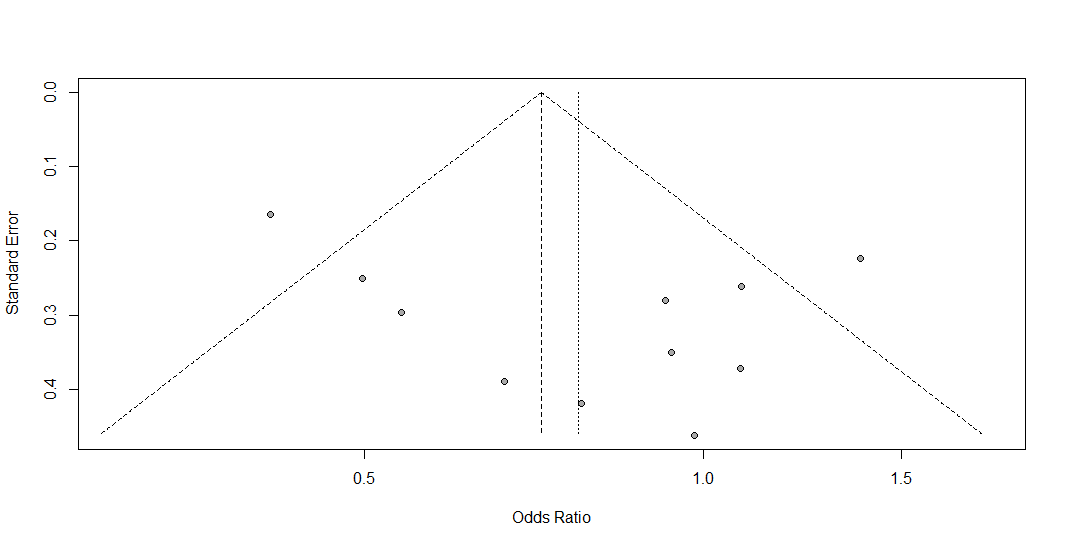
**

**Figure S22.** Funnel plot of the association between the *EPO* rs1617640 polymorphism and diabetic retinopathy in the overall group analysis including only the sets with controls in Hardy-Weinberg equilibrium, under the homozygous additive genetic model for the minor allele (GG vs. TT).

**
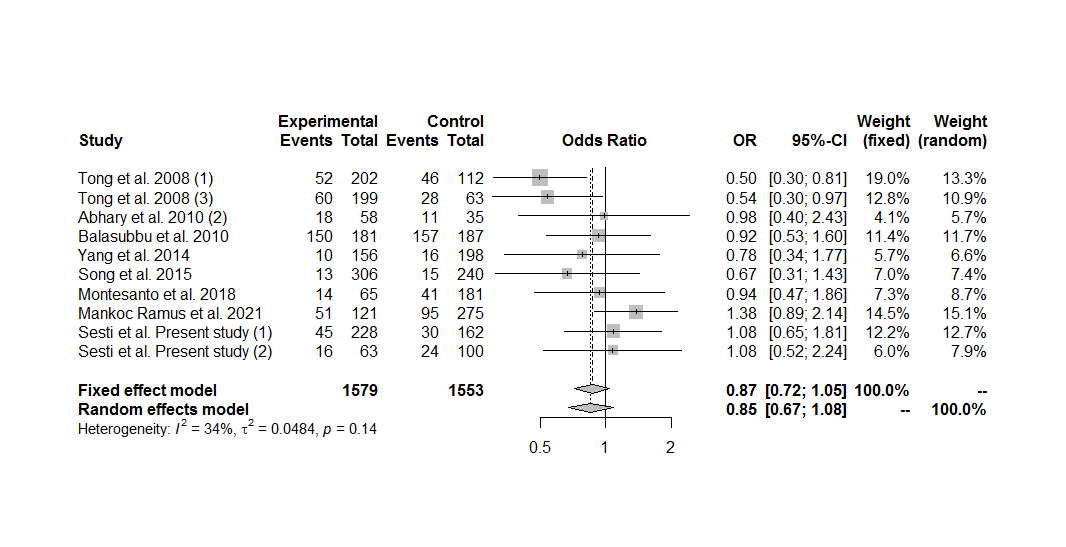
**

**Figure S23.** Forest plot of the association between the *EPO* rs1617640 polymorphism and diabetic retinopathy in the overall group analysis, including only the sets with controls in Hardy-Weinberg equilibrium and excluding the cohort #2 by Tong et al. (2008), under the homozygous additive genetic model for the minor allele (GG vs. TT).

**
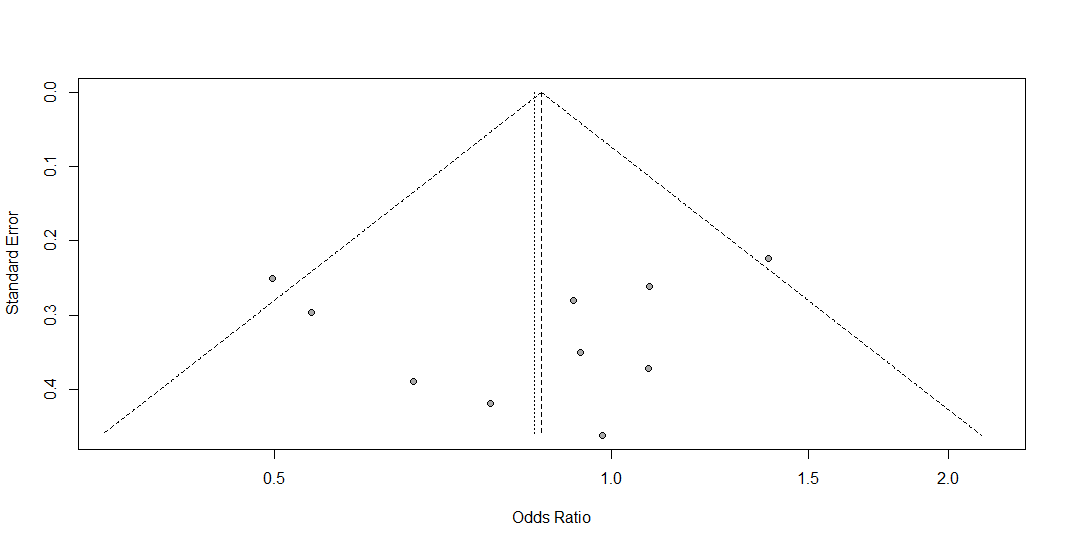
**

**Figure S24.** Funnel plot of the association between the *EPO* rs1617640 polymorphism and diabetic retinopathy in the overall group analysis, including only the sets with controls in Hardy-Weinberg equilibrium and excluding the cohort #2 by Tong et al. (2008), under the homozygous additive genetic model for the minor allele (GG vs. TT).

**
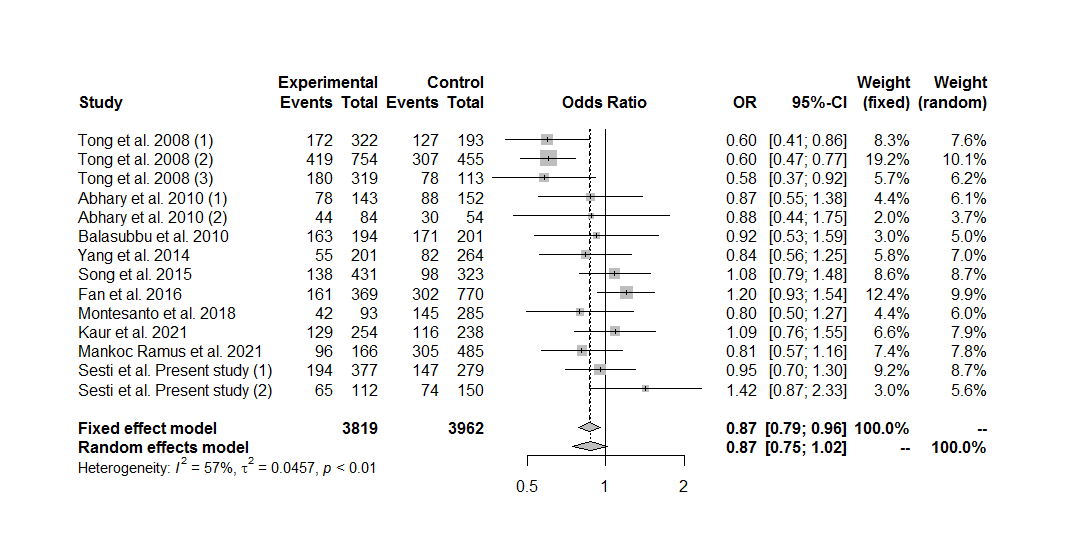
**

**Figure S25.** Forest plot of the association between the *EPO* rs1617640 polymorphism and diabetic retinopathy in the overall group analysis, under the heterozygous additive genetic model for the minor allele (TG vs. TT).

**
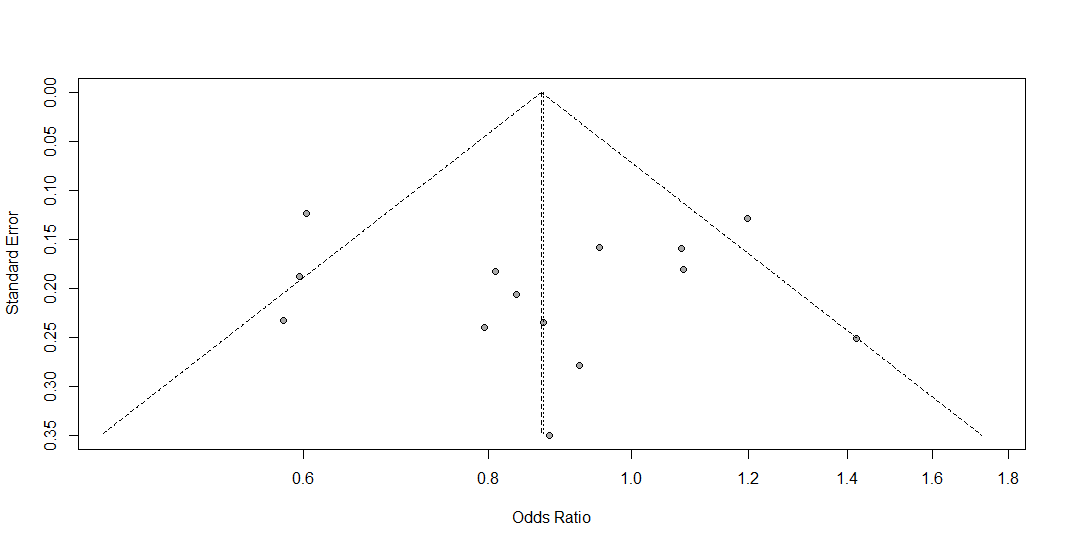
**

**Figure S26.** Funnel plot of the association between the *EPO* rs1617640 polymorphism and diabetic retinopathy in the overall group analysis, under the heterozygous additive genetic model for the minor allele (TG vs. TT).

**
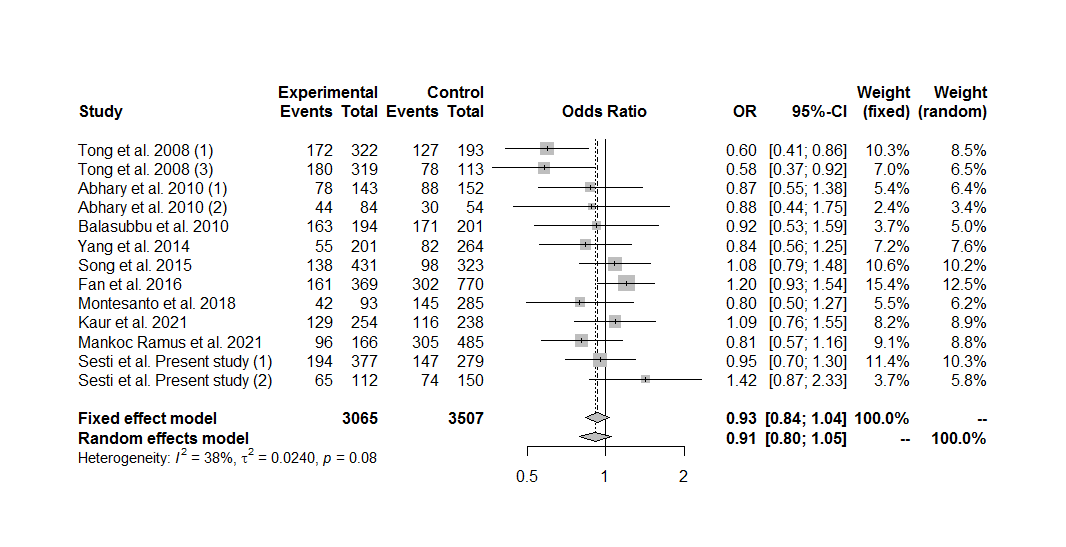
**

**Figure S27.** Forest plot of the association between the *EPO* rs1617640 polymorphism and diabetic retinopathy in the overall group analysis without the cohort #2 by Tong et al. (2008), under the heterozygous additive genetic model for the minor allele (TG vs. TT).

**
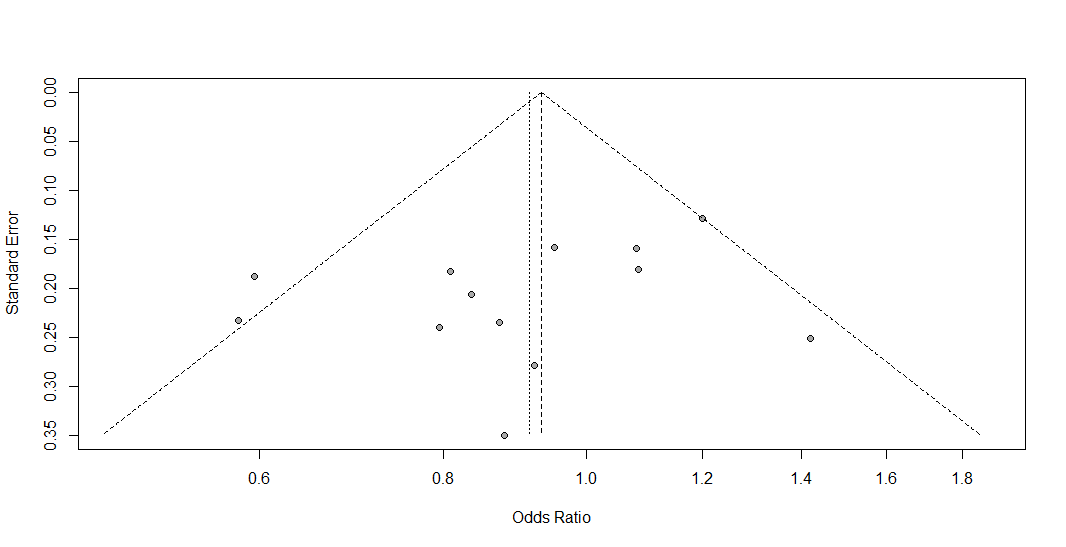
**

**Figure S28.** Funnel plot of the association between the *EPO* rs1617640 polymorphism and diabetic retinopathy in the overall group analysis without the cohort #2 by Tong et al. (2008), under the heterozygous additive genetic model for the minor allele (TG vs. TT).

**
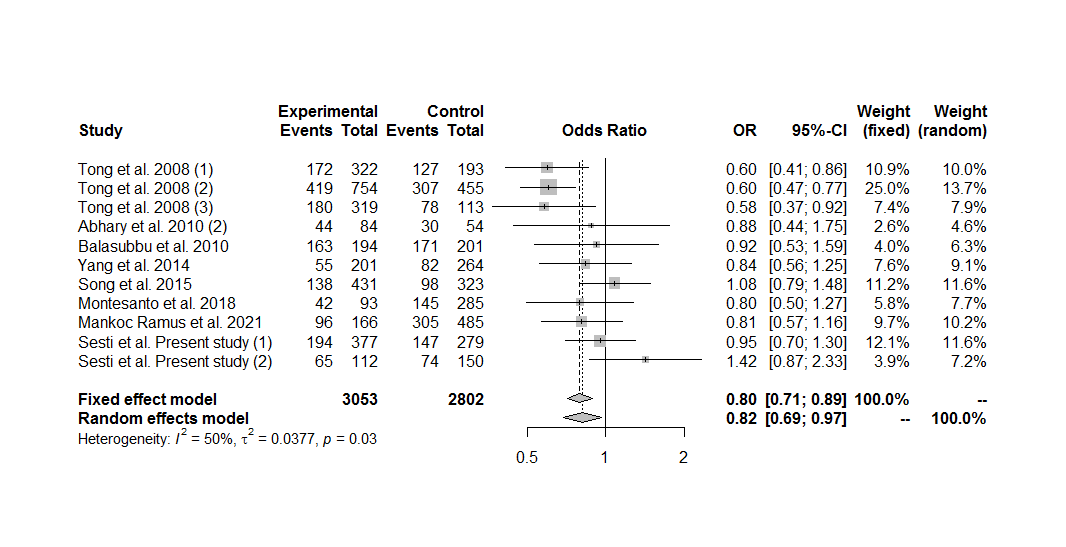
**

**Figure S29.** Forest plot of the association between the *EPO* rs1617640 polymorphism and diabetic retinopathy in the overall group analysis including only the sets with controls in Hardy-Weinberg equilibrium, under the heterozygous additive genetic model for the minor allele (TG vs. TT).

**
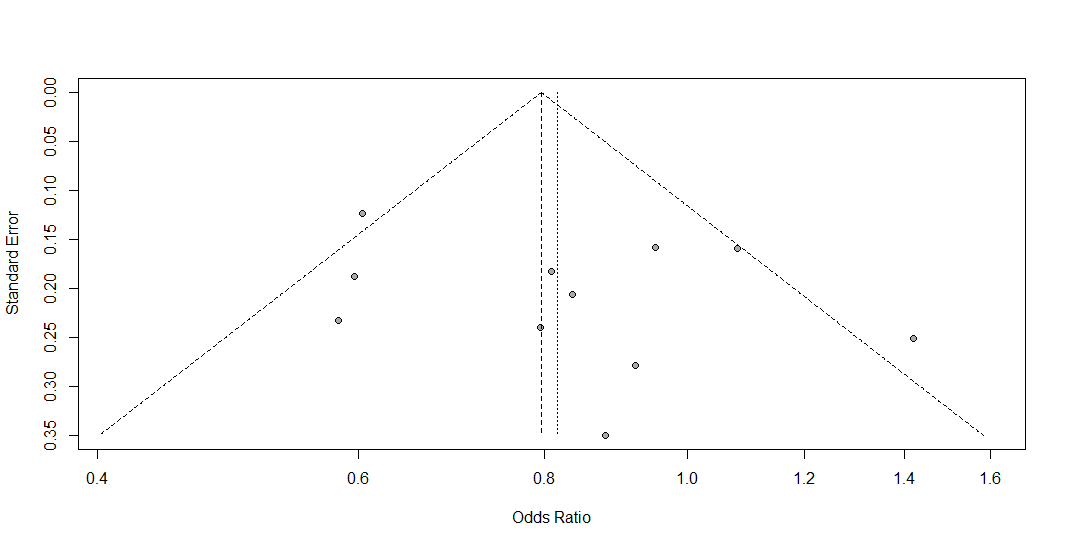
**

**Figure S30.** Funnel plot of the association between the *EPO* rs1617640 polymorphism and diabetic retinopathy in the overall group analysis including only the sets with controls in Hardy-Weinberg equilibrium, under the heterozygous additive genetic model for the minor allele (TG vs. TT).

**
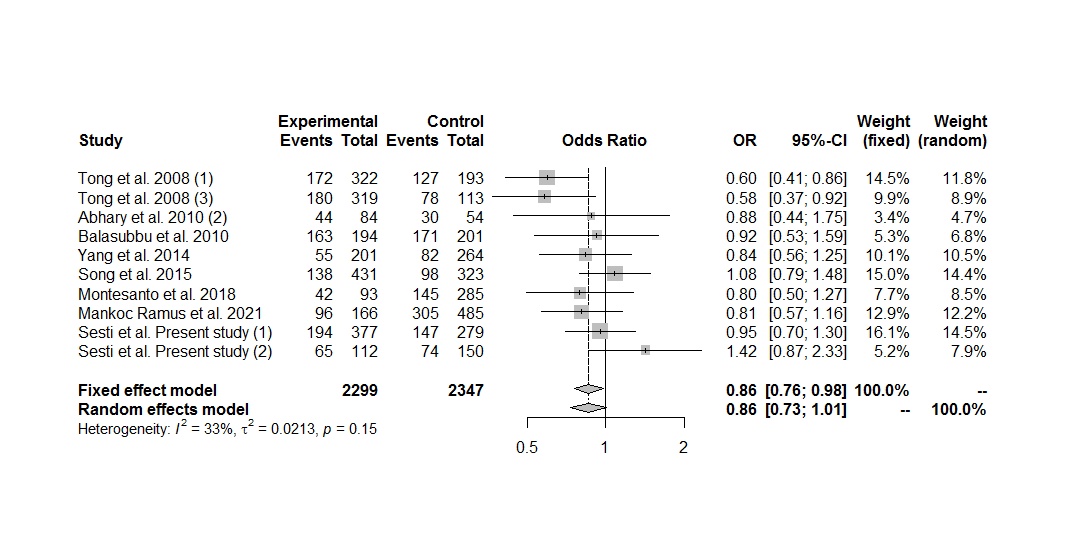
**

**Figure S31.** Forest plot of the association between the *EPO* rs1617640 polymorphism and diabetic retinopathy in the overall group analysis, including only the sets with controls in Hardy-Weinberg equilibrium and excluding the cohort #2 by Tong et al. (2008), under the heterozygous additive genetic model for the minor allele (TG vs. TT).

**
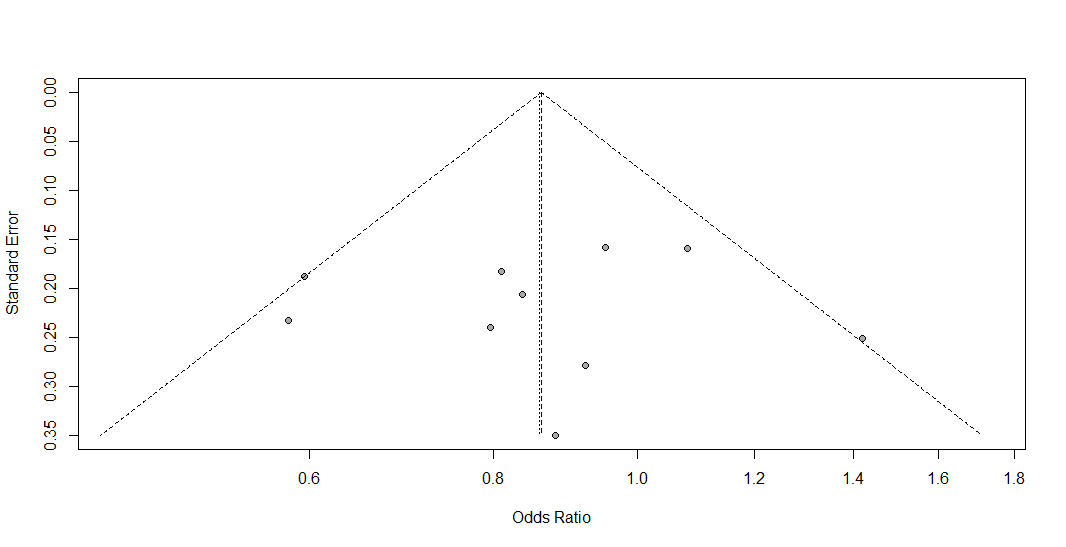
**

**Figure S32.** Funnel plot of the association between the *EPO* rs1617640 polymorphism and diabetic retinopathy in the overall group analysis, including only the sets with controls in Hardy-Weinberg equilibrium and excluding the cohort #2 by Tong et al. (2008), under the heterozygous additive genetic model for the minor allele (TG vs. TT).

**
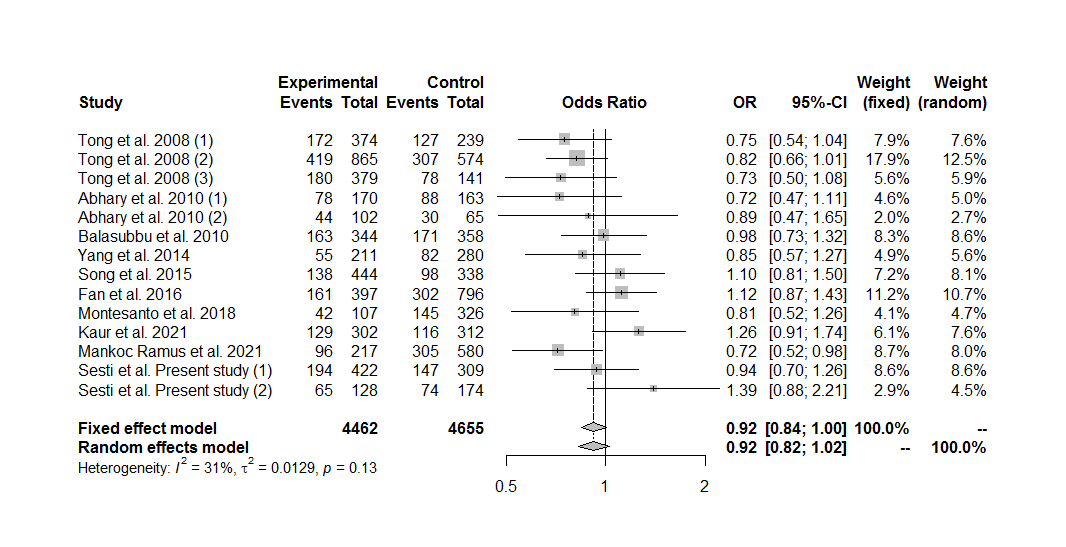
**

**Figure S33.** Forest plot of the association between the *EPO* rs1617640 polymorphism and diabetic retinopathy in the overall group analysis, under the overdominant genetic model (TG vs. GG+TT).

**
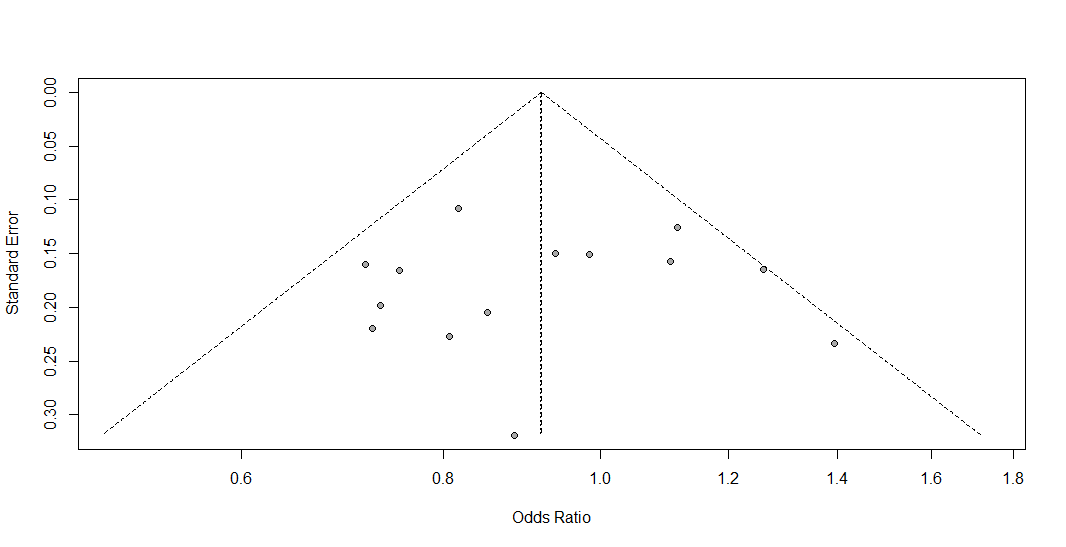
**

**Figure S34.** Funnel plot of the association between the *EPO* rs1617640 polymorphism and diabetic retinopathy in the overall group analysis, under the overdominant genetic model (TG vs. GG+TT).

**
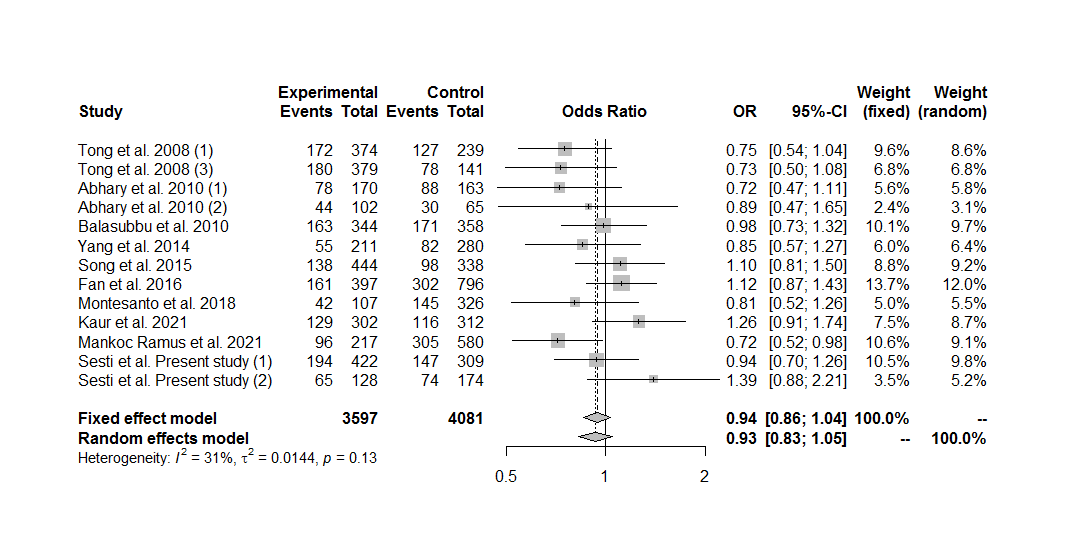
**

**Figure S35.** Forest plot of the association between the *EPO* rs1617640 polymorphism and diabetic retinopathy in the overall group analysis without the cohort #2 by Tong et al. (2008), under the overdominant genetic model (TG vs. GG+TT).

**
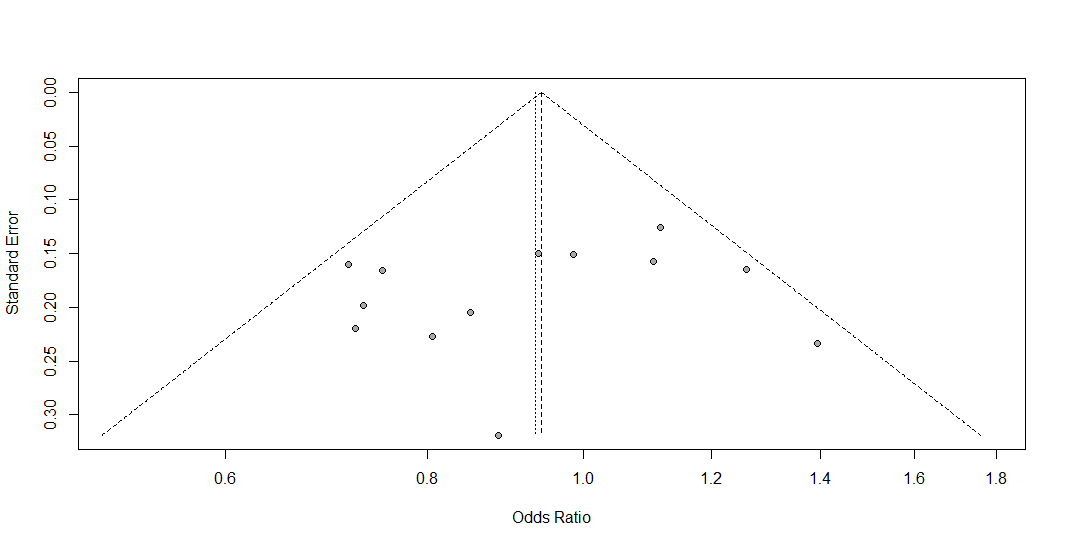
**

**Figure S36.** Funnel plot of the association between the *EPO* rs1617640 polymorphism and diabetic retinopathy in the overall group analysis without the cohort #2 by Tong et al. (2008), under the overdominant genetic model (TG vs. GG+TT).

**
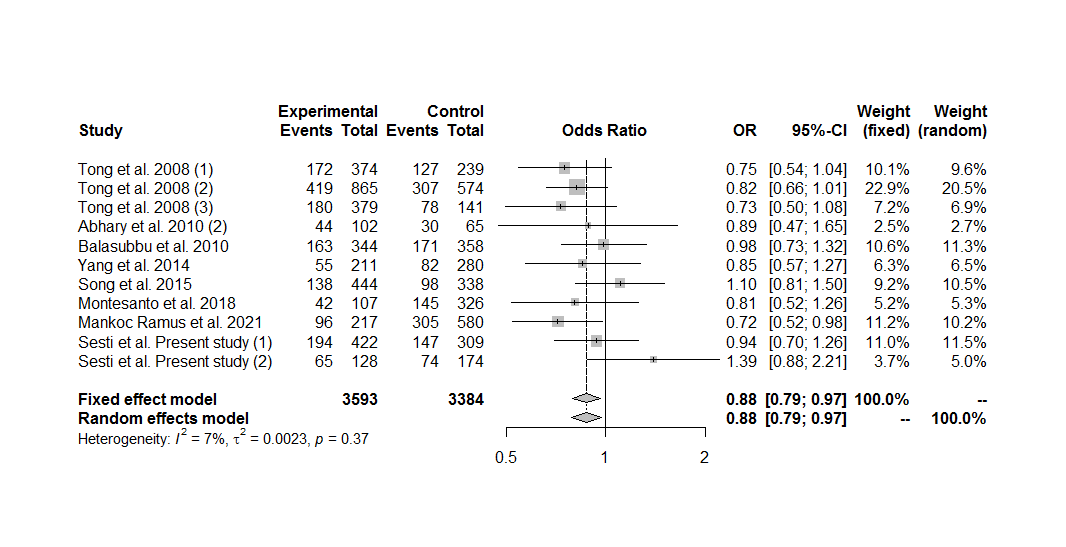
**

**Figure S37.** Forest plot of the association between the *EPO* rs1617640 polymorphism and diabetic retinopathy in the overall group analysis including only the sets with controls in Hardy-Weinberg equilibrium, under the overdominant genetic model (TG vs. GG+TT).

**
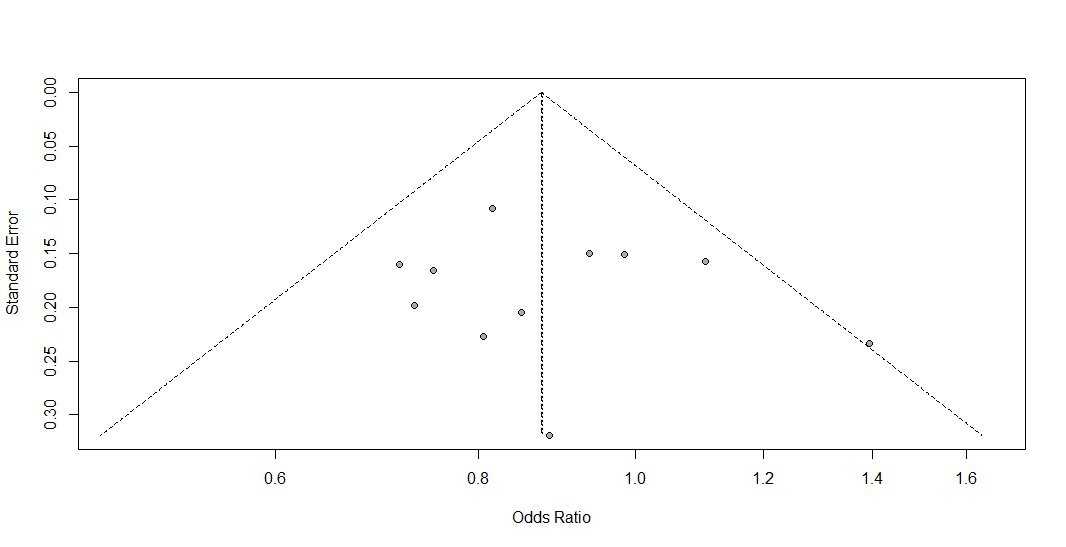
**

**Figure S38.** Funnel plot of the association between the *EPO* rs1617640 polymorphism and diabetic retinopathy in the overall group analysis including only the sets with controls in Hardy-Weinberg equilibrium, under the overdominant genetic model (TG vs. GG+TT).

**
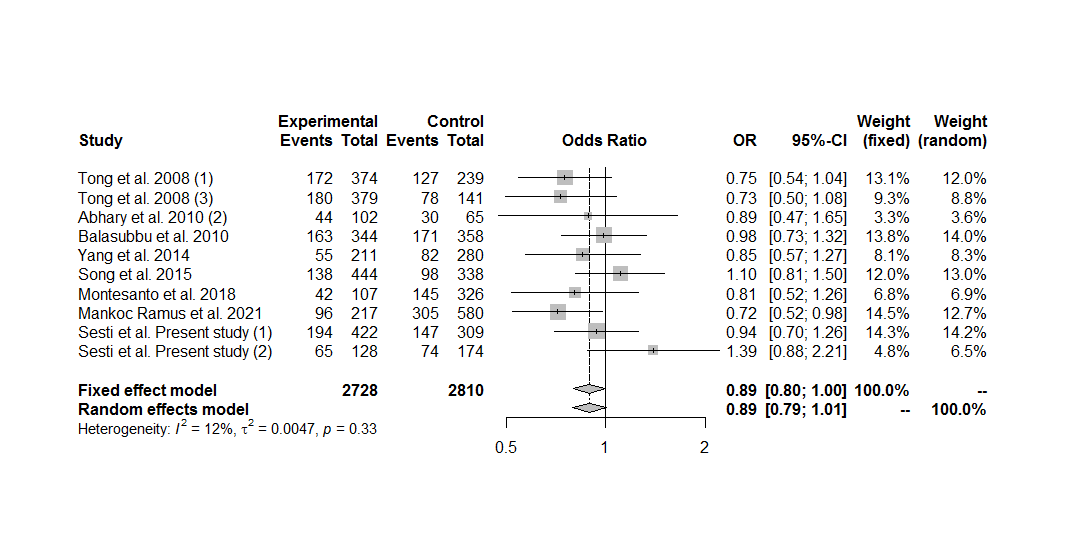
**

**Figure S39.** Forest plot of the association between the *EPO* rs1617640 polymorphism and diabetic retinopathy in the overall group analysis, including only the sets with controls in Hardy-Weinberg equilibrium and excluding the cohort #2 by Tong et al. (2008), under the overdominant genetic model (TG vs. GG+TT).

**
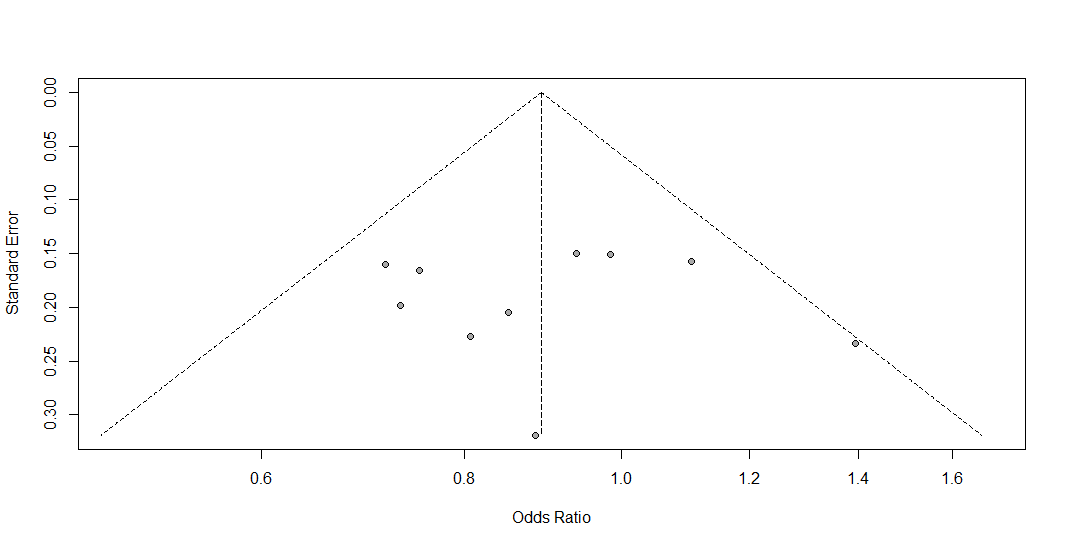
**

**Figure S40.** Funnel plot of the association between the *EPO* rs1617640 polymorphism and diabetic retinopathy in the overall group analysis, including only the sets with controls in Hardy-Weinberg equilibrium and excluding the cohort #2 by Tong et al. (2008), under the overdominant genetic model (TG vs. GG+TT).

**
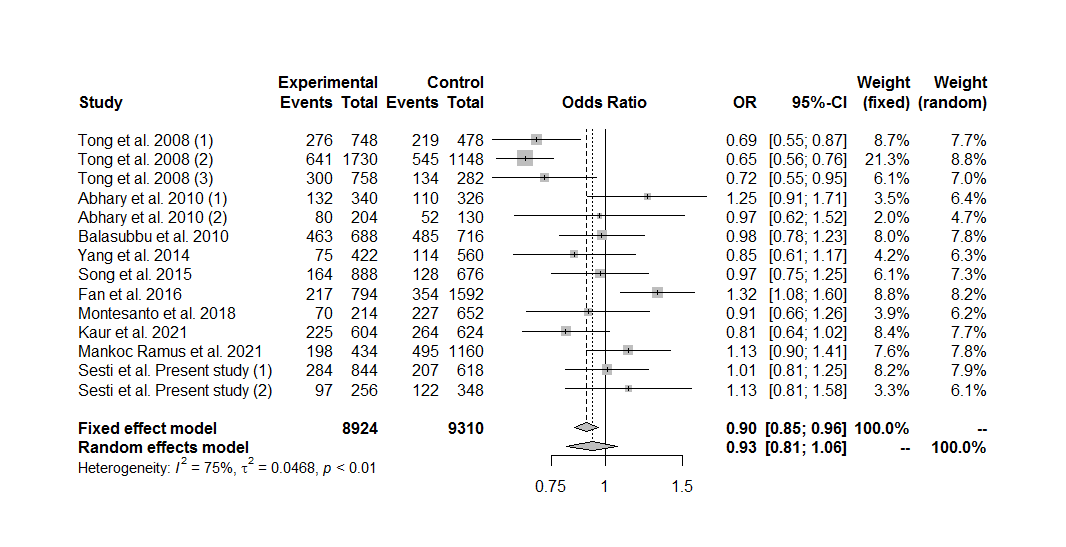
**

**Figure S41.** Forest plot of the association between the *EPO* rs1617640 polymorphism and diabetic retinopathy in the overall group analysis, under the allele contrast genetic model (G vs. T).

**
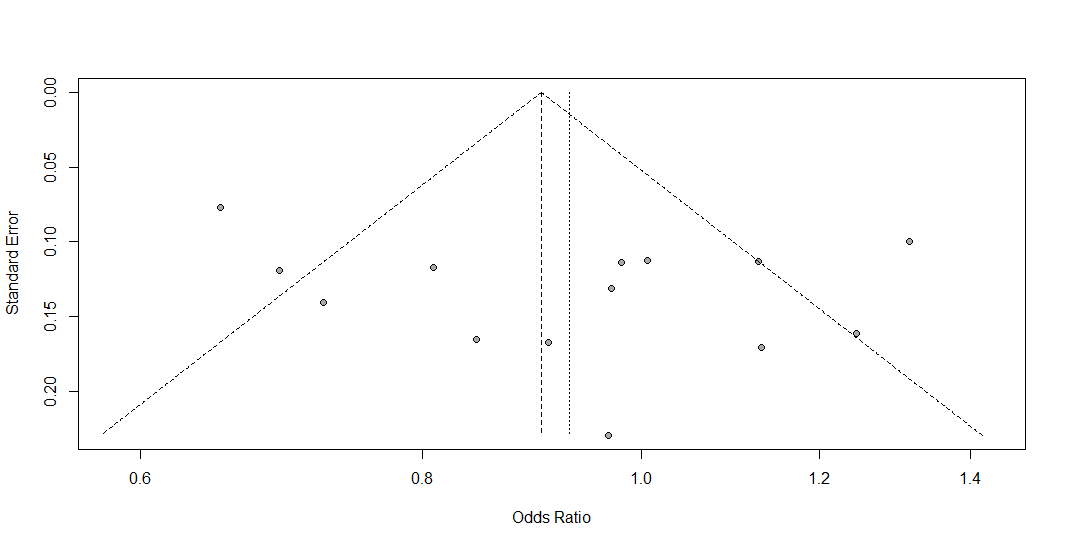
**

**Figure S42.** Funnel plot of the association between the *EPO* rs1617640 polymorphism and diabetic retinopathy in the overall group analysis, under the allele contrast genetic model (G vs. T).

**
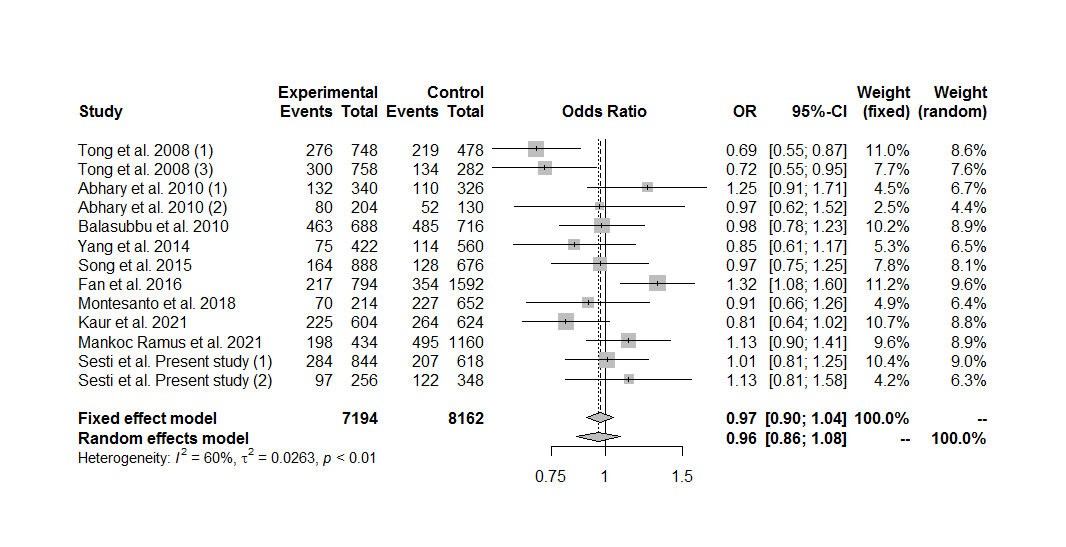
**

**Figure S43.** Forest plot of the association between the *EPO* rs1617640 polymorphism and diabetic retinopathy in the overall group analysis without the cohort #2 by Tong et al. (2008), under the allele contrast genetic model (G vs. T).

**
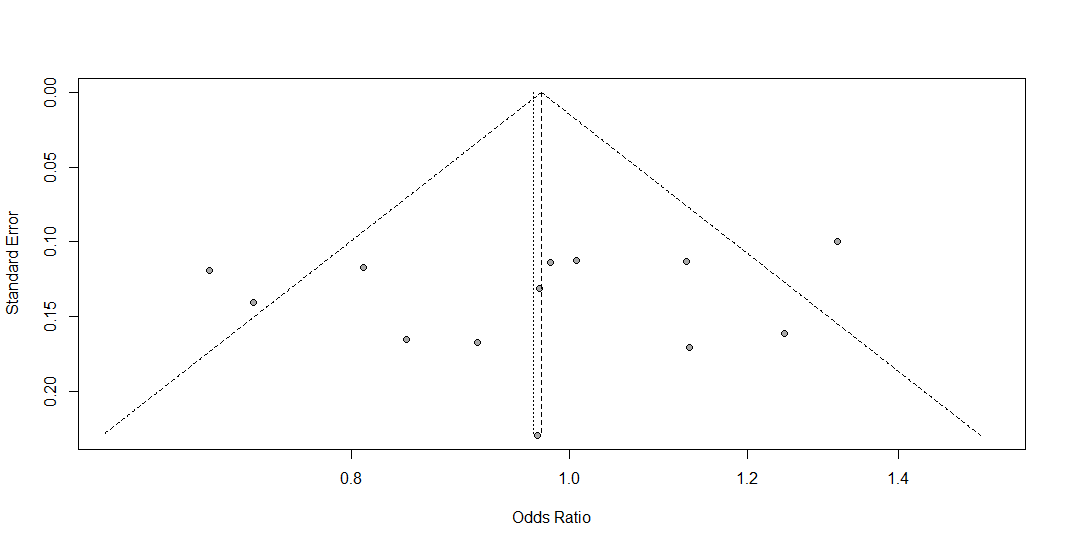
**

**Figure S44.** Funnel plot of the association between the *EPO* rs1617640 polymorphism and diabetic retinopathy in the overall group analysis without the cohort #2 by Tong et al. (2008), under the allele contrast genetic model (G vs. T).

**
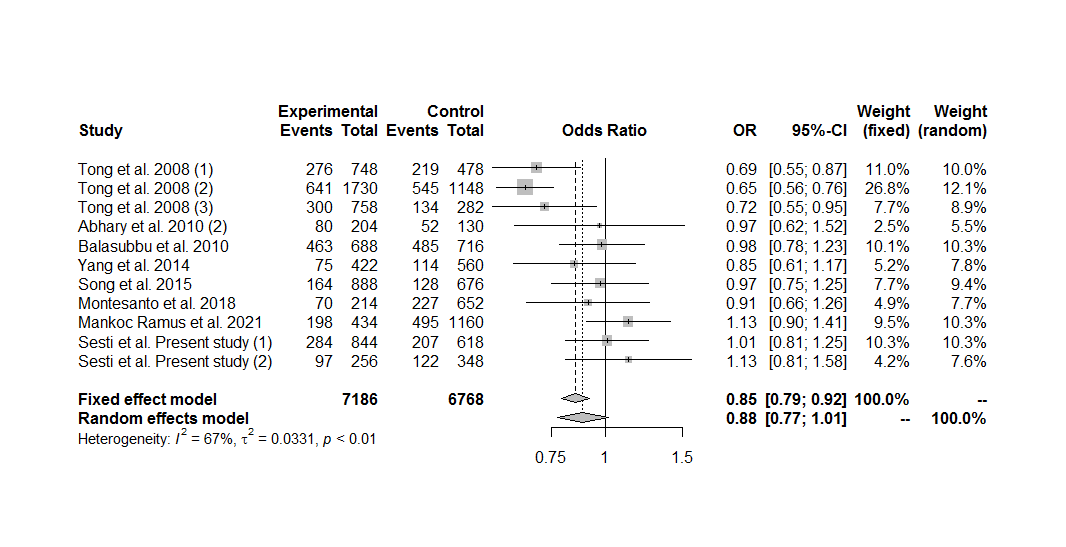
**

**Figure S45.** Forest plot of the association between the *EPO* rs1617640 polymorphism and diabetic retinopathy in the overall group analysis including only the sets with controls in Hardy-Weinberg equilibrium, under the allele contrast genetic model (G vs. T).

**
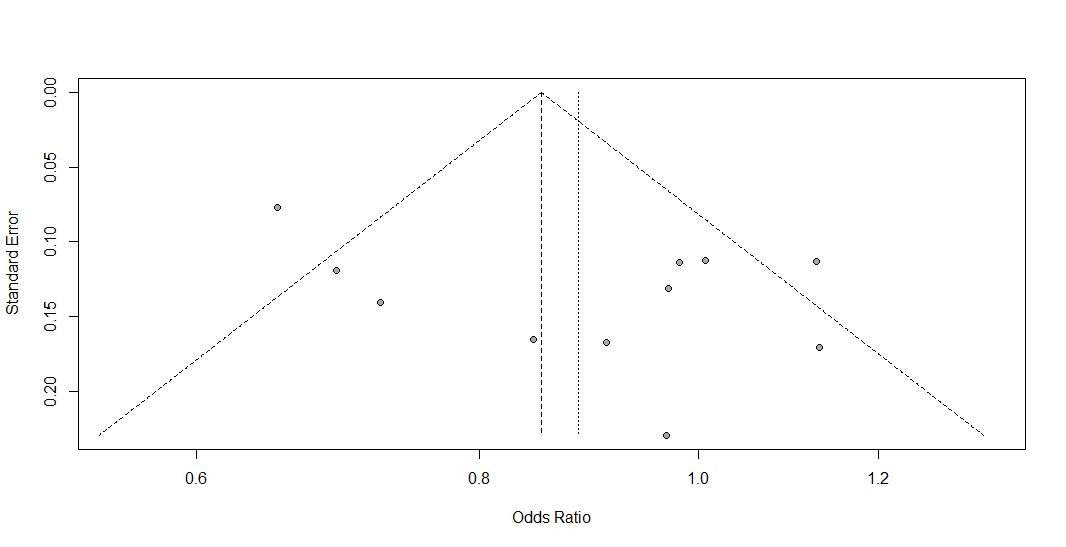
**

**Figure S46.** Funnel plot of the association between the *EPO* rs1617640 polymorphism and diabetic retinopathy in the overall group analysis including only the sets with controls in Hardy-Weinberg equilibrium, under the allele contrast genetic model (G vs. T).

**
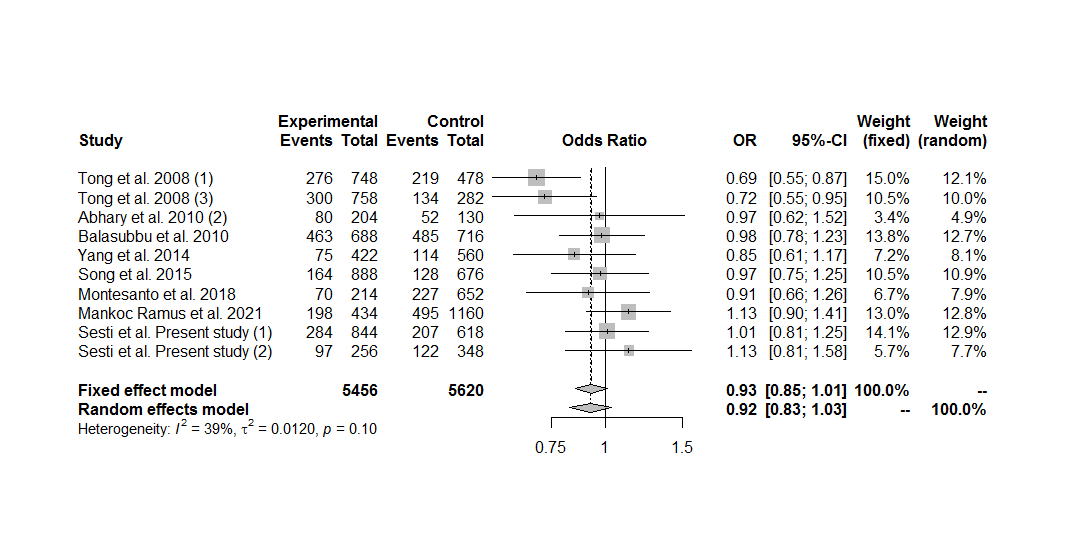
**

**Figure S47.** Forest plot of the association between the *EPO* rs1617640 polymorphism and diabetic retinopathy in the overall group analysis, including only the sets with controls in Hardy-Weinberg equilibrium and excluding the cohort #2 by Tong et al. (2008), under the allele contrast genetic model (G vs. T).

**
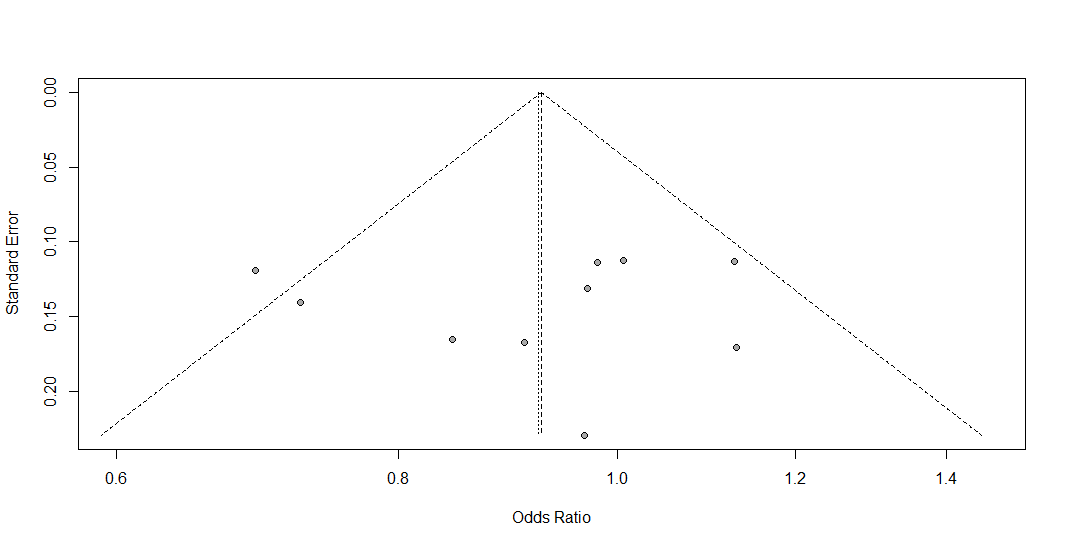
**

**Figure S48.** Funnel plot of the association between the *EPO* rs1617640 polymorphism and diabetic retinopathy in the overall group analysis, including only the sets with controls in Hardy-Weinberg equilibrium and excluding the cohort #2 by Tong et al. (2008), under the allele contrast genetic model (G vs. T).

**
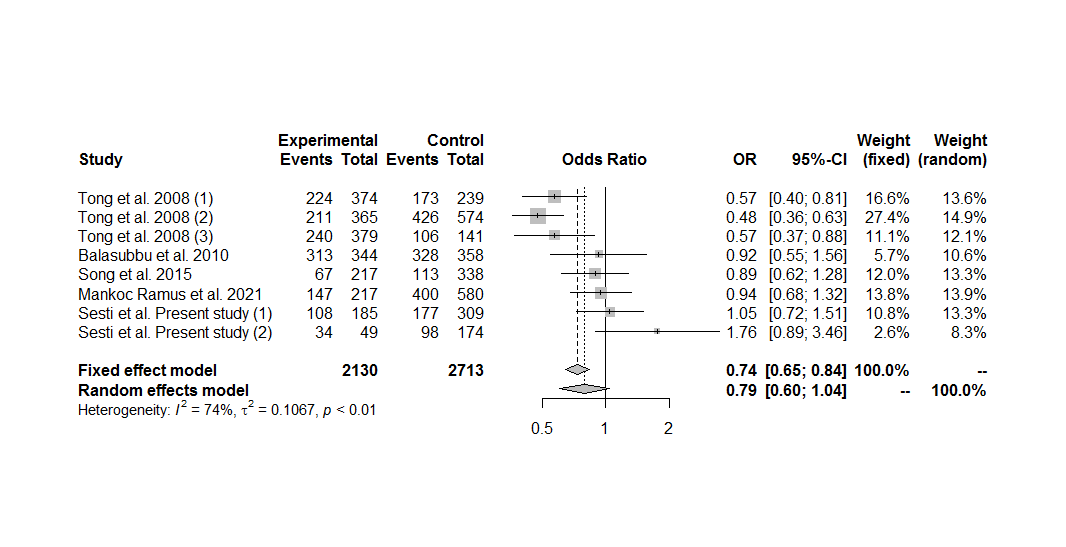
**

**Figure S49.** Forest plot of the association between the *EPO* rs1617640 polymorphism and proliferative diabetic retinopathy, including only the sets with controls in Hardy-Weinberg equilibrium, under the dominant genetic model for the minor allele (GG+TG vs. TT).

**
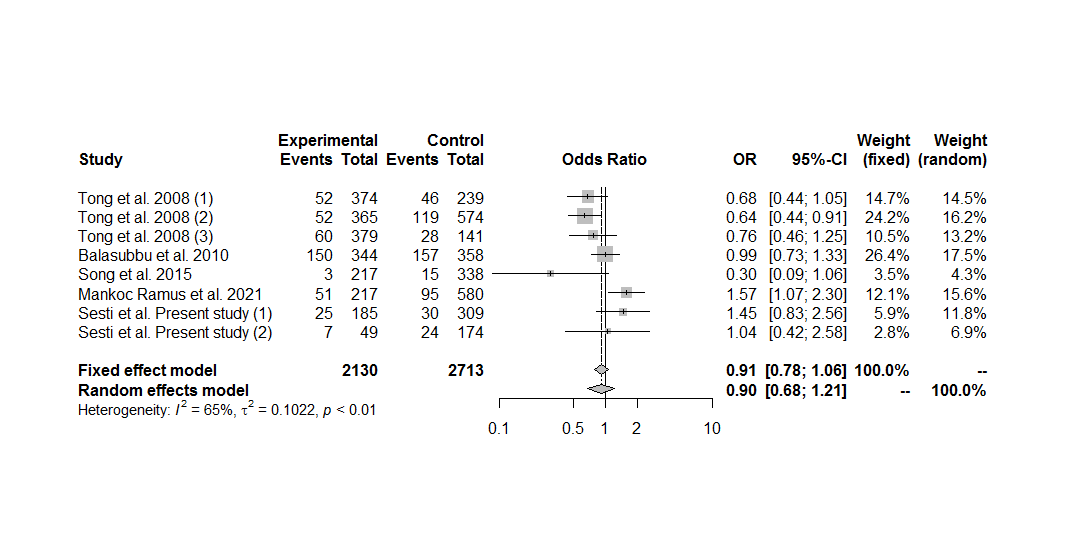
**

**Figure S50.** Forest plot of the association between the *EPO* rs1617640 polymorphism and proliferative diabetic retinopathy, including only the sets with controls in Hardy-Weinberg equilibrium, under the recessive genetic model for the minor allele (GG vs. TG+TT).

**
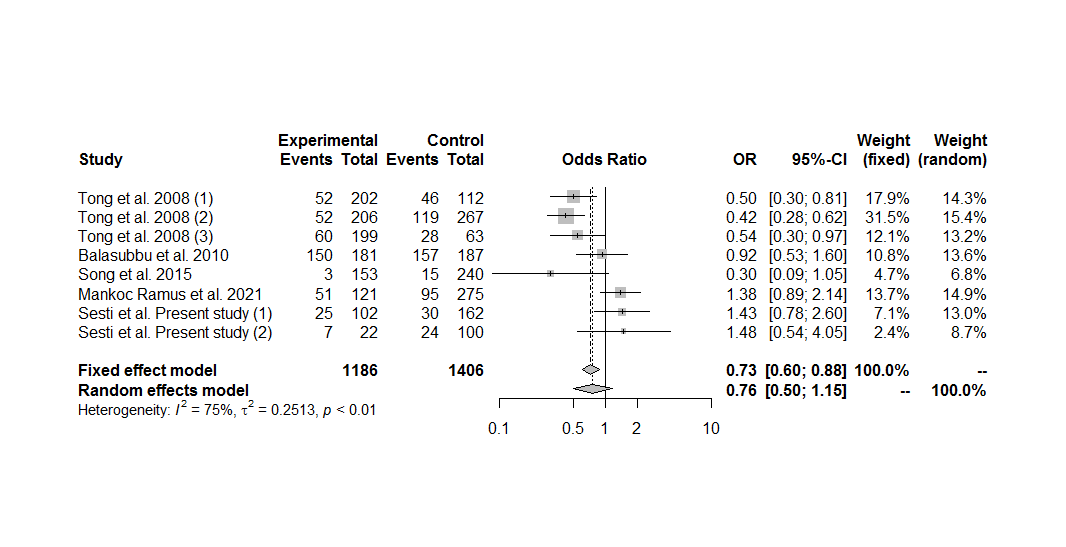
**

**Figure S51.** Forest plot of the association between the *EPO* rs1617640 polymorphism and proliferative diabetic retinopathy, including only the sets with controls in Hardy-Weinberg equilibrium, under the homozygous additive genetic model for the minor allele (GG vs. TT).

**
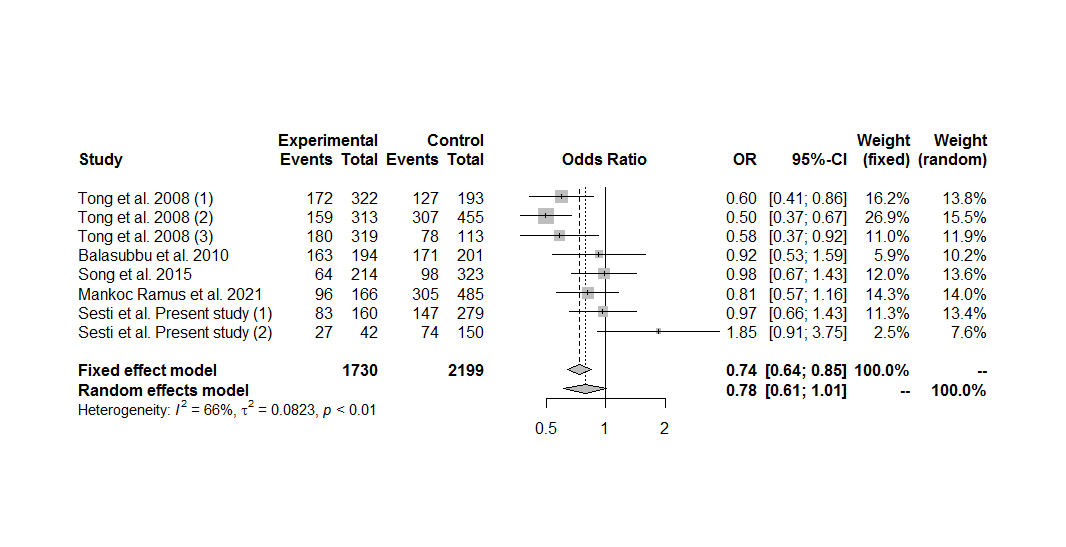
**

**Figure S52.** Forest plot of the association between the *EPO* rs1617640 polymorphism and proliferative diabetic retinopathy, including only the sets with controls in Hardy-Weinberg equilibrium, under the heterozygous additive genetic model for the minor allele (TG vs. TT).

**
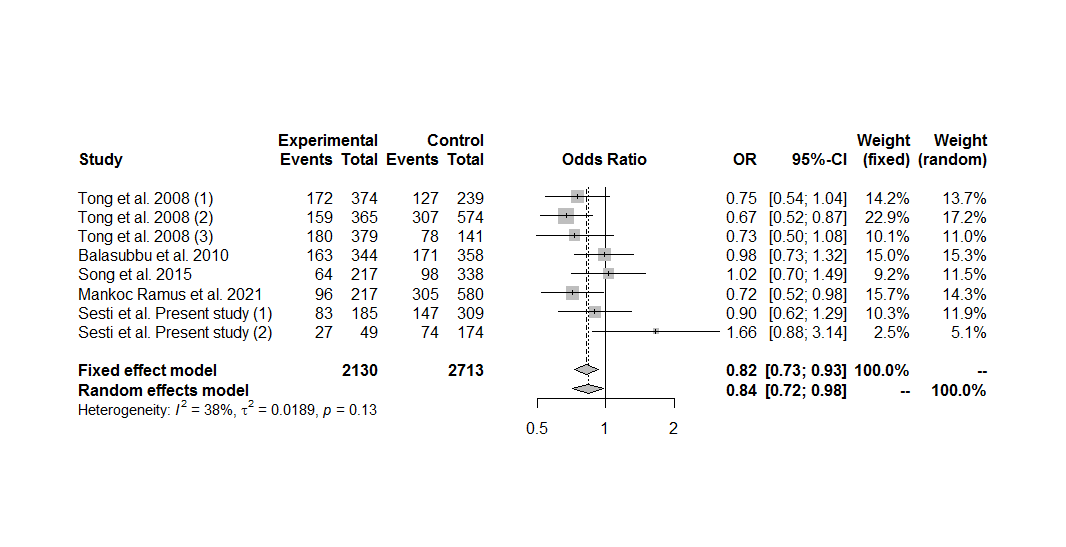
**

**Figure S53.** Forest plot of the association between the *EPO* rs1617640 polymorphism and proliferative diabetic retinopathy, including only the sets with controls in Hardy-Weinberg equilibrium, under the overdominant genetic model (TG vs. GG+TT).

**
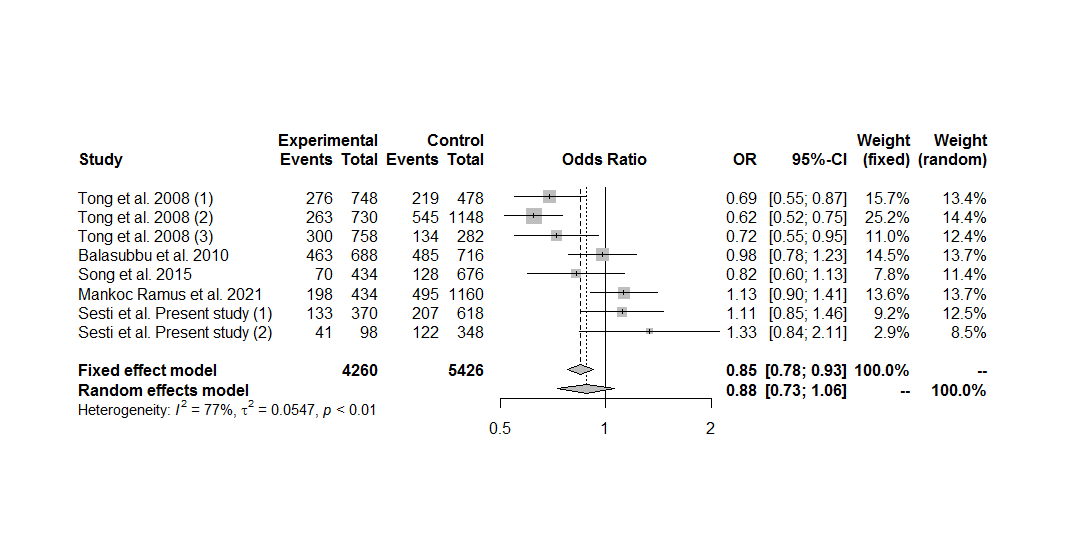
**

**Figure S54.** Forest plot of the association between the *EPO* rs1617640 polymorphism and proliferative diabetic retinopathy, including only the sets with controls in Hardy-Weinberg equilibrium, under the allele contrast genetic model (G vs. T).

**
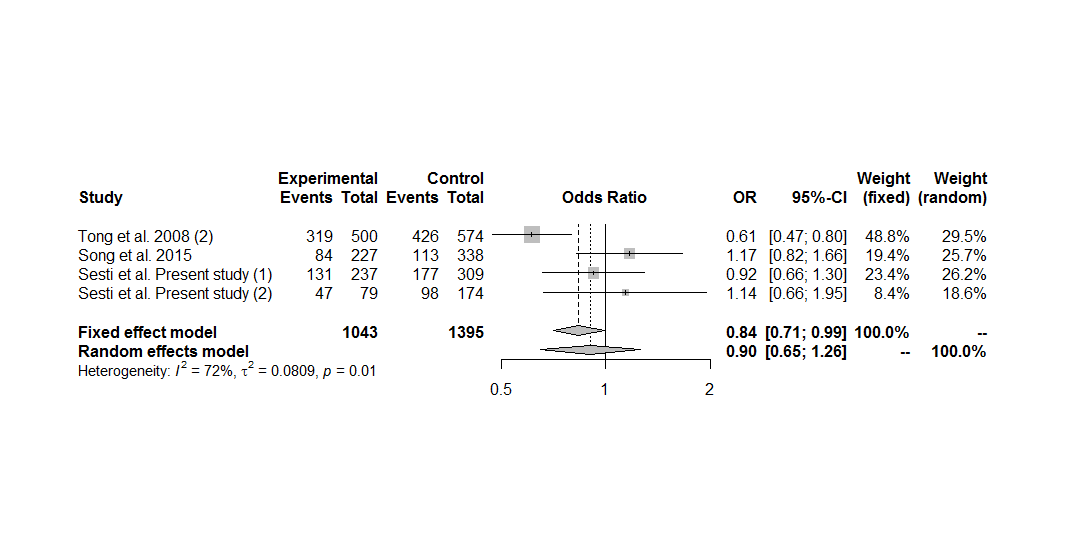
**

**Figure S55.** Forest plot of the association between the *EPO* rs1617640 polymorphism and non-proliferative diabetic retinopathy, including only the sets with controls in Hardy-Weinberg equilibrium, under the dominant genetic model for the minor allele (GG+TG vs. TT).

**
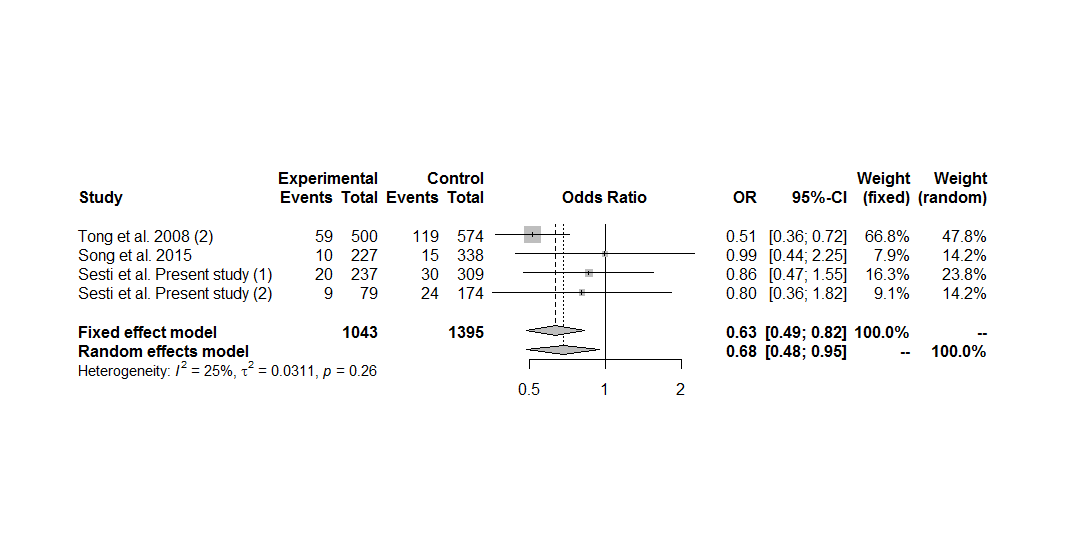
**

**Figure S56.** Forest plot of the association between the *EPO* rs1617640 polymorphism and non-proliferative diabetic retinopathy, including only the sets with controls in Hardy-Weinberg equilibrium, under the recessive genetic model for the minor allele (GG vs. TG+TT).

**
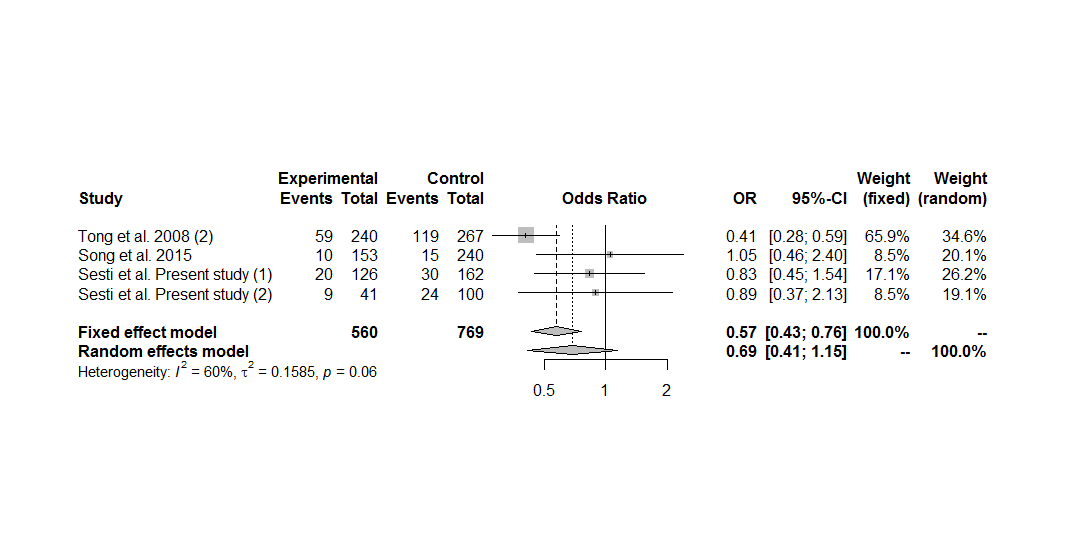
**

**Figure S57.** Forest plot of the association between the *EPO* rs1617640 polymorphism and non-proliferative diabetic retinopathy, including only the sets with controls in Hardy-Weinberg equilibrium, under the homozygous additive genetic model for the minor allele (GG vs. TT).

**
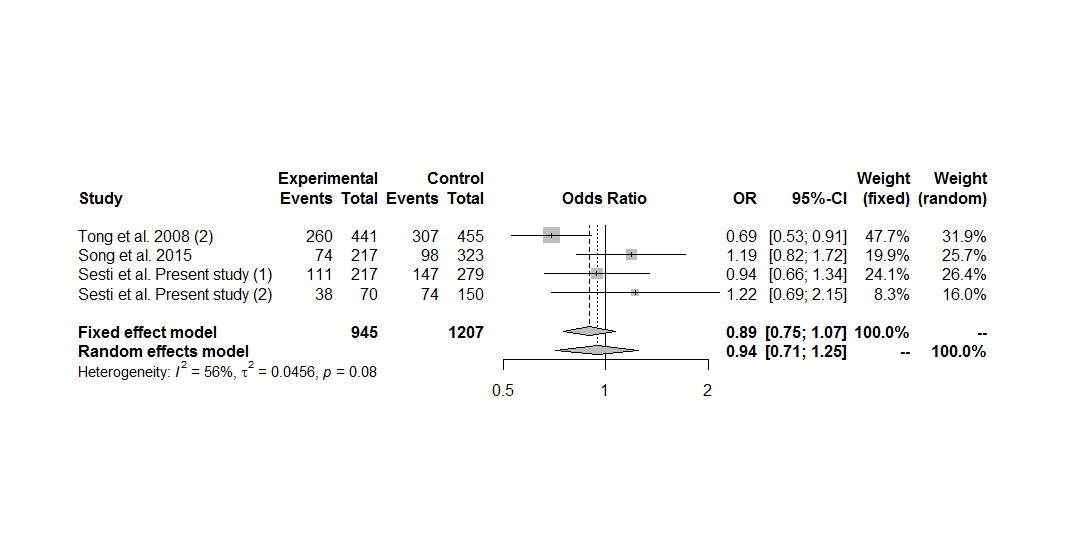
**

**Figure S58.** Forest plot of the association between the *EPO* rs1617640 polymorphism and non-proliferative diabetic retinopathy, including only the sets with controls in Hardy-Weinberg equilibrium, under the heterozygous additive genetic model for the minor allele (TG vs. TT).

**
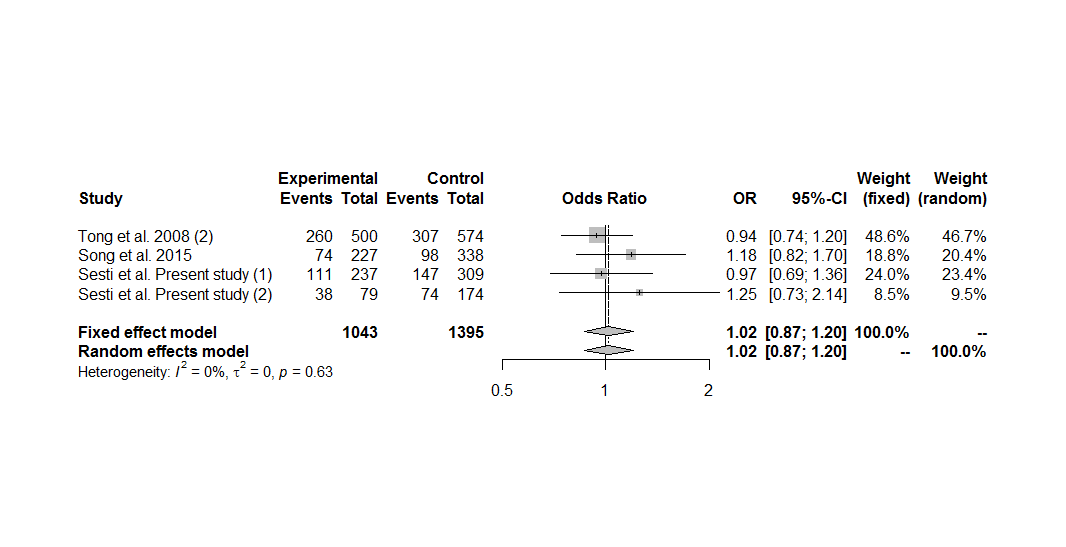
**

**Figure S59.** Forest plot of the association between the *EPO* rs1617640 polymorphism and non-proliferative diabetic retinopathy, including only the sets with controls in Hardy-Weinberg equilibrium, under the overdominant genetic model (TG vs. GG+TT).

**
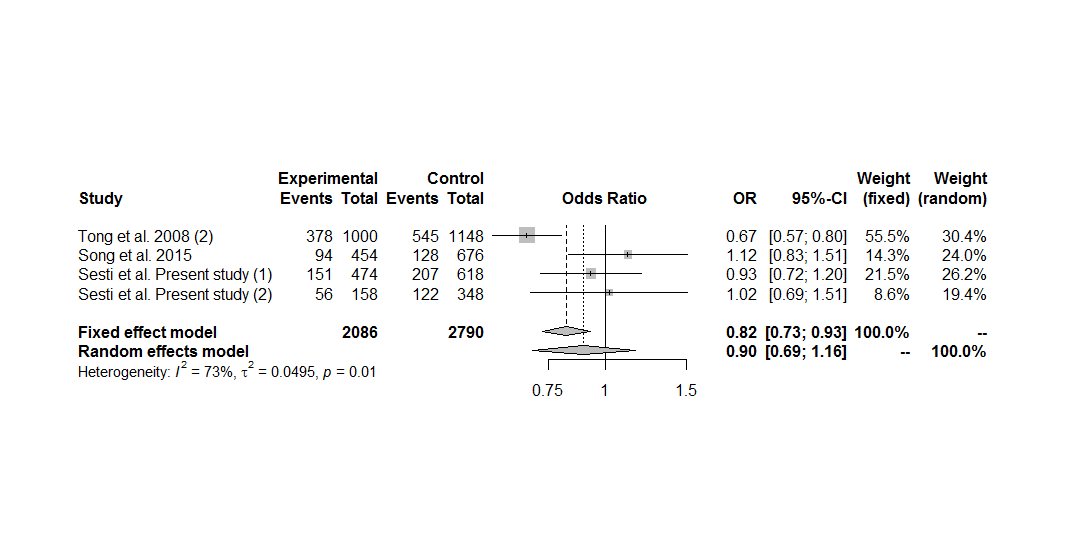
**

**Figure S60.** Forest plot of the association between the *EPO* rs1617640 polymorphism and non-proliferative diabetic retinopathy, including only the sets with controls in Hardy-Weinberg equilibrium, under the allele contrast genetic model (G vs. T).

**
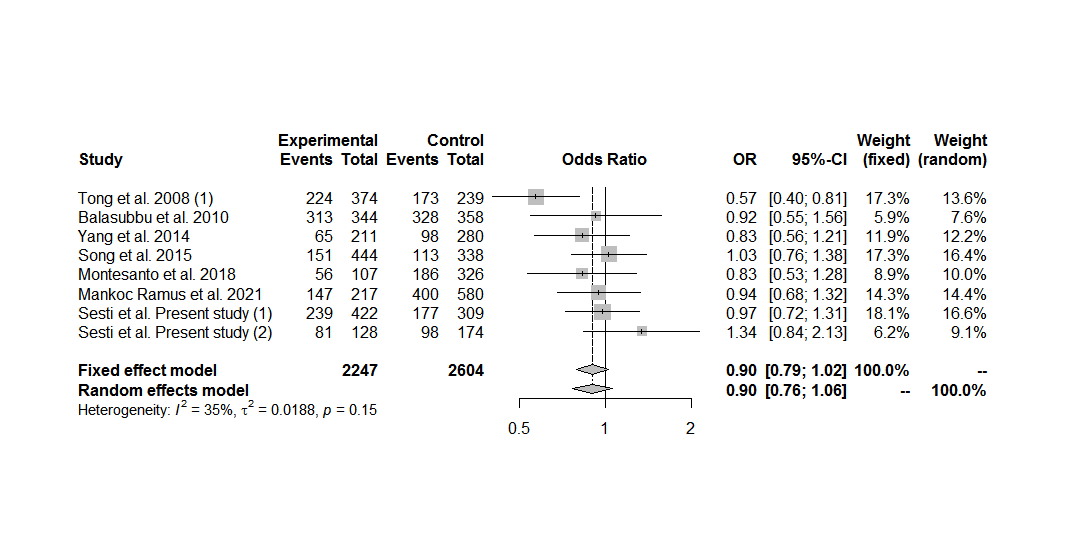
**

**Figure S61.** Forest plot of the association between the *EPO* rs1617640 polymorphism and diabetic retinopathy in subjects with type 2 diabetes, including only the sets with controls in Hardy-Weinberg equilibrium, under the dominant genetic model for the minor allele (GG+TG vs. TT).

**
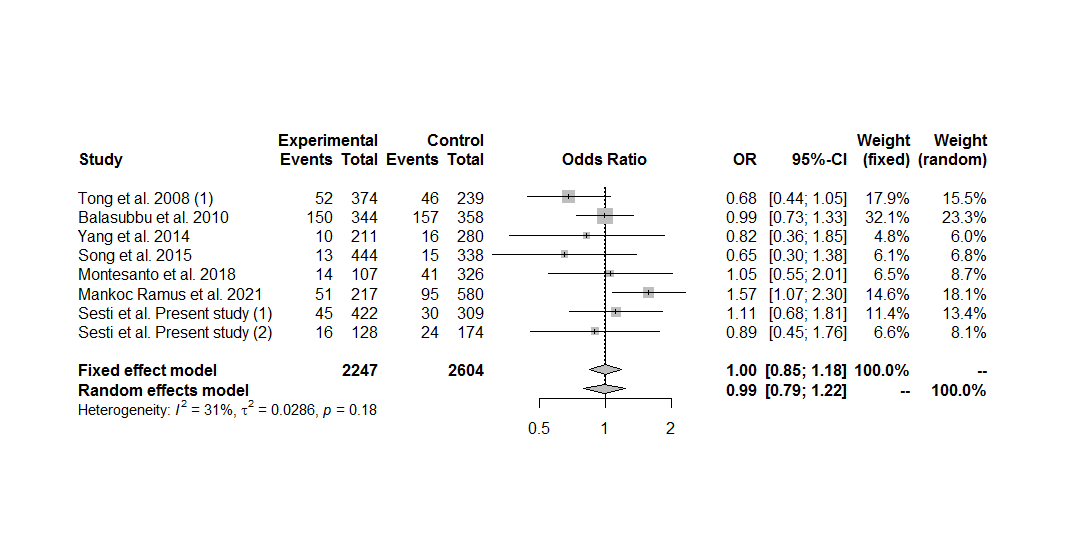
**

**Figure S62.** Forest plot of the association between the *EPO* rs1617640 polymorphism and diabetic retinopathy in subjects with type 2 diabetes, including only the sets with controls in Hardy-Weinberg equilibrium, under the recessive genetic model for the minor allele (GG vs. TG+TT).

**
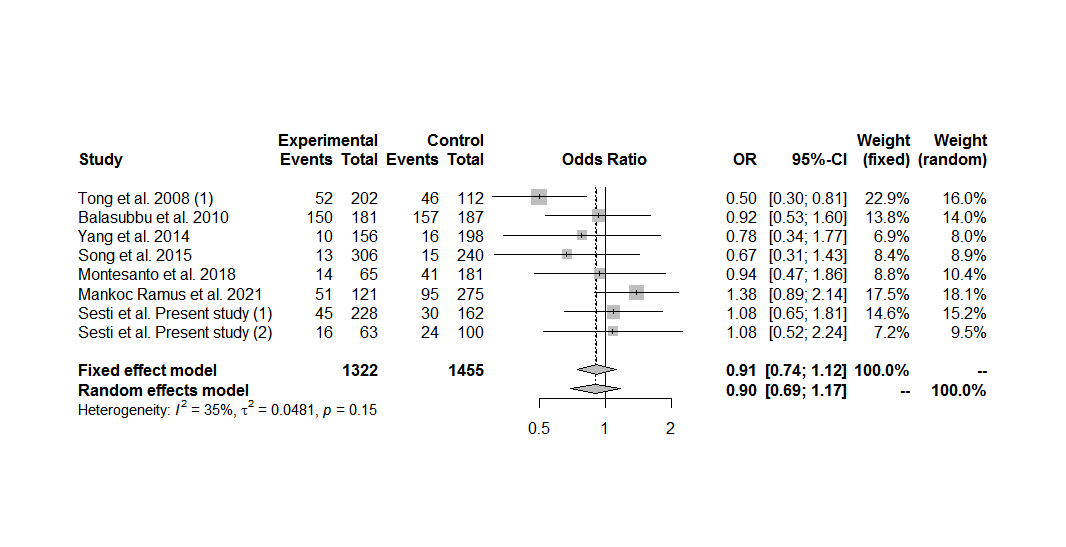
**

**Figure S63.** Forest plot of the association between the *EPO* rs1617640 polymorphism and diabetic retinopathy in subjects with type 2 diabetes, including only the sets with controls in Hardy-Weinberg equilibrium, under the homozygous additive genetic model for the minor allele (GG vs. TT).

**
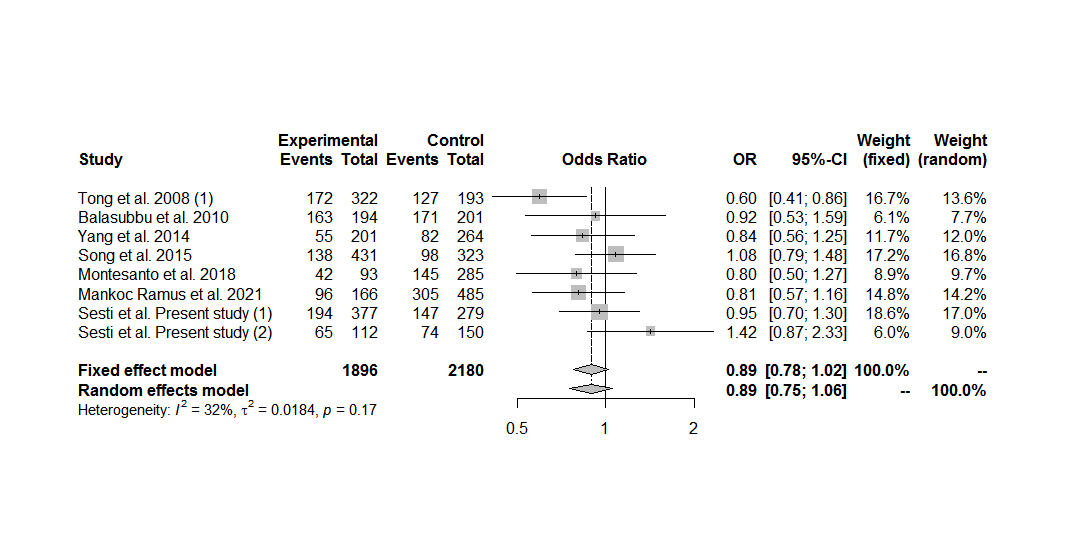
**

**Figure S64.** Forest plot of the association between the *EPO* rs1617640 polymorphism and diabetic retinopathy in subjects with type 2 diabetes, including only the sets with controls in Hardy-Weinberg equilibrium, under the heterozygous additive genetic model for the minor allele (TG vs. TT).

**
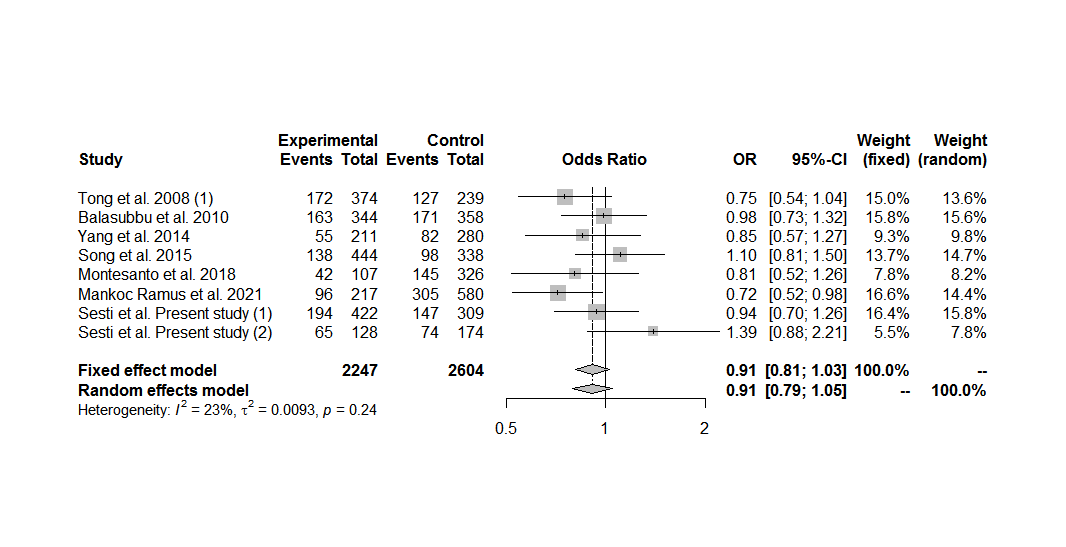
**

**Figure S65.** Forest plot of the association between the *EPO* rs1617640 polymorphism and diabetic retinopathy in subjects with type 2 diabetes, including only the sets with controls in Hardy-Weinberg equilibrium, under the overdominant genetic model (TG vs. GG+TT).

**
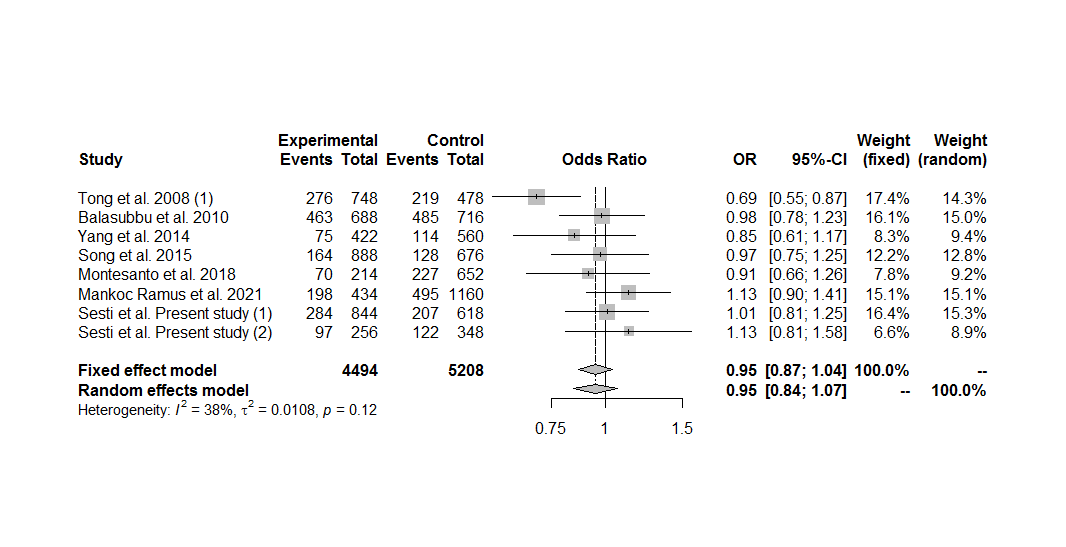
**

**Figure S66.** Forest plot of the association between the *EPO* rs1617640 polymorphism and diabetic retinopathy in subjects with type 2 diabetes, including only the sets with controls in Hardy-Weinberg equilibrium, under the allele contrast genetic model (G vs. T).

**
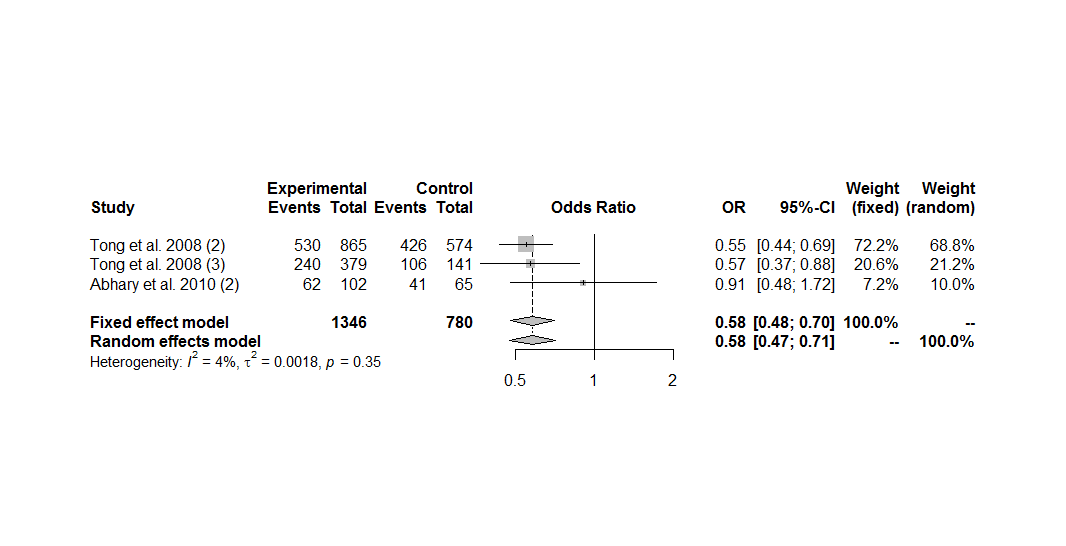
**

**Figure S67.** Forest plot of the association between the *EPO* rs1617640 polymorphism and diabetic retinopathy in subjects with type 1 diabetes, including only the sets with controls in Hardy-Weinberg equilibrium, under the dominant genetic model for the minor allele (GG+TG vs. TT).

**
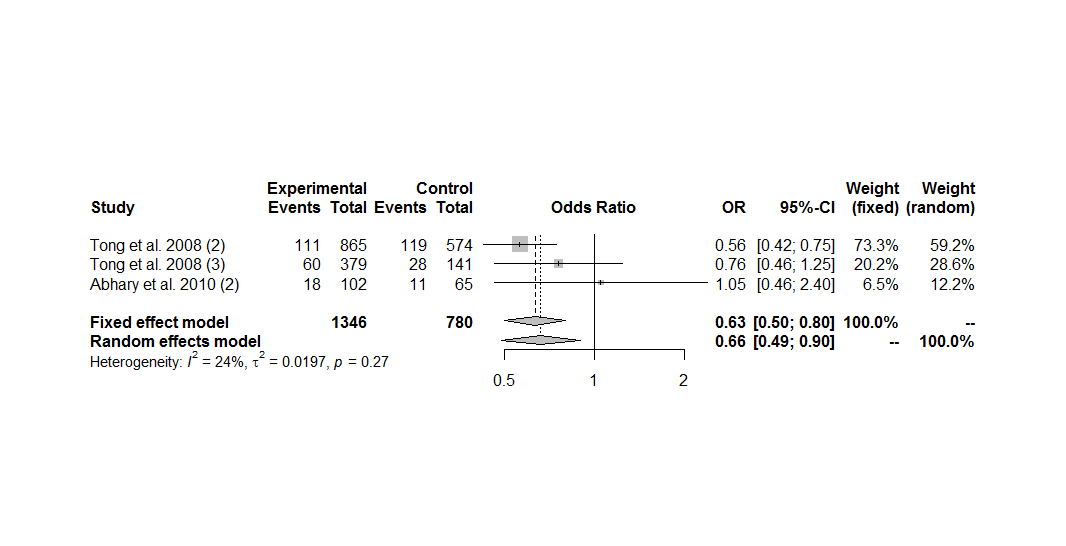
**

**Figure S68.** Forest plot of the association between the *EPO* rs1617640 polymorphism and diabetic retinopathy in subjects with type 1 diabetes, including only the sets with controls in Hardy-Weinberg equilibrium, under the recessive genetic model for the minor allele (GG vs. TG+TT).

**
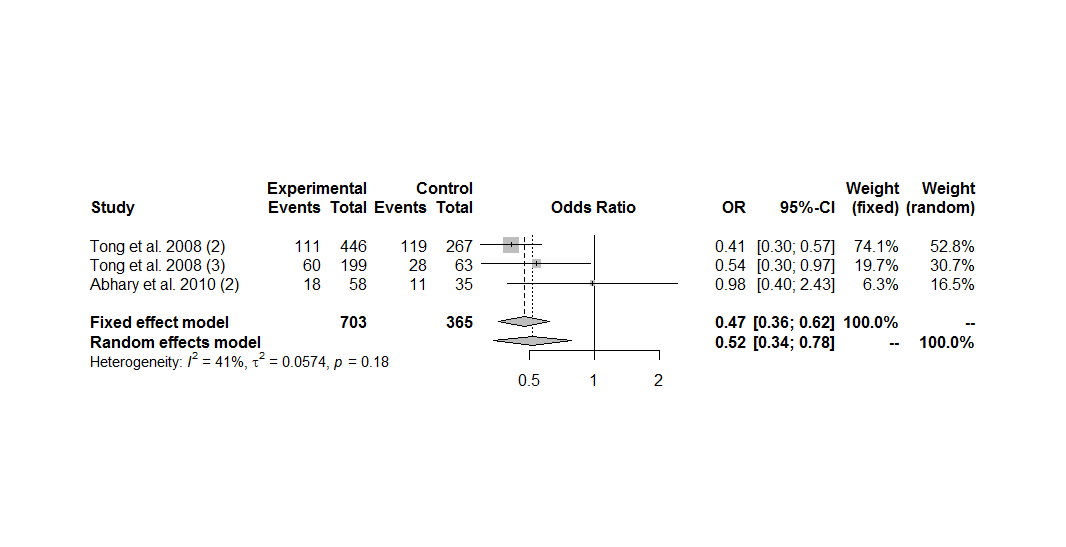
**

**Figure S69.** Forest plot of the association between the *EPO* rs1617640 polymorphism and diabetic retinopathy in subjects with type 1 diabetes, including only the sets with controls in Hardy-Weinberg equilibrium, under the homozygous additive genetic model for the minor allele (GG vs. TT).

**
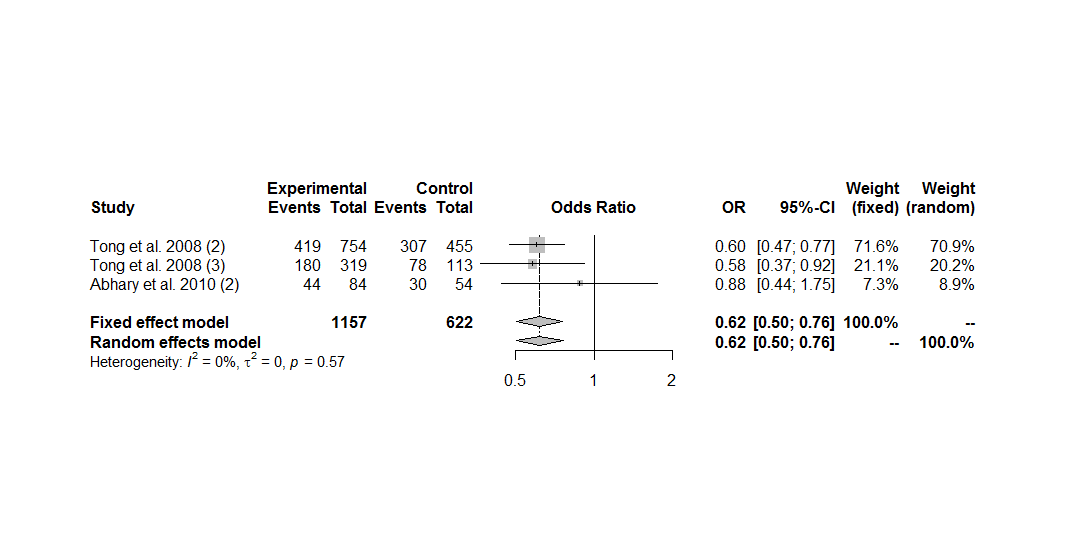
**

**Figure S70.** Forest plot of the association between the *EPO* rs1617640 polymorphism and diabetic retinopathy in subjects with type 1 diabetes, including only the sets with controls in Hardy-Weinberg equilibrium, under the heterozygous additive genetic model for the minor allele (TG vs. TT).

**
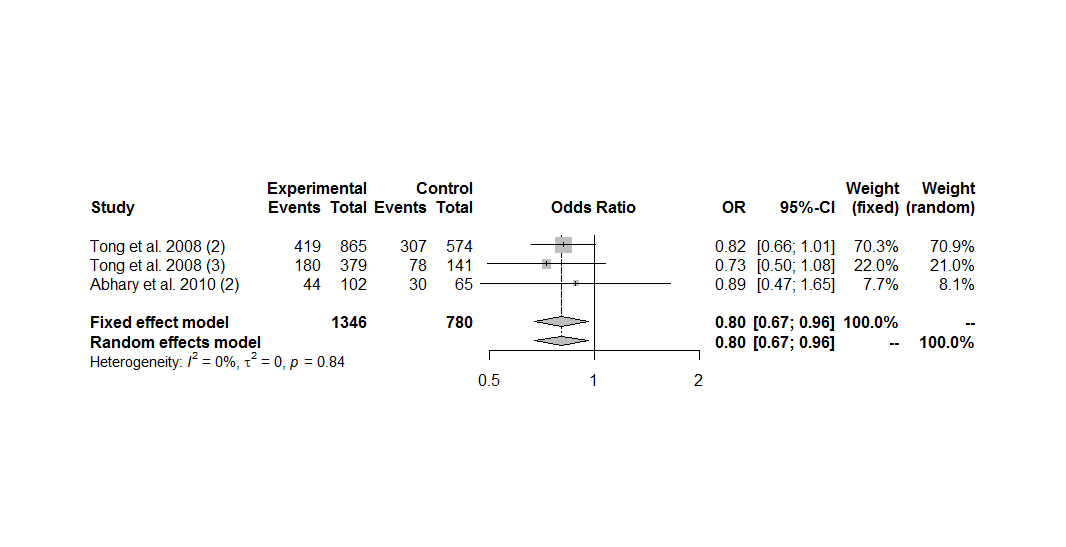
**

**Figure S71.** Forest plot of the association between the *EPO* rs1617640 polymorphism and diabetic retinopathy in subjects with type 1 diabetes, including only the sets with controls in Hardy-Weinberg equilibrium, under the overdominant genetic model (TG vs. GG+TT).

**
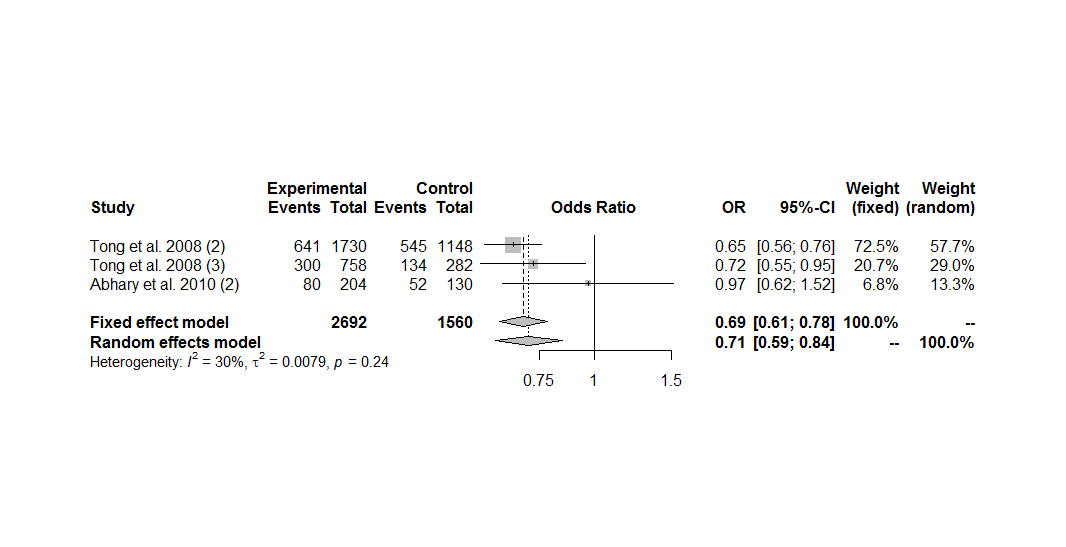
**

**Figure S72.** Forest plot of the association between the *EPO* rs1617640 polymorphism and diabetic retinopathy in subjects with type 1 diabetes, including only the sets with controls in Hardy-Weinberg equilibrium, under the allele contrast genetic model (G vs. T).

**
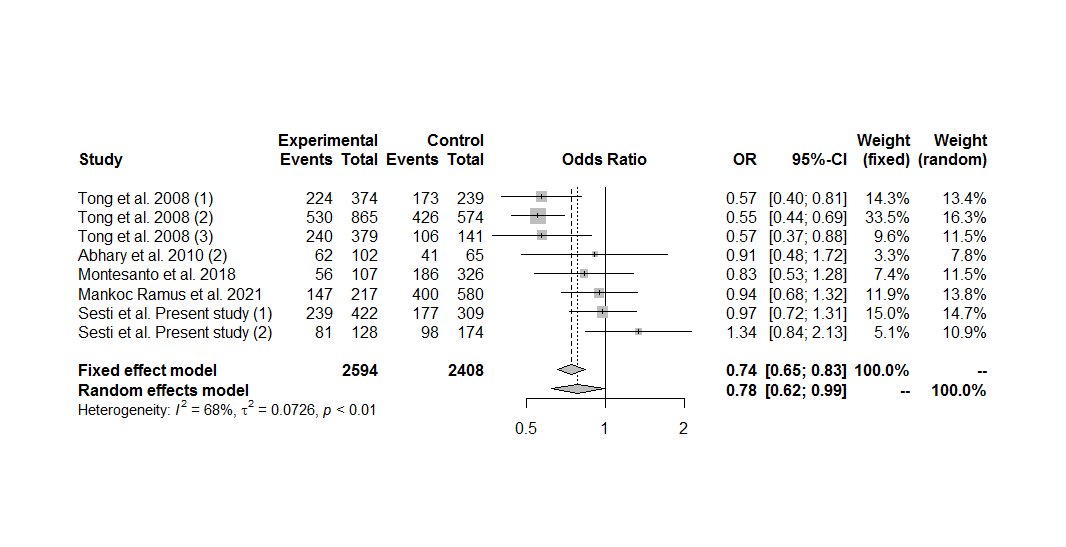
**

**Figure S73.** Forest plot of the association between the *EPO* rs1617640 polymorphism and diabetic retinopathy in non-Asians, including only the sets with controls in Hardy-Weinberg equilibrium, under the dominant genetic model for the minor allele (GG+TG vs. TT).

**
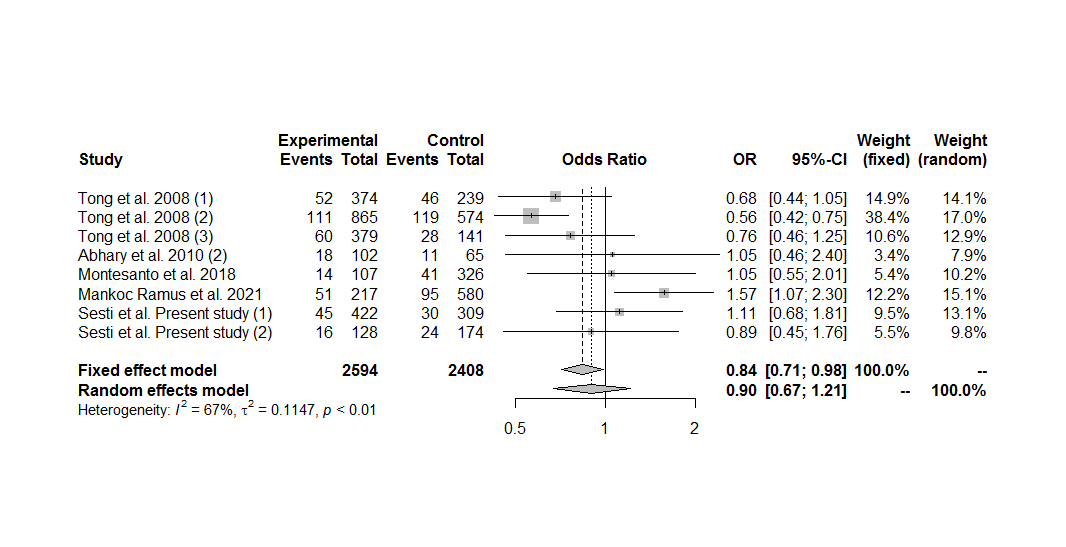
**

**Figure S74.** Forest plot of the association between the *EPO* rs1617640 polymorphism and diabetic retinopathy in non-Asians, including only the sets with controls in Hardy-Weinberg equilibrium, under the recessive genetic model for the minor allele (GG vs. TG+TT).

**
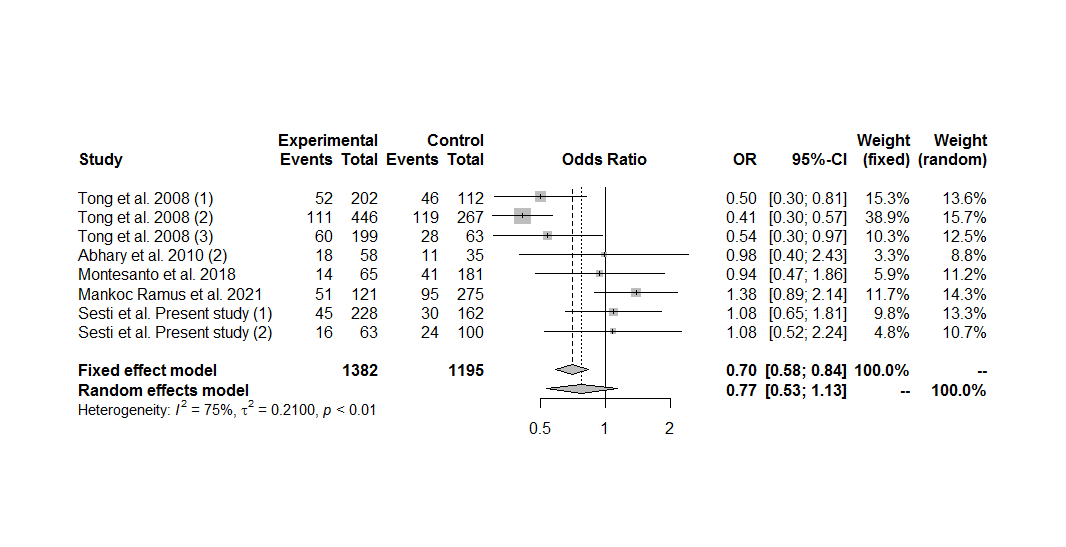
**

**Figure S75.** Forest plot of the association between the *EPO* rs1617640 polymorphism and diabetic retinopathy in non-Asians, including only the sets with controls in Hardy-Weinberg equilibrium, under the homozygous additive genetic model for the minor allele (GG vs. TT).

**
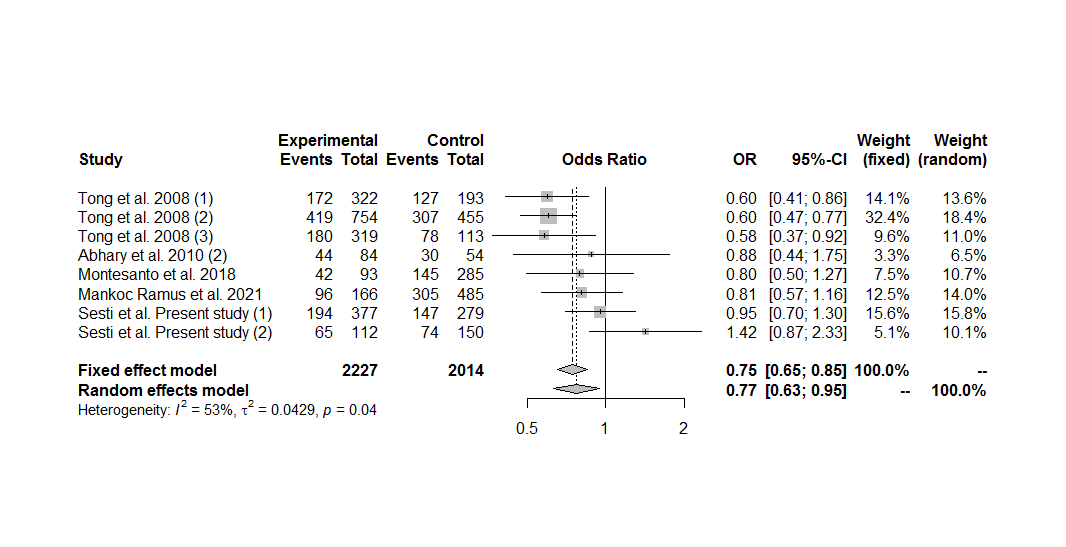
**

**Figure S76.** Forest plot of the association between the *EPO* rs1617640 polymorphism and diabetic retinopathy in non-Asians, including only the sets with controls in Hardy-Weinberg equilibrium, under the heterozygous additive genetic model for the minor allele (TG vs. TT).

**
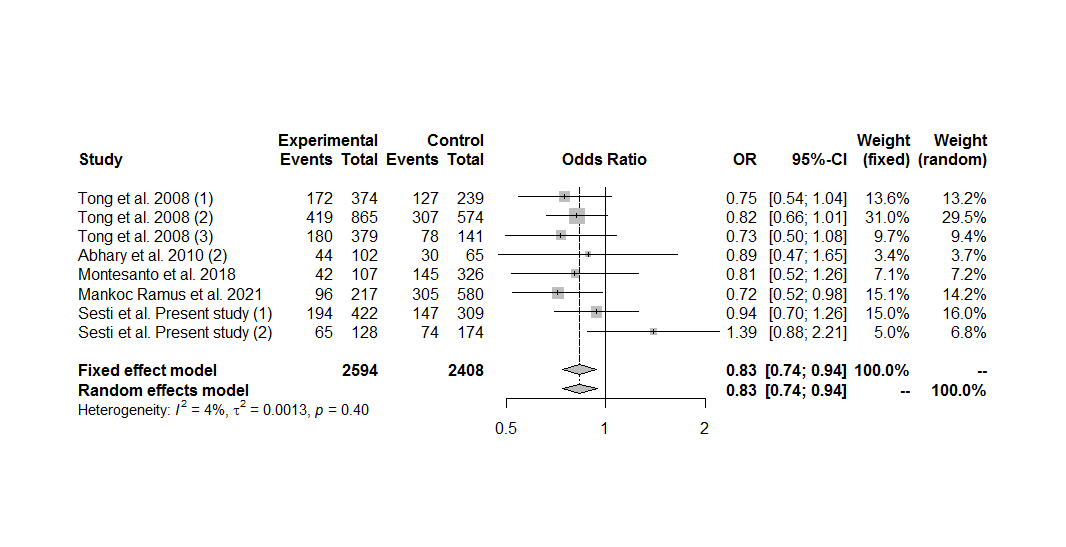
**

**Figure S77.** Forest plot of the association between the *EPO* rs1617640 polymorphism and diabetic retinopathy in non-Asians, including only the sets with controls in Hardy-Weinberg equilibrium, under the overdominant genetic model (TG vs. GG+TT).

**
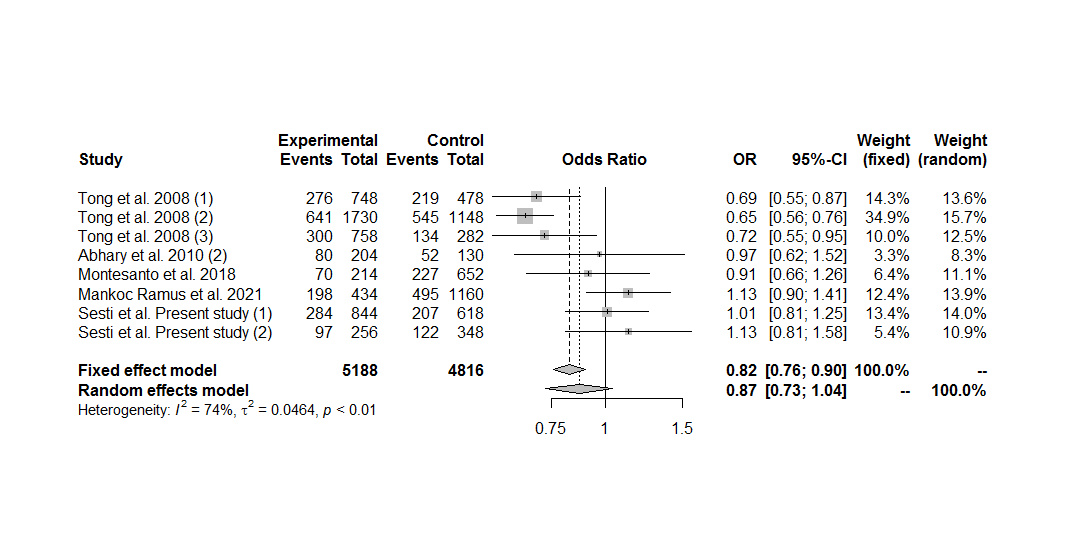
**

**Figure S78.** Forest plot of the association between the *EPO* rs1617640 polymorphism and diabetic retinopathy in non-Asians, including only the sets with controls in Hardy-Weinberg equilibrium, under the allele contrast genetic model (G vs. T).

**
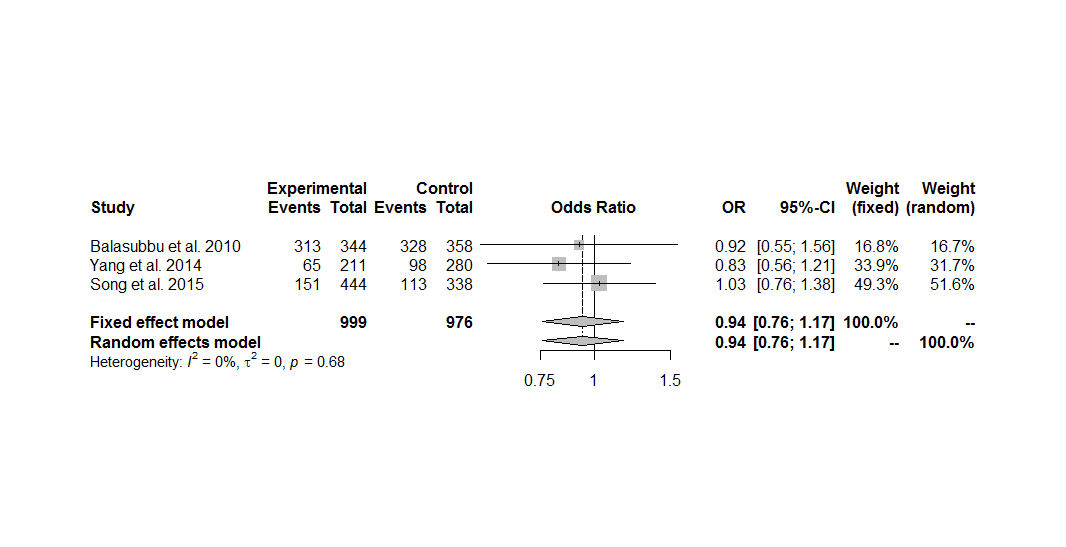
**

**Figure S79.** Forest plot of the association between the *EPO* rs1617640 polymorphism and diabetic retinopathy in Asians, including only the sets with controls in Hardy-Weinberg equilibrium, under the dominant genetic model for the minor allele (GG+TG vs. TT).

**
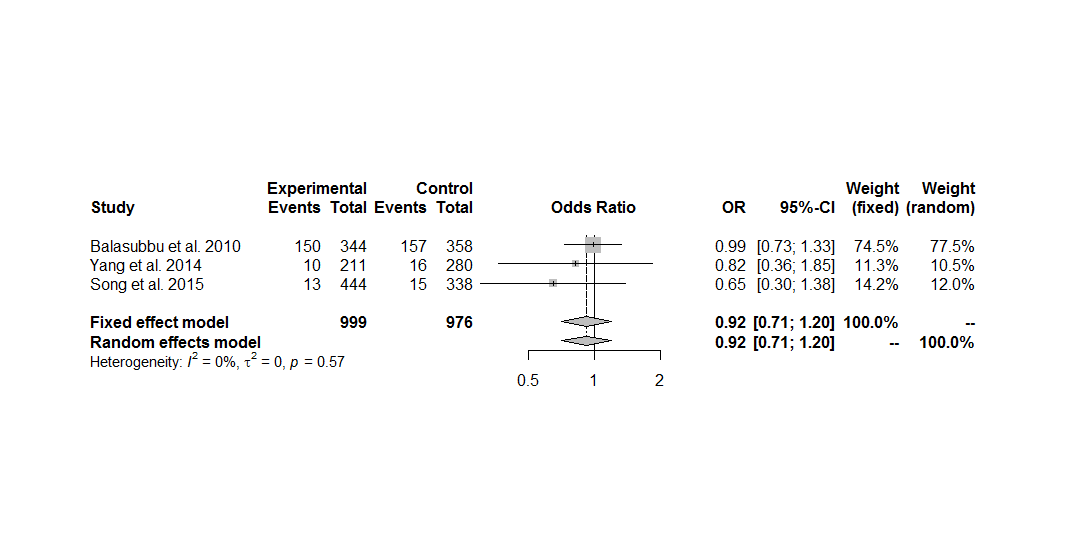
**

**Figure S80.** Forest plot of the association between the *EPO* rs1617640 polymorphism and diabetic retinopathy in Asians, including only the sets with controls in Hardy-Weinberg equilibrium, under the recessive genetic model for the minor allele (GG vs. TG+TT).

**
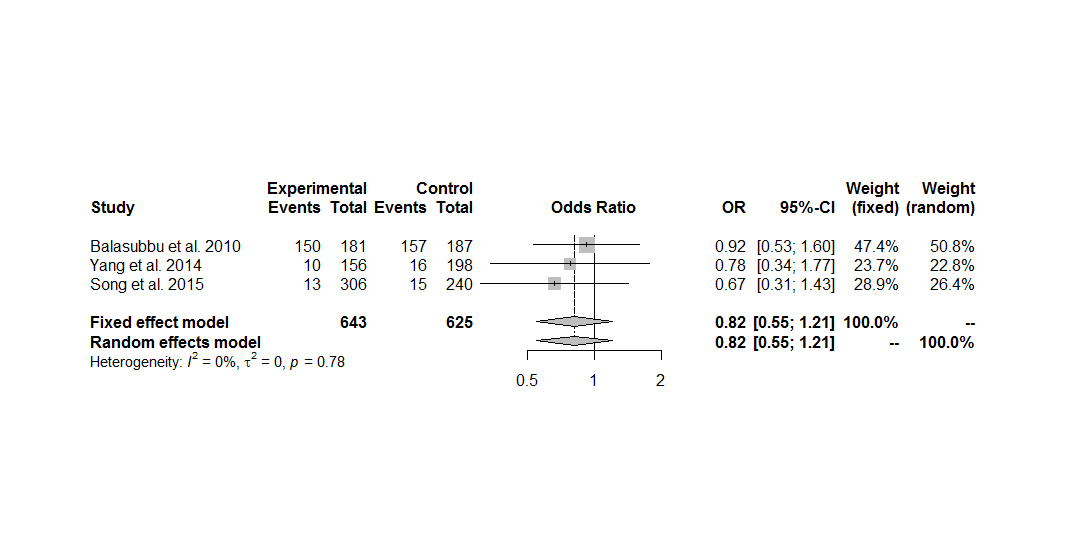
**

**Figure S81.** Forest plot of the association between the *EPO* rs1617640 polymorphism and diabetic retinopathy in Asians, including only the sets with controls in Hardy-Weinberg equilibrium, under the homozygous additive genetic model for the minor allele (GG vs. TT).

**
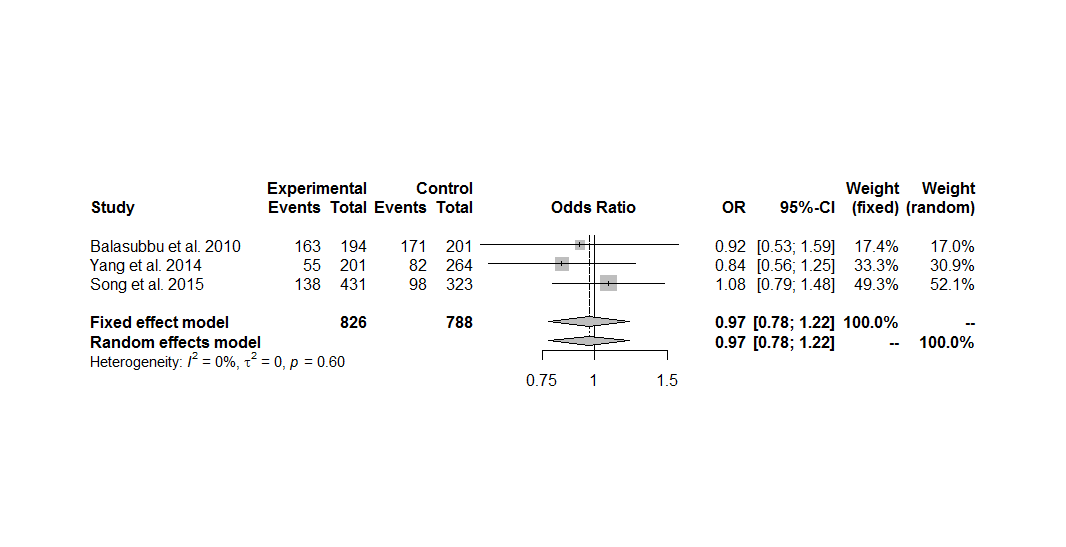
**

**Figure S82.** Forest plot of the association between the *EPO* rs1617640 polymorphism and diabetic retinopathy in Asians, including only the sets with controls in Hardy-Weinberg equilibrium, under the heterozygous additive genetic model for the minor allele (TG vs. TT).

**
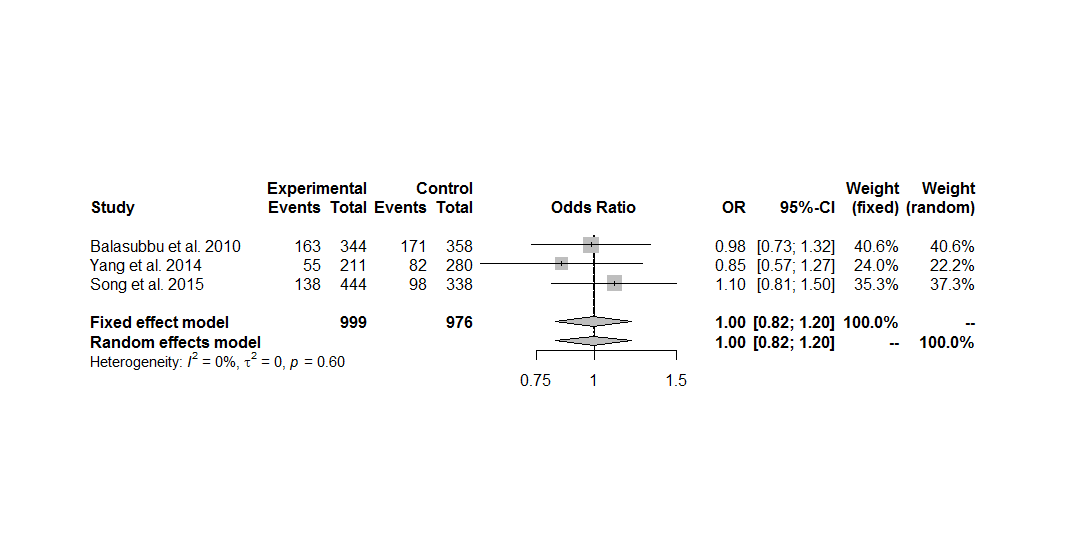
**

**Figure S83.** Forest plot of the association between the *EPO* rs1617640 polymorphism and diabetic retinopathy in Asians, including only the sets with controls in Hardy-Weinberg equilibrium, under the overdominant genetic model (TG vs. GG+TT).

**
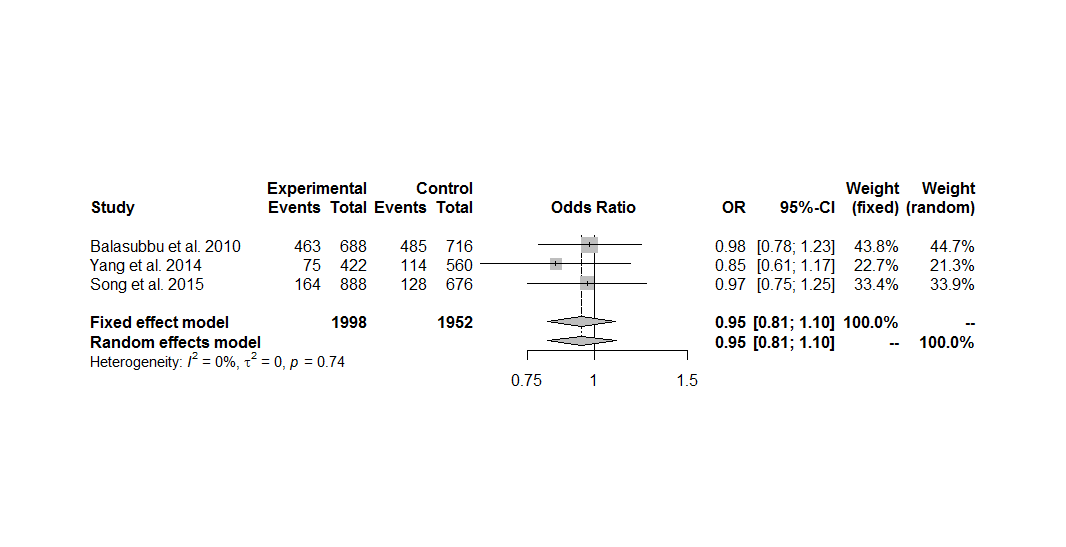
**

**Figure S84.** Forest plot of the association between the *EPO* rs1617640 polymorphism and diabetic retinopathy in Asians, including only the sets with controls in Hardy-Weinberg equilibrium, under the allele contrast genetic model (G vs. T).

**
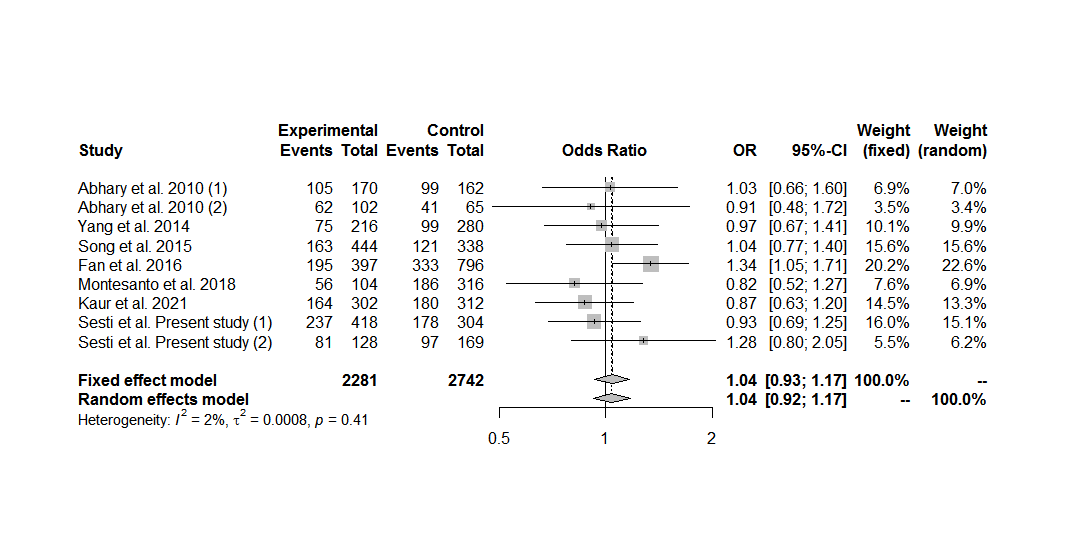
**

**Figure S85.** Forest plot of the association between the *EPO* rs507392 polymorphism and diabetic retinopathy in the overall group analysis, under the dominant genetic model for the minor allele (CC+TC vs. TT).

**
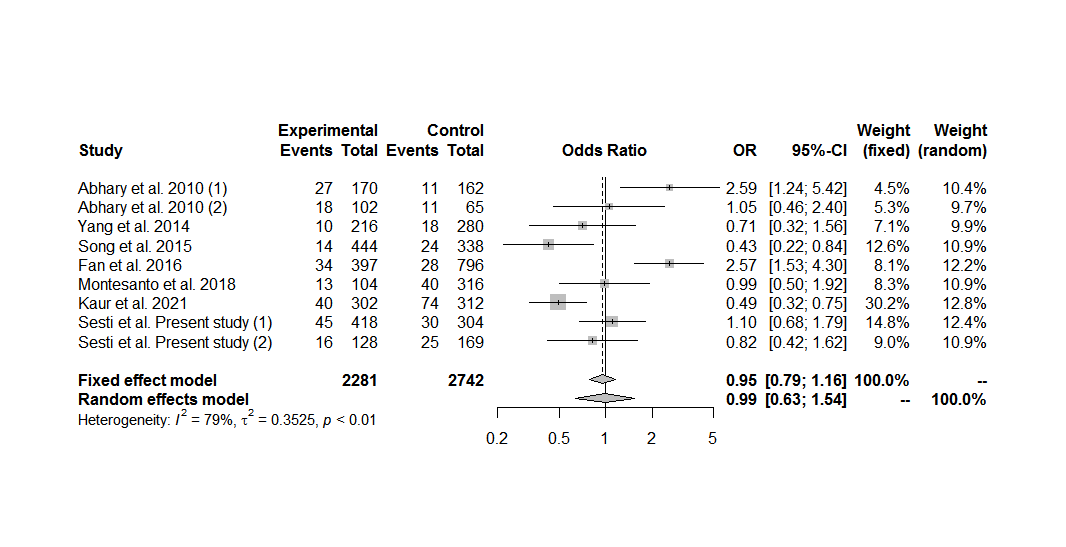
**

**Figure S86.** Forest plot of the association between the *EPO* rs507392 polymorphism and diabetic retinopathy in the overall group analysis, under the recessive genetic model for the minor allele (CC vs. TC+TT).

**
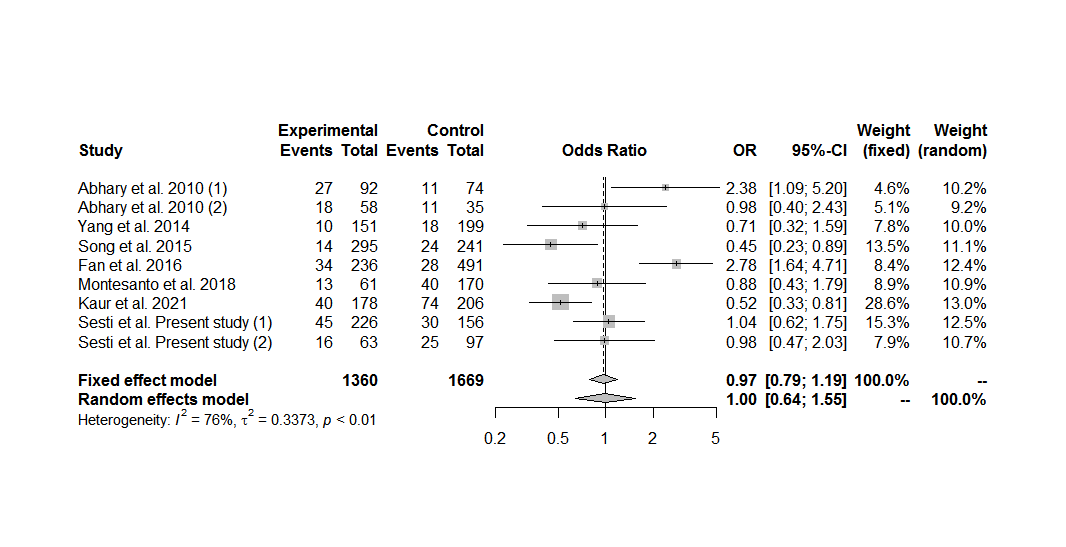
**

**Figure S87.** Forest plot of the association between the *EPO* rs507392 polymorphism and diabetic retinopathy in the overall group analysis, under the homozygous additive genetic model for the minor allele (CC vs. TT).

**
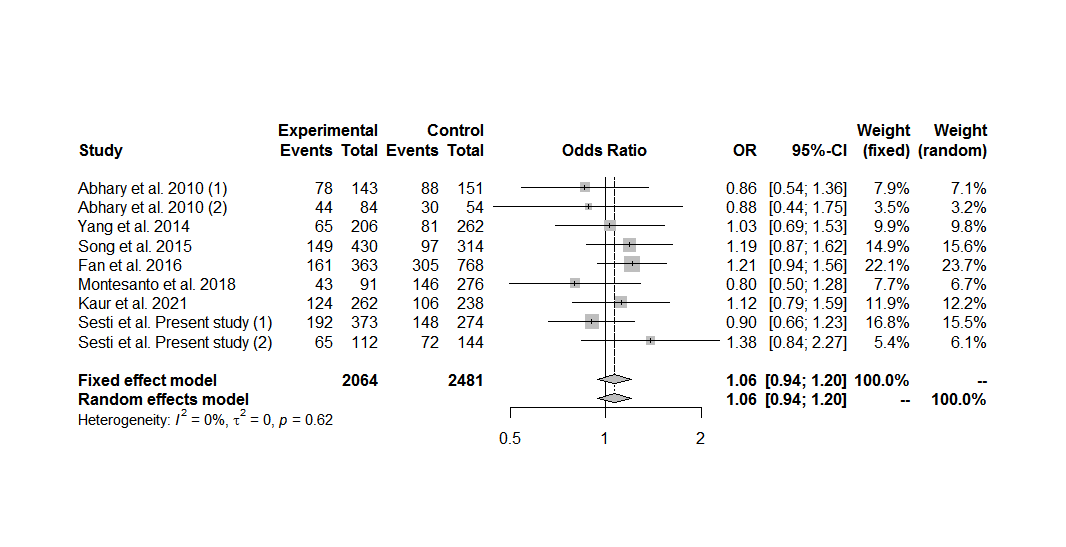
**

**Figure S88.** Forest plot of the association between the *EPO* rs507392 polymorphism and diabetic retinopathy in the overall group analysis, under the heterozygous additive genetic model for the minor allele (TC vs. TT).

**
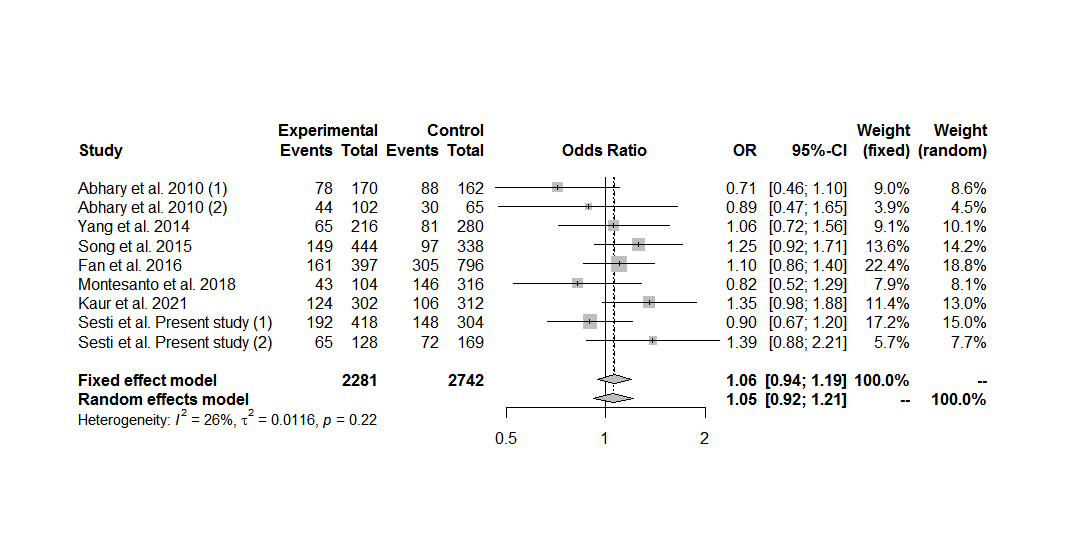
**

**Figure S89.** Forest plot of the association between the *EPO* rs507392 polymorphism and diabetic retinopathy in the overall group analysis, under the overdominant genetic model (TC vs. CC+TT).

**
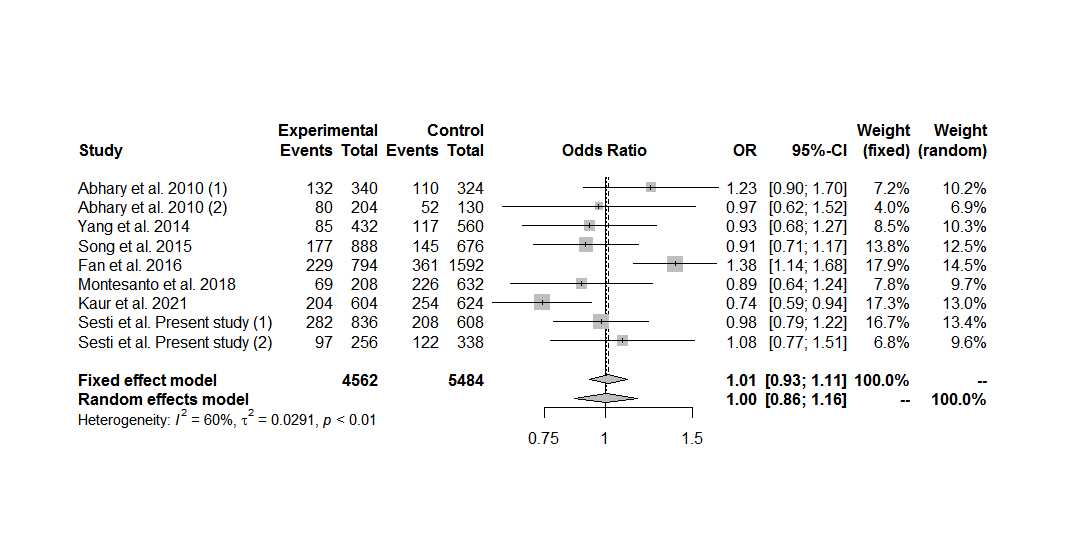
**

**Figure S90.** Forest plot of the association between the *EPO* rs507392 polymorphism and diabetic retinopathy in the overall group analysis, under the allele contrast genetic model (C vs. T).

**
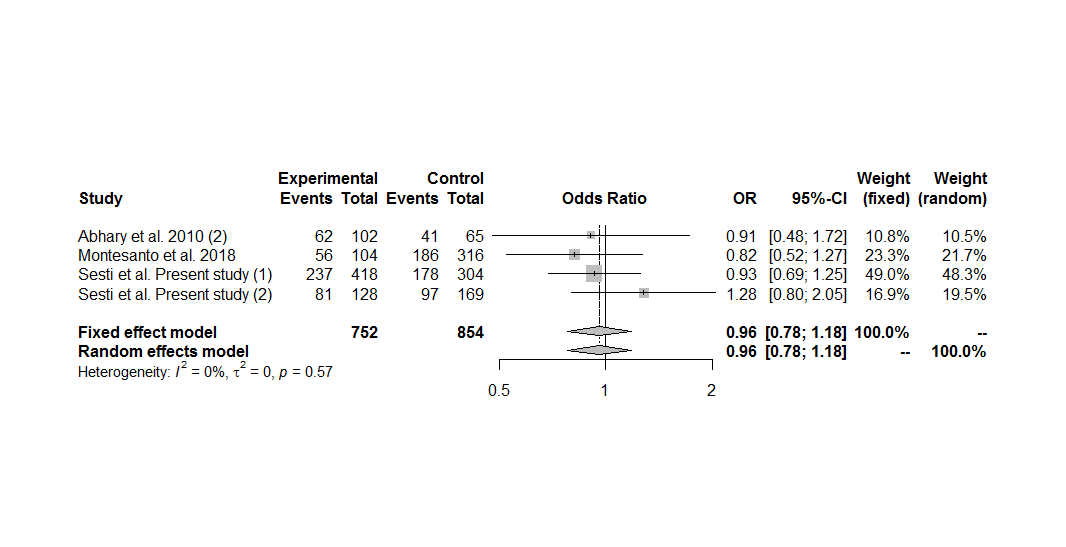
**

**Figure S91.** Forest plot of the association between the *EPO* rs507392 polymorphism and diabetic retinopathy in the overall group analysis including only the sets with controls in Hardy-Weinberg equilibrium, under the dominant genetic model for the minor allele (CC+TC vs. TT).

**
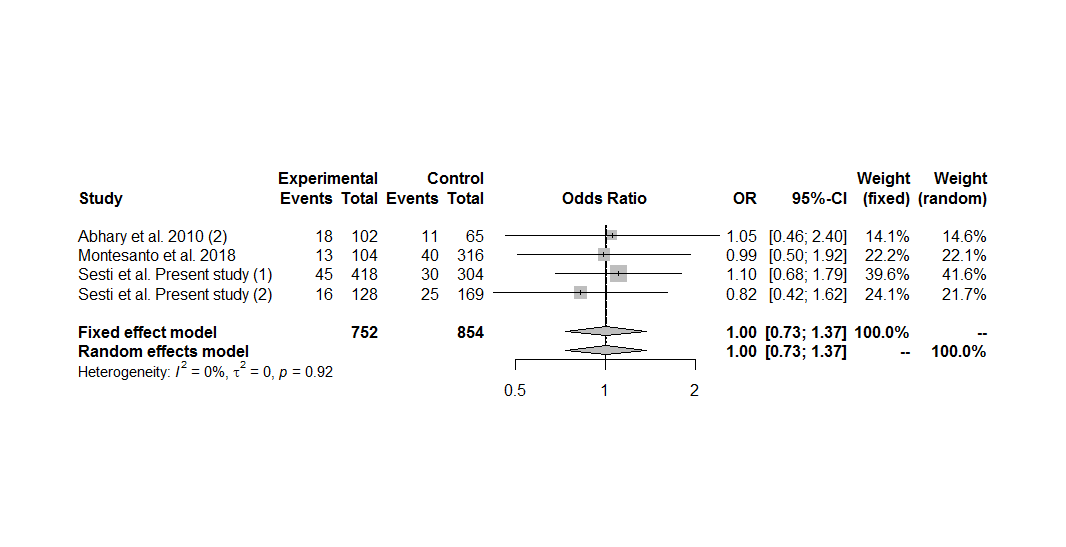
**

**Figure S92.** Forest plot of the association between the *EPO* rs507392 polymorphism and diabetic retinopathy in the overall group analysis including only the sets with controls in Hardy-Weinberg equilibrium, under the recessive genetic model for the minor allele (CC vs. TC+TT).

**
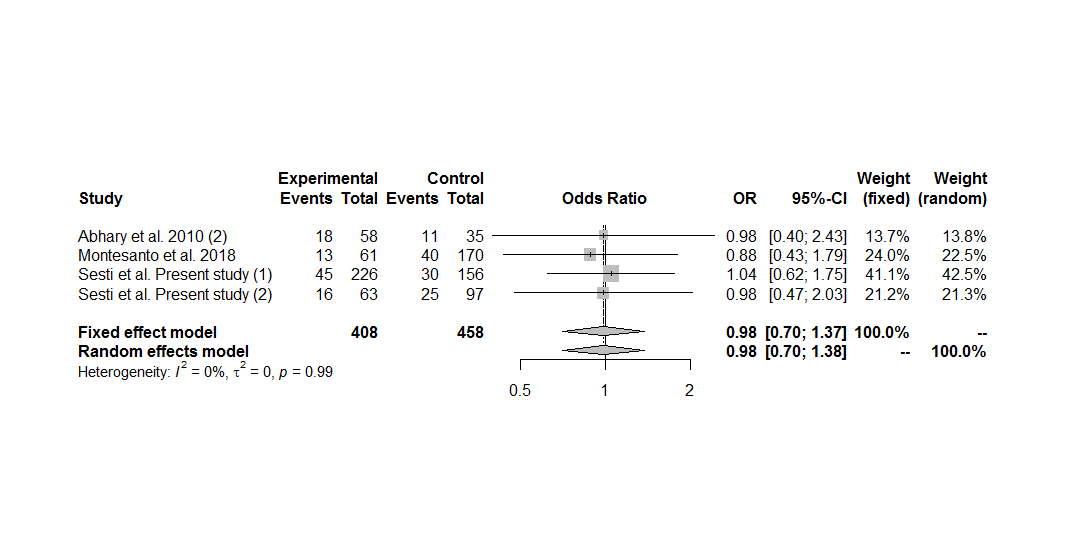
**

**Figure S93.** Forest plot of the association between the *EPO* rs507392 polymorphism and diabetic retinopathy in the overall group analysis including only the sets with controls in Hardy-Weinberg equilibrium, under the homozygous additive genetic model for the minor allele (CC vs. TT).

**
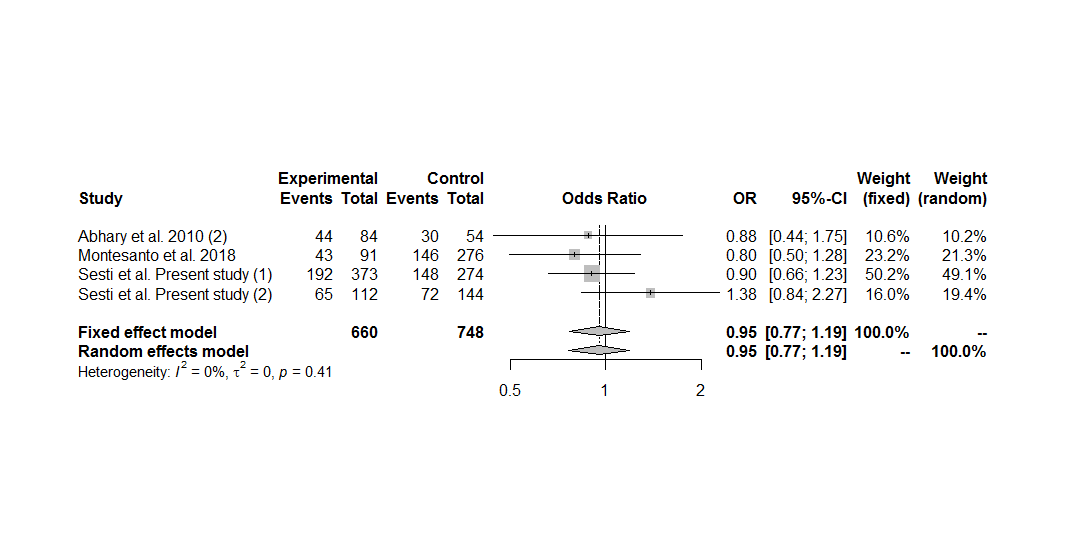
**

**Figure S94.** Forest plot of the association between the *EPO* rs507392 polymorphism and diabetic retinopathy in the overall group analysis including only the sets with controls in Hardy-Weinberg equilibrium, under the heterozygous additive genetic model for the minor allele (TC vs. TT).

**
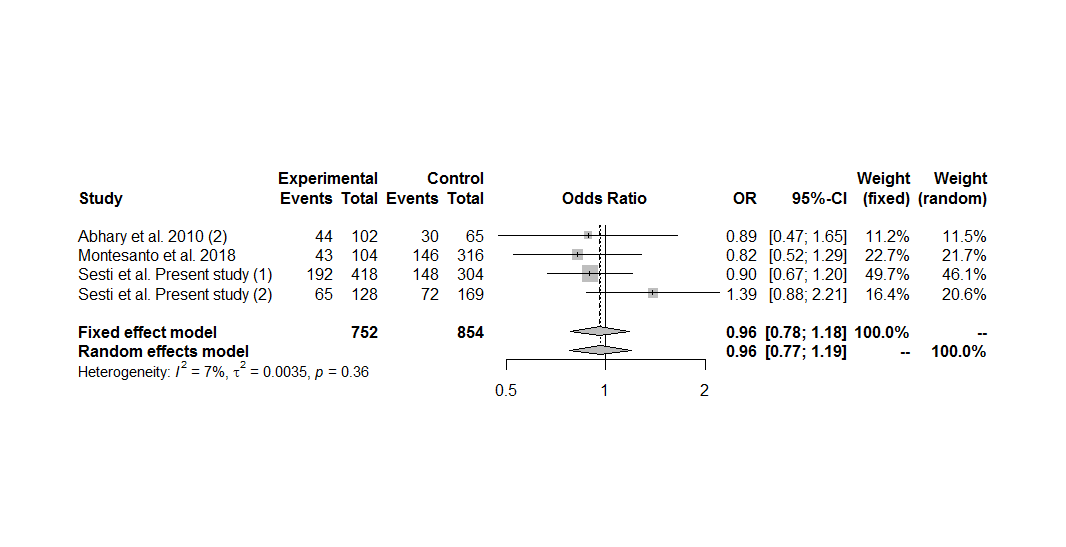
**

**Figure S95.** Forest plot of the association between the *EPO* rs507392 polymorphism and diabetic retinopathy in the overall group analysis including only the sets with controls in Hardy-Weinberg equilibrium, under the overdominant genetic model (TC vs. CC+TT).

**
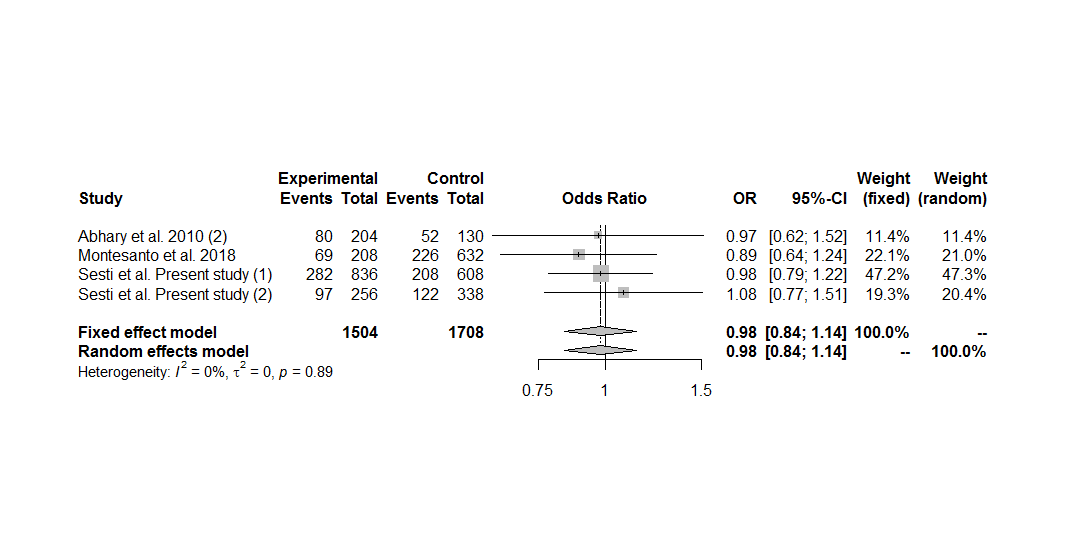
**

**Figure S96.** Forest plot of the association between the *EPO* rs507392 polymorphism and diabetic retinopathy in the overall group analysis including only the sets with controls in Hardy-Weinberg equilibrium, under the allele contrast genetic model (C vs. T).

**
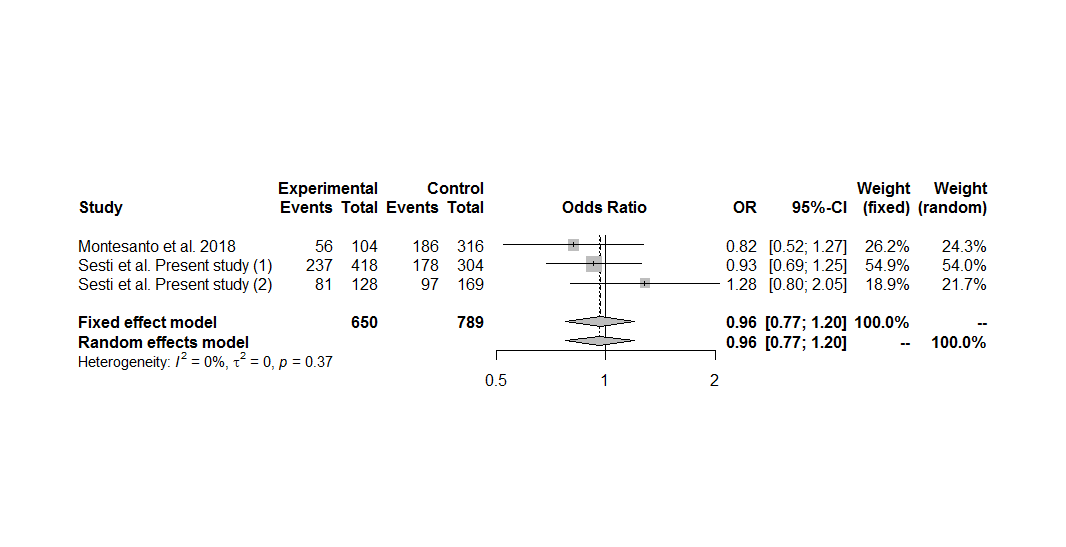
**

**Figure S97.** Forest plot of the association between the *EPO* rs507392 polymorphism and diabetic retinopathy in subjects with type 2 diabetes, including only the sets with controls in Hardy-Weinberg equilibrium, under the dominant genetic model for the minor allele (CC+TC vs. TT).

**
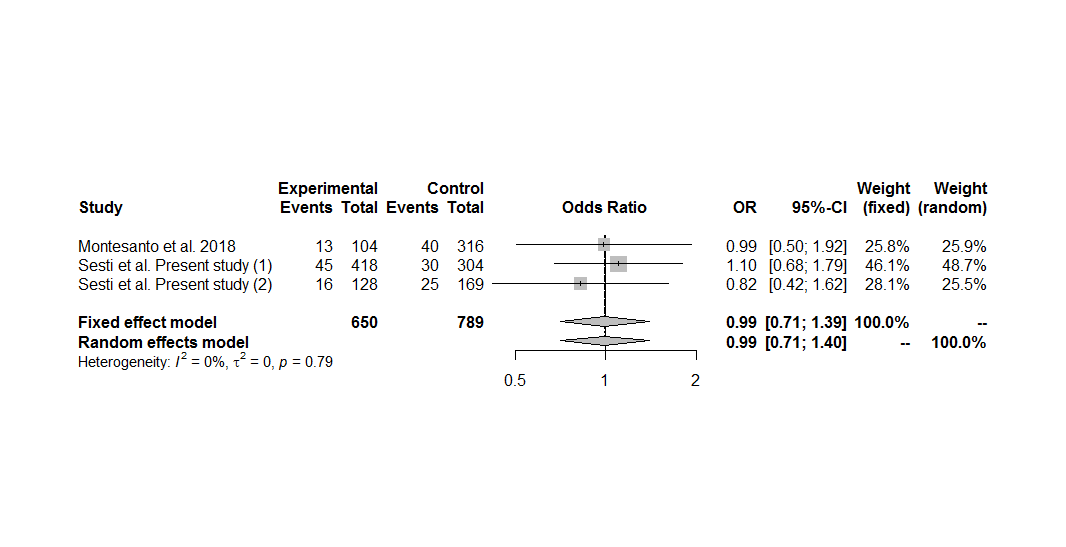
**

**Figure S98.** Forest plot of the association between the *EPO* rs507392 polymorphism and diabetic retinopathy in subjects with type 2 diabetes, including only the sets with controls in Hardy-Weinberg equilibrium, under the recessive genetic model for the minor allele (CC vs. TC+TT).

**
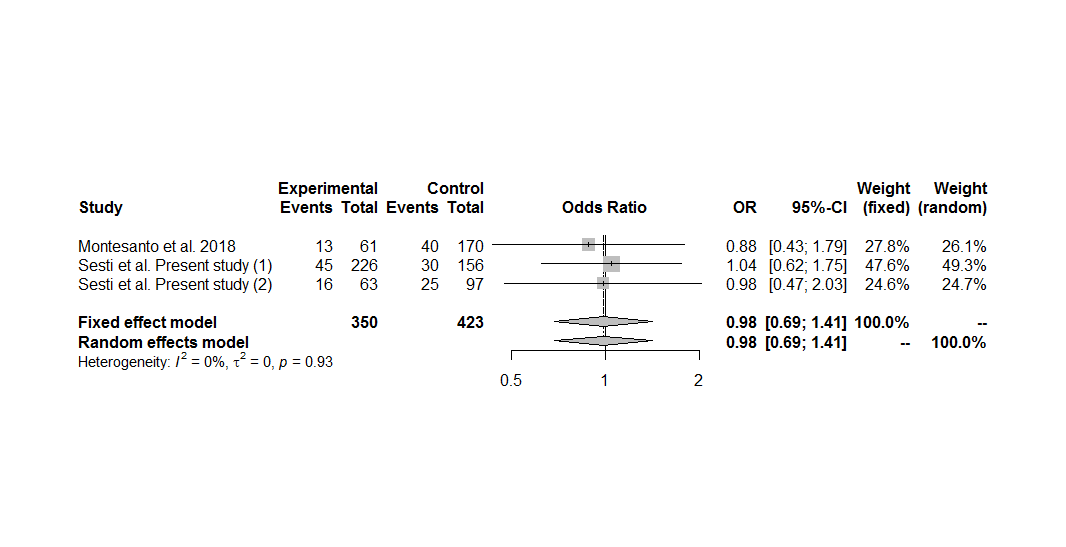
**

**Figure S99.** Forest plot of the association between the *EPO* rs507392 polymorphism and diabetic retinopathy in subjects with type 2 diabetes, including only the sets with controls in Hardy-Weinberg equilibrium, under the homozygous additive genetic model for the minor allele (CC vs. TT).


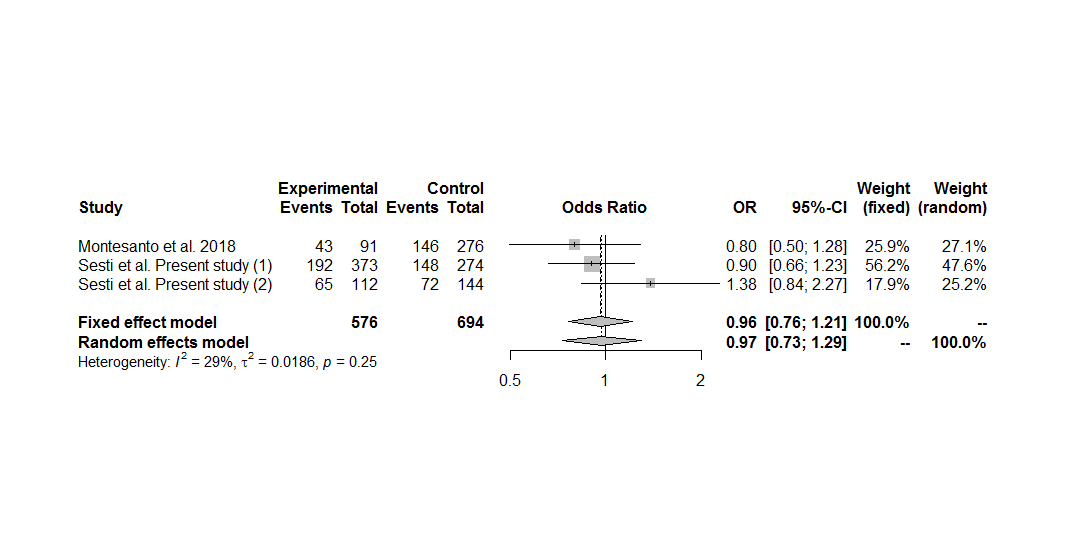


**Figure S100.** Forest plot of the association between the *EPO* rs507392 polymorphism and diabetic retinopathy in subjects with type 2 diabetes, including only the sets with controls in Hardy-Weinberg equilibrium, under the heterozygous additive genetic model for the minor allele (TC vs. TT).

**Figure S101.** Forest plot of the association between the *EPO* rs507392 polymorphism and diabetic retinopathy in subjects with type 2 diabetes, including only the sets with controls in Hardy-Weinberg equilibrium, under the overdominant genetic model (TC vs. CC+TT).

**Figure S102.** Forest plot of the association between the *EPO* rs507392 polymorphism and diabetic retinopathy in subjects with type 2 diabetes, including only the sets with controls in Hardy-Weinberg equilibrium, under the allele contrast genetic model (C vs. T).

**Figure S103.** Forest plot of the association between the *EPO* rs551238 polymorphism and diabetic retinopathy in the overall group analysis, under the dominant genetic model for the minor allele (CC+AC vs. AA).

**Figure S104.** Forest plot of the association between the *EPO* rs551238 polymorphism and diabetic retinopathy in the overall group analysis, under the recessive genetic model for the minor allele (CC vs. AC+AA).

**Figure S105.** Forest plot of the association between the *EPO* rs551238 polymorphism and diabetic retinopathy in the overall group analysis, under the homozygous additive genetic model for the minor allele (CC vs. AA).

**Figure S106.** Forest plot of the association between the *EPO* rs551238 polymorphism and diabetic retinopathy in the overall group analysis, under the heterozygous additive genetic model for the minor allele (AC vs. AA).

**Figure S107.** Forest plot of the association between the *EPO* rs551238 polymorphism and diabetic retinopathy in the overall group analysis, under the overdominant genetic model (AC vs. CC+AA).

**Figure S108.** Forest plot of the association between the *EPO* rs551238 polymorphism and diabetic retinopathy in the overall group analysis, under the allele contrast genetic model (C vs. A).

**Figure S109.** Forest plot of the association between the *EPO* rs551238 polymorphism and diabetic retinopathy in the overall group analysis including only the sets with controls in Hardy-Weinberg equilibrium, under the dominant genetic model for the minor allele (CC+AC vs. AA).

**Figure S110.** Forest plot of the association between the *EPO* rs551238 polymorphism and diabetic retinopathy in the overall group analysis including only the sets with controls in Hardy-Weinberg equilibrium, under the recessive genetic model for the minor allele (CC vs. AC+AA).

**Figure S111.** Forest plot of the association between the *EPO* rs551238 polymorphism and diabetic retinopathy in the overall group analysis including only the sets with controls in Hardy-Weinberg equilibrium, under the homozygous additive genetic model for the minor allele (CC vs. AA).

**Figure S112.** Forest plot of the association between the *EPO* rs551238 polymorphism and diabetic retinopathy in the overall group analysis including only the sets with controls in Hardy-Weinberg equilibrium, under the heterozygous additive genetic model for the minor allele (AC vs. AA).

**Figure S113.** Forest plot of the association between the *EPO* rs551238 polymorphism and diabetic retinopathy in the overall group analysis including only the sets with controls in Hardy-Weinberg equilibrium, under the overdominant genetic model (AC vs. CC+AA).

**Figure S114.** Forest plot of the association between the *EPO* rs551238 polymorphism and diabetic retinopathy in the overall group analysis including only the sets with controls in Hardy-Weinberg equilibrium, under the allele contrast genetic model (C vs. A).

**Figure S115.** Forest plot of the association between the *EPO* rs551238 polymorphism and diabetic retinopathy in subjects with type 2 diabetes, including only the sets with controls in Hardy-Weinberg equilibrium, under the dominant genetic model for the minor allele (CC+AC vs. AA).

**Figure S116.** Forest plot of the association between the *EPO* rs551238 polymorphism and diabetic retinopathy in subjects with type 2 diabetes, including only the sets with controls in Hardy-Weinberg equilibrium, under the recessive genetic model for the minor allele (CC vs. AC+AA).

**Figure S117.** Forest plot of the association between the *EPO* rs551238 polymorphism and diabetic retinopathy in subjects with type 2 diabetes, including only the sets with controls in Hardy-Weinberg equilibrium, under the homozygous additive genetic model for the minor allele (CC vs. AA).

**Figure S118.** Forest plot of the association between the *EPO* rs551238 polymorphism and diabetic retinopathy in subjects with type 2 diabetes, including only the sets with controls in Hardy-Weinberg equilibrium, under the heterozygous additive genetic model for the minor allele (AC vs. AA).

**Figure S119.** Forest plot of the association between the *EPO* rs551238 polymorphism and diabetic retinopathy in subjects with type 2 diabetes, including only the sets with controls in Hardy-Weinberg equilibrium, under the overdominant genetic model (AC vs. CC+AA).

**Figure S120.** Forest plot of the association between the *EPO* rs551238 polymorphism and diabetic retinopathy in subjects with type 2 diabetes, including only the sets with controls in Hardy-Weinberg equilibrium, under the allele contrast genetic model (C vs. A).

**Figure S121.** Forest plot of the association between the *EPO* rs551238 polymorphism and diabetic retinopathy in non-Asians, including only the sets with controls in Hardy-Weinberg equilibrium, under the dominant genetic model for the minor allele (CC+AC vs. AA).

**Figure S122.** Forest plot of the association between the *EPO* rs551238 polymorphism and diabetic retinopathy in non-Asians, including only the sets with controls in Hardy-Weinberg equilibrium, under the recessive genetic model for the minor allele (CC vs. AC+AA).

**Figure S123.** Forest plot of the association between the *EPO* rs551238 polymorphism and diabetic retinopathy in non-Asians, including only the sets with controls in Hardy-Weinberg equilibrium, under the homozygous additive genetic model for the minor allele (CC vs. AA).

**Figure S124.** Forest plot of the association between the *EPO* rs551238 polymorphism and diabetic retinopathy in non-Asians, including only the sets with controls in Hardy-Weinberg equilibrium, under the heterozygous additive genetic model for the minor allele (AC vs. AA).

**Figure S125.** Forest plot of the association between the *EPO* rs551238 polymorphism and diabetic retinopathy in non-Asians, including only the sets with controls in Hardy-Weinberg equilibrium, under the overdominant genetic model (AC vs. CC+AA).

**Figure S126.** Forest plot of the association between the *EPO* rs551238 polymorphism and diabetic retinopathy in non-Asians, including only the sets with controls in Hardy-Weinberg equilibrium, under the allele contrast genetic model (C vs. A).
