# Supplementary material for: Association of polymorphisms in the erythropoietin gene with diabetic retinopathy: a case–control study and systematic review with meta-analysis
Source: BMC Ophthalmol. 2022 Jun 4;22:250. doi: 10.1186/s12886-022-02467-y (PMC9167513; doi:10.1186/s12886-022-02467-y)
Supplement: Supplementary file 5 — Additional file 5: Table S8. Summary of the main differences between our meta-analysis and the previous one by Li et al. [41]. [file 12886_2022_2467_MOESM5_ESM.docx]

**Supplementary Table S8** Summary of the main differences between our meta-analysis and the previous one by Li et al. [41]

|  | **Sesti et al. (present study)** | **Li et al. (2017)** |
| --- | --- | --- |
| Year of publication of the studies included in the final quantitative synthesis* | 2008−2021 | 2010−2015 |
| Genetic models tested | Dominant, recessive, homozygous and heterozygous additive, overdominant, and allele contrast | Dominant, recessive, homozygous and heterozygous additive, and allele contrast |
| Correction for multiple comparisons | Yes (the threshold for statistical significance was set based on Bonferroni correction; i.e., 0.05/number of comparisons) | Not reported |
| Analysis of haplotypes | Yes | No |
| Statistical test(s) used to evaluate small-study effects | Rücker’s test | Begg’s test and Egger’s test |
| Studies included in the final quantitative synthesis for the rs1617640 polymorphism* | Tong et al. (1), Tong et al. (2), Tong et al. (3) (2008), Abhary et al. (T1DM) (2010), Balasubbu et al. (2010), Yang et al. (2014), Song et al. (2015), Montesanto et al. (2018), Mankoc Ramus et al. (2021), Sesti et al. (1), Sesti et al. (2) | Abhary et al. (T1DM) (2010), Balasubbu et al. (2010), Yang et al. (2014), plus two theses for master’s degrees (2014** and 2015) |
| Main findings regarding the rs1617640 polymorphism | The G allele was associated with protection for NPDR under the recessive model. In other subgroup analyses, the G allele was also associated with a decreased risk of DR (PDR+NPDR) among patients with T1DM. | No association was observed |
| Studies included in the final quantitative synthesis for the rs507392 polymorphism* | Abhary et al. (T1DM) (2010), Montesanto et al. (2018), Sesti et al. (1), Sesti et al. (2) | Abhary et al. (T1DM) (2010) |
| Main findings regarding the rs507392 polymorphism | No association was observed | No association was observed |
| Studies included in the final quantitative synthesis for the rs551238 polymorphism* | Abhary et al. (T1DM) (2010), Fan et al. (2016), Montesanto et al. (2018), Sesti et al. (1), Sesti et al. (2) | Abhary et al. (T1DM) (2010), Fan et al. (2016), plus one thesis for master’s degree (2015) |
| Main findings regarding the rs551238 polymorphism | No association was observed | The A allele was associated with decreased risk of DR |

*Studies whose genotype frequencies were in Hardy-Weinberg equilibrium in the control group. **The master’s degree thesis of 2014 was published as an original article by Song et al. (2015). T1DM: type 1 diabetes mellitus
